# Quantitative analysis of th*e* effects of brushing, flossing, and mouthrinsing on supragingival and subgingival plaque microbiota: 12-week clinical trial

**DOI:** 10.1186/s12903-024-04362-y

**Published:** 2024-05-17

**Authors:** Kyungrok Min, Mary Lynn Bosma, Gabriella John, James A. McGuire, Alicia DelSasso, Jeffery Milleman, Kimberly R. Milleman

**Affiliations:** 1 Johnson & Johnson Consumer Inc, 199 Grandview Rd, Skillman, NJ USA; 2 Salus Research, Inc, 1220 Medical Park Drive, Building 4, Fort Wayne, IN USA

**Keywords:** Oral microbiome, Mouthrinse, Listerine, Propidium monoazide, Dental plaque

## Abstract

**Background:**

Translational microbiome research using next-generation DNA sequencing is challenging due to the semi-qualitative nature of relative abundance data. A novel method for quantitative analysis was applied in this 12-week clinical trial to understand the mechanical vs. chemotherapeutic actions of brushing, flossing, and mouthrinsing against the supragingival dental plaque microbiome. Enumeration of viable bacteria using vPCR was also applied on supragingival plaque for validation and on subgingival plaque to evaluate interventional effects below the gingival margin.

**Methods:**

Subjects with gingivitis were enrolled in a single center, examiner-blind, virtually supervised, parallel group controlled clinical trial. Subjects with gingivitis were randomized into brushing only (B); brushing and flossing (BF); brushing and rinsing with Listerine® Cool Mint® Antiseptic (BA); brushing and rinsing with Listerine® Cool Mint® Zero (BZ); or brushing, flossing, and rinsing with Listerine® Cool Mint® Zero (BFZ). All subjects brushed twice daily for 1 min with a sodium monofluorophosphate toothpaste and a soft-bristled toothbrush. Subjects who flossed used unflavored waxed dental floss once daily. Subjects assigned to mouthrinses rinsed twice daily. Plaque specimens were collected at the baseline visit and after 4 and 12 weeks of intervention. Bacterial cell number quantification was achieved by adding reference amounts of DNA controls to plaque samples prior to DNA extraction, followed by shallow shotgun metagenome sequencing.

**Results:**

286 subjects completed the trial. The metagenomic data for supragingival plaque showed significant reductions in Shannon-Weaver diversity, species richness, and total and categorical bacterial abundances (commensal, gingivitis, and malodor) after 4 and 12 weeks for the BA, BZ, and BFZ groups compared to the B group, while no significant differences were observed between the B and BF groups. Supragingival plaque vPCR further validated these results, and subgingival plaque vPCR demonstrated significant efficacy for the BFZ intervention only.

**Conclusions:**

This publication reports on a successful application of a quantitative method of microbiome analysis in a clinical trial demonstrating the sustained and superior efficacy of essential oil mouthrinses at controlling dental plaque compared to mechanical methods. The quantitative microbiological data in this trial also reinforce the safety and mechanism of action of EO mouthrinses against plaque microbial ecology and highlights the importance of elevating EO mouthrinsing as an integral part of an oral hygiene regimen.

**Trial registration:**

The trial was registered on ClinicalTrials.gov on 31/10/2022. The registration number is NCT05600231.

**Supplementary Information:**

The online version contains supplementary material available at 10.1186/s12903-024-04362-y.

## Background

Changes in the structure of microbial communities within the dental plaque biofilm serve as a primary etiological factor in common oral diseases, such as caries and periodontitis [1]. In addition to toothbrushing, controlling the plaque biofilm relies on a variety of adjunctive methods that include mechanical flossing and chemotherapeutic mouthrinses.

Despite limited evidence of efficacy, flossing has been a long-standing recommendation [2] among dental professionals for the mechanical removal of interproximal plaque. In a systematic review and meta-analysis conducted by Worthington et al., there was “low-certainty evidence” to suggest “that flossing, in addition to toothbrushing, may reduce gingivitis (measured by gingival index (GI)) at one month (SMD -0.58, 95% confidence interval (CI) ‐1.12 to ‐0.04; 8 trials, 585 participants), three months or six months. The results for proportion of bleeding sites and plaque were [also] inconsistent (very low‐certainty evidence).” [3].

When used as an adjunct to daily mechanical oral hygiene, an alcohol-containing mouthrinse with a fixed combination of four essential oils (EOs) has a long history of demonstrated clinical reductions in plaque, gingivitis, and gingival bleeding [4, 5] and has performed favorably when compared to flossing in two recent 3-month clinical trials [6, 7]. An alcohol-free EO mouthrinse also performed similarly to an alcohol-containing mouthrinse in 6-month clinical trials [8, 9]. The antimicrobial action of alcohol-containing EO mouthrinses has consistently demonstrated reductions of oral microbes in a variety of oral anatomic locations, including the tongue, cheek, and subgingival crevice [10–13]. These data were derived using well-established, although dated, methodologies, such as bacterial cell culture enumeration [14, 15] and checkerboard DNA-DNA hybridization examining specific bacterial species [16, 17].

More recently, advances in microbial profiling using high throughput DNA sequencing have revealed the presence of over 700 bacterial species in the human oral cavity [18]. These new methods enable highly detailed studies of the oral microbiome, which is essential to more fully understand the role of oral microbes in the pathogenesis of, and therefore the potential prevention of, a variety of oral diseases. Currently, however, there is only partial understanding of how certain mechanical and chemotherapeutic interventions impact the oral microbiome. There are limited quantifiable microbiome data describing time-resolved changes in absolute individual bacterial species abundances, spatiotemporal development of microbial communities, and their clinical relevance on various oral surfaces. This is particularly true of interproximal sites where plaque can remain relatively undisturbed and has a greater diversity of bacteria, including those associated with gingivitis, than more easily accessible areas of the mouth [19, 20].

This clinical trial investigated how flossing and mouthrinses containing a fixed combination of EOs with and without alcohol impact plaque microbiota by generating absolute quantitative microbiome data using a new method of microbiome profiling analysis [21] and viable bacteria enumeration by vPCR. Plaque specimens were spiked with known amounts of exogenous control DNA to enable the quantification of bacterial cell numbers. Further, species identities were carefully annotated and categorized according to their clinical relevance using published literature evidence. The subjects recruited in this trial used floss once daily, mouthrinses twice daily, or a combination of both flossing and mouthrinsing for 12 weeks [22]. This mechanistic study is the first to provide a comprehensive quantification of oral care regimen impacts on the plaque microbiome using clinically relevant microbiological metrics.

## Methods

### Study design

This clinical trial was conducted between April 18, 2022 and July 21, 2022 at Salus Research, Inc. (Fort Wayne, Indiana, USA), an independent clinical research site qualified by the American Dental Association Seal of Acceptance Program. This examiner-blind, controlled, randomized, single-center, and parallel-group clinical trial was conducted in accordance with the principles of the International Council on Harmonization for Good Clinical Practice.

Periodontally healthy subjects and subjects with gingivitis were enrolled separately according to the inclusion and exclusion criteria. All subjects refrained from oral hygiene, food, beverages, and smoking for 8 to 18 h before oral examination of the hard and soft tissues, gingivitis, and plaque. Supragingival plaque was collected for microbiome analysis and subgingival plaque for viable bacteria count using PCR (vPCR) as secondary study endpoints before staining the whole mouth plaque with a disclosing dye. The periodontally healthy cohort participated only in one baseline visit, while subjects with gingivitis progressed through the trial after randomization into one of five intervention groups: [B] brushing only; [BF] brushing and flossing with Reach® Unflavored Waxed Dental Floss (Dr. Fresh LLC, Buena Park, California, USA); [BA] brushing and rinsing with Listerine® Cool Mint® Antiseptic (Johnson & Johnson Consumer Inc, New Jersey, USA); [BZ] brushing and rinsing with Listerine® Cool Mint® Zero Alcohol (Johnson & Johnson Consumer Inc, New Jersey, USA); and [BFZ] brushing, flossing, and rinsing with Listerine® Cool Mint® Zero Alcohol. Complete dental prophylaxis was administered to remove all accessible plaque and calculus. The subjects were given a fluoridated toothpaste (Colgate® Cavity Protection, Colgate-Palmolive Company, NY, USA) and brushed twice daily for 1 timed minute using a standard soft-bristled toothbrush (Colgate® Classic Toothbrush Full Head/Soft Bristles, Colgate-Palmolive Company, NY, USA). Subjects in the flossing groups rinsed their mouth with water after brushing and then flossed once daily. Subjects in the mouthrinse groups rinsed with 20 mL of their assigned mouthrinse for 30 timed seconds twice daily after brushing and flossing or brushing. Primary endpoints were based on clinical gingivitis and plaque assessments and secondary endpoints included supragingival and subgingival plaque microbiome assessments. Supragingival plaque microbiome assessments were completed at baseline before prophylaxis and after 4 and 12 weeks of product intervention, while subgingival plaque vPCR assessments were completed only after 12 weeks of intervention. To ensure compliance, all subjects received an initial training at the clinical site for the correct usage of their assigned products and were subsequently supervised virtually once daily during the weekdays through a video call. Subjects were unsupervised for their second daily use in the evening or on weekends, however, compliance for homecare regimen was monitored through individual diaries and by weighing their assigned toothpaste and mouthrinses at each visit.

### Subject inclusion & exclusion

Healthy adults 18 years of age or older with a minimum of 20 natural teeth with scorable facial and lingual surfaces were included. Requirements for the periodontally healthy subjects were whole-mouth mean scores of: Modified Gingival Index (MGI) [23] ≤ 0.75, Expanded Bleeding Index (EBI) [24] < 3%, and no teeth with periodontal pocket depth (PPD) exceeding 3 mm [25–27]. Requirements for the randomized subjects with gingivitis were evidence of some gingivitis (mild to severe), minimum of 10% bleeding sites based on the EBI, no more than three sites having PPD of 5 mm or any sites exceeding 5 mm, and absence of significant oral soft tissue pathology, advanced periodontitis, and oral appliances, which may interfere with flossing.

Key exclusion criteria included the use of chemotherapeutic oral care products containing triclosan, EOs, cetylpyridinium chloride, stannous fluoride, or chlorhexidine; professional dental prophylaxis 4 weeks before the baseline; use of probiotics within 1 week before baseline or during the study, antibiotics, anti-inflammatory, or anticoagulant therapy within 1 month before baseline or during the study; use of intraoral devices; substance abuse (alcohol, drugs, or tobacco); history of significant adverse effects; allergies or sensitivity against oral hygiene products; pregnancy; significant medical conditions; and participation in any clinical trials within 30 days of the trial.

### Sample size, randomization, and blinding

The sample size for this study was based on power to detect differences based on plaque and gingivitis endpoints. The planned sample size of 50 completed subjects per randomized intervention group provides 95% power to detect a difference between BA or BZ and BF means of 0.34 for Interproximal Mean MGI, given a standard deviation of 0.43, based on a two-sided test at the 2.5% significance level. This sample size also provides greater than 99% power to detect a difference between BA or BZ and BF means of 0.54 for Interproximal Mean Turesky Plaque Index (TPI) [28], given a standard deviation of 0.38. The standard deviation estimates were based on previous three-month studies using the examiners for the current study, and the differences between means are conservative estimates based on previous studies of this type. Sample sizes were estimated using PASS version 14.0.4 (NCSS, LLC, Kaysville, UT, USA). Assuming a 5% drop-out rate, the trial recruited 54 subjects per group or 270 subjects with gingivitis to ensure that the trial would be completed with at least 250 subjects in the randomized intervention groups. An additional 30 subjects, representing the non-randomized and periodontally healthy reference group, were recruited for a baseline assessment only.

The randomization schedule for subjects with moderate gingivitis was generated using a validated program created by the Biostatistics Department at Johnson & Johnson Consumer Inc. (Skillman, NJ, USA). The subjects with gingivitis were randomized in an equal allocation using a block size of ten and were assigned a unique randomization number that determined the sequential assignment of intervention products at the baseline visit. To minimize bias, the principal investigator and examiners were blinded to the administered intervention products, while the clinical personnel dispensing them were excluded from subject examinations.

### Oral examination

All clinical assessments in this trial were performed by the same dental examiners. One examiner performed the oral hard and soft tissue assessments, MGI grading, and selection of teeth (as described below) to be sampled. Another examiner performed EBI and TPI grading. Both examiners were trained and calibrated with the visual assessment of gingival inflammation, supragingival plaque, and gingival bleeding as measured using the MGI, TPI, and EBI. All examinations were conducted in the following order: an oral hard and soft tissue assessment, MGI, supragingival and subgingival plaque sampling, EBI, and TPI.

### Plaque sample collection

Plaque samples were collected by the same dental hygienist from the same four teeth selected at baseline, which met the inclusion and exclusion criteria for periodontally healthy subjects or subjects with gingivitis. The preferential teeth numbers were 3, 7, 18, and 23. Adjacent teeth that met the selection criteria were substituted for missing teeth.

Supragingival plaque for microbiome analysis was collected at all visits by moving a sterile curette five strokes supragingivally from the mesiobuccal line angle to follow the gingival margin to interproximal, from the distobuccal line angle to interproximal, and then repeating on the lingual side. Subgingival plaque for vPCR analysis was collected at week 12, during the last visit, using a sterile 204-sickle scaler to enter the interproximal subgingival space, removing plaque within one stroke, and repeating on all buccal and lingual interproximal surfaces. For each individual subject, the supragingival plaque and subgingival plaque samples were pooled, placed separately in 250 µL of sterile ultrapure grade phosphate-buffered saline with pH 7.2, and stored at -80^o^C.

### Shotgun metagenomic sequencing

Microbiome analysis of supragingival plaque was performed using next-generation DNA sequencing at CosmosID, Inc. (Germantown, Maryland, USA). DNA isolation, library preparation, and sequencing were carried out according to vendor-optimized protocol. Briefly, ZymoBIOMICS Spike-in Control II (Zymo Research, Irvine, CA) was added to plaque specimens to enable bacterial cell number quantification. To enhance cell lysis, plaque samples were incubated with MetaPolyzyme at 35 °C for 12 h, and DNA was extracted using ZymoBIOMICS DNA MicroPrep with bead-beating according to the manufacturer’s instructions. DNA concentrations were determined using the Qubit dsDNA HS assay and Qubit 4 fluorometer (ThermoFisher Scientific, Waltham, MA). DNA libraries were prepared using 1 ng of input genomic DNA that was fragmented, amplified, and indexed employing the Nextera XT DNA Library Preparation and Nextera Indexing Kit (Illumina, San Diego, CA). DNA libraries were purified using AMPure magnetic beads (Beckman Coulter, Brea, CA) and then normalized for equimolar pooling. Sequencing was performed using a HiSeq sequencer (Illumina), targeting a coverage of 3 − 4 million paired-end 2 × 150 bp reads.

### Viability qPCR

Quantification of live bacteria from supragingival and subgingival plaque samples was performed at week 12 using vPCR at Azenta Life Sciences, Inc. (South Plainfield, NJ). Plaque samples were treated with PMAxx™ dye (Biotium, San Francisco, CA) soon after their collection to a final concentration of 100 µM, followed by photolysis with blue light for 15 min to inactivate dead bacterial cell DNA. Excess dye was neutralized using Tris-Cl buffer to a final concentration of 5 mM, followed by another cycle of photolysis. After standard DNA extraction, vPCR was performed using a vendor-optimized protocol based on SYBR GREEN chemistry. Target detection included total bacteria using the 16S rRNA universal primer pair 5’-GTGSTGCAYGGYTGTCGTCA-3’ and 5’-ACGTCRTCCMCACCTTCCTC-3’; *Actinomyces oris*, using the 16S rRNA primer pair 5’-TCGACCTGATGGACGTTTCGC-3’ and 5’-ACGGTTGGCATCGTCGTGTT-3’; *Fusobacterium nucleatum*, using the *RpoB* primer pair 5’-GGTTCAGAAGTAGGACCGGGAGA-3’ and 5’-ACTCCCTTAGAGCCATGAGGCAT-3’; and *Porphyromonas gingivalis*, using *RpoB* primer pair 5’-TTGCTGGTTCTGGATGAGTG-3’ and 5’-CAGGCACAGAATATCCCGTATTA-3’.

### Microbiome computational analysis

Raw DNA sequence reads were processed and quality filtered by CosmosID. Bacterial diversity analyses were performed using R statistical programming language version 3.6.1 [29]. Alpha-diversity was assessed using the *vegan* package version 2.5.6 [30] and included observed richness and Shannon-Weaver diversity indices at the species taxonomic level. Statistical comparisons between the treatment groups were evaluated using mixed effects model for repeated measures with baseline covariate and terms for treatment, visit, treatment-by-visit, and baseline-by-visit, and unstructured within-subject covariance. Based on this model, pair-wise comparisons were tested, each at the 5% significance level, two-sided, between each mouthrinse containing group and floss containing group with B and between each mouthrinse containing group with BF. Statistical significance between the healthy and gingivitis cohorts was tested at the 5% significance level, two-sided, using two-sample *t*-test assuming unequal variance.

Beta-diversity analysis was performed using the *phyloseq* package version 1.28.0 [31] to calculate the phylogenetic distance matrix by weighted UniFrac [32] and ordination using principal coordinate analysis. The input phylogenetic tree was constructed using GenBank Common Tree based on the data taxonomy table. Significance testing of factors and interactions that affect bacterial compositions was performed with permutational multivariate analysis of variance (PERMANOVA) [33] using *adonis* in the *vegan* package [30].

For bacterial abundance quantification, standard calibration curves of reference control DNA were evaluated for individual samples [21]. The DNA amounts of bacterial species were calculated using the linear regression of added amounts of reference control DNA vs. output relative abundances and genome molecular weights specific for each bacterial species from GenBank [34]. The resulting bacterial abundances were expressed in units of calculated microbial units (CMUs) and represented in base 10 log where appropriate.

For the quantitative assessment of product intervention, bacterial species were classified into specific categories based on their association with oral conditions. These included commensal, malodor, gingivitis, and acidogenic bacterial groups. The classification was based on a review of the primary scientific literature, including journal research articles and clinical research reports as well as annotations from the Human Oral Microbiome Database [35]. The abundance of bacterial species associated with these different categories was log10-transformed and aggregated per sample basis, and the means of log10 values from all samples were reported.

## Results

### Study group characteristics

**Fig. 1:**
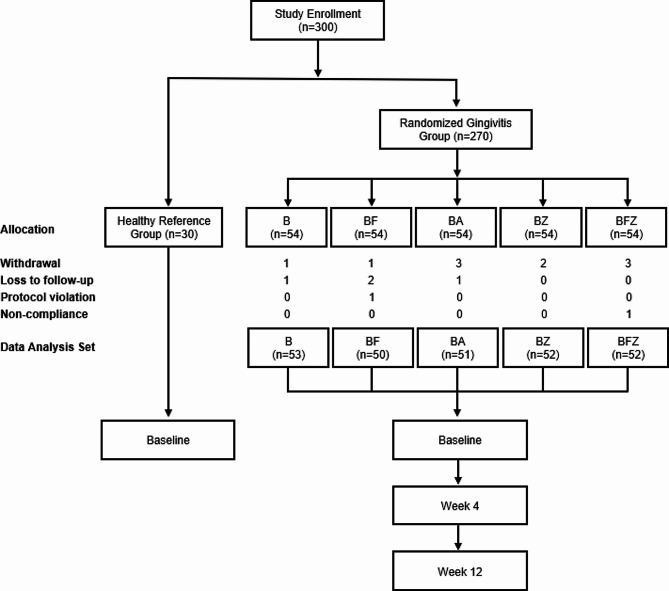
Study design flow chart and subject recruitment

A summary of subject recruitment and a list of baseline demographic and oral health parameters are presented in Fig. 1; Table 1. This trial enrolled 300 generally healthy adults, of which 16 discontinued. For full data analysis, 288 subjects were evaluated including those that partially completed the study with primary and secondary evaluations performed at baseline and at least one post-baseline visit: 30 subjects were in good periodontal health, whereas 256 had gingivitis and were randomized into five treatment arms: 53 in the brushing only group (B); 50 in the brushing and flossing group (BF); 51 in the brushing and rinsing with Listerine® Cool Mint® Antiseptic group (BA); 52 in the brushing and rinsing with Listerine® Cool Mint® Zero Alcohol group (BZ); and 52 in the brushing, flossing, and rinsing with Listerine® Cool® Mint Zero Alcohol group (BFZ). All treatments in this trial were well tolerated. The mean (SD) ages of the healthy subjects and subjects with gingivitis were 52.0 (16.2) years and 43.5 (14.0) years, respectively, with the majority of study participants being females (78.6%), Caucasian (88.2%), and non-smokers (97.5%). The whole-mouth and interproximal baseline oral health parameters were significantly distinct between the healthy and gingivitis cohorts, as expected based on the subject inclusion criteria, with approximately 0.742 vs. 2.675 for the MGI, 2.592 vs. 3.107 for the TPI, 0.012 vs. 0.326 for the EBI, and 0.869 vs. 2.186 for the PPD (*p-*values < 0.001).

**Table 1:** Subject demographic and baseline characteristics

Parameters	Healthy	B	BF	BA	BZ	BFZ
	(*n* = 30)	(*n* = 53)	(*n* = 50)	(*n* = 51)	(*n* = 52)	(*n* = 52)
Mean age (range)	52.0 (18–76)	42.8 (18–76)	44.7 (18–72)	44.4 (18 − 72)	44.0 (23 − 70)	41.04 (18–77)
Sex, n (%)						
Male	3 (10.0)	10 (18.9)	12 (24.0)	6 (11.8)	15 (28.8)	15 (28.8)
Female	27 (90.0)	43 (81.1)	38 (76.0)	45 (88.2)	37 (71.2)	37 (71.2)
Race, n (%)						
White	27 (90.0)	47 (88.7)	43 (86.0)	44 (86.3)	48 (94.2)	45 (86.5)
Black	3 (10.0)	5 (9.4)	7 (14.0)	7 (13.7)	4 (7.8)	7 (13.5)
Asian	0	1 (1.9)	0	0	0	0
Smoker, n (%)						
No	29 (96.7)	52 (98.1)	50 (100.0)	49 (96.1)	52 (100.0)	49 (94.2)
Whole-mouth						
mean MGI ± SD	0.594 ± 0.076	2.560 ± 0.305	2.578 ± 0.270	2.563 ± 0.320	2.653 ± 0.288	2.633 ± 0.261
mean TPI ± SD	2.469 ± 0.371	3.068 ± 0.403	3.133 ± 0.407	2.980 ± 0.468	3.001 ± 0.364	3.031 ± 0.432
mean EBI ± SD	0.018 ± 0.016	0.308 ± 0.187	0.343 ± 0.174	0.304 ± 0.169	0.366 ± 0.195	0.319 ± 0.186
mean PPD ± SD	1.731 ± 0.234	2.048 ± 0.319	2.048 ± 0.307	1.998 ± 0.329	2.122 ± 0.395	2.068 ± 0.334
Interproximal						
mean MGI ± SD	0.889 ± 0.104	2.738 ± 0.269	2.744 ± 0.225	2.722 ± 0.286	2.796 ± 0.226	2.785 ± 0.208
mean TPI ± SD	2.715 ± 0.332	3.200 ± 0.382	3.275 ± 0.369	3.130 ± 0.413	3.138 ± 0.329	3.178 ± 0.386
mean EBI ± SD	0.005 ± 0.008	0.305 ± 0.191	0.342 ± 0.176	0.296 ± 0.178	0.367 ± 0.206	0.311 ± 0.186
mean PPD ± SD	0.007 ± 0.020	2.309 ± 0.328	2.290 ± 0.312	2.242 ± 0.344	2.386 ± 0.404	2.312 ± 0.340

### Bacterial profiling of supragingival plaque

Metagenomic sequencing of supragingival plaque identified a total of 574 unique taxa at the species level (Table 2). Extensive clinical and scientific literature reviews of species identities helped to classify these taxa with clinical relevance (Additional File 1). At the study level, 236 species were identified as belonging to the human oral cavity, 228 were identified as transient or extraoral, and the remaining 109 were unknown or unclassified. At the individual subject level, there were, on average, 155 distinct species, of which 120 were identified as oral residents, nine were found to be transient or extraoral, and 26 were unknown or unclassified. While certain oral bacterial species overlapped across different categories, approximately 91 were commensal, whereas 28 were associated with gingivitis, 16 with malodor, and six with acidogenesis. No statistically significant differences in the species classification were observed between the healthy and gingivitis cohorts (Table 2, p*-*values > 0.512).

**Table 2:** Summary of bacterial profiling showing the number of species in each classification at baseline. Mean values for the healthy vs. gingivitis cohorts are rounded to the nearest integer. *p-*values represent two-sample *t-*test comparing healthy subjects vs. subjects with gingivitis

Classification	Total taxa in the study	Mean taxa Healthy ± SD	Mean taxa Gingivitis ± SD	*p*-values
Distinct Bacterial Species	574	156 ± 41	153 ± 41	0.611
Oral Bacterial Species	236	122 ± 34	118 ± 33	0.591
Commensal	158	92 ± 23	89 ± 21	0.557
Gingivitis	67	28 ± 12	27 ± 13	0.632
Malodor	30	16 ± 6	15 ± 6	0.775
Acidogenic	21	6 ± 2	6 ± 2	0.512
Unknown or Unclassified	109	26 ± 6	25 ± 6	0.555
Transient or Extraoral	228	9 ± 4	9 ± 5	0.784

### Healthy vs. gingivitis supragingival plaque microbiota

**Fig. 2:**
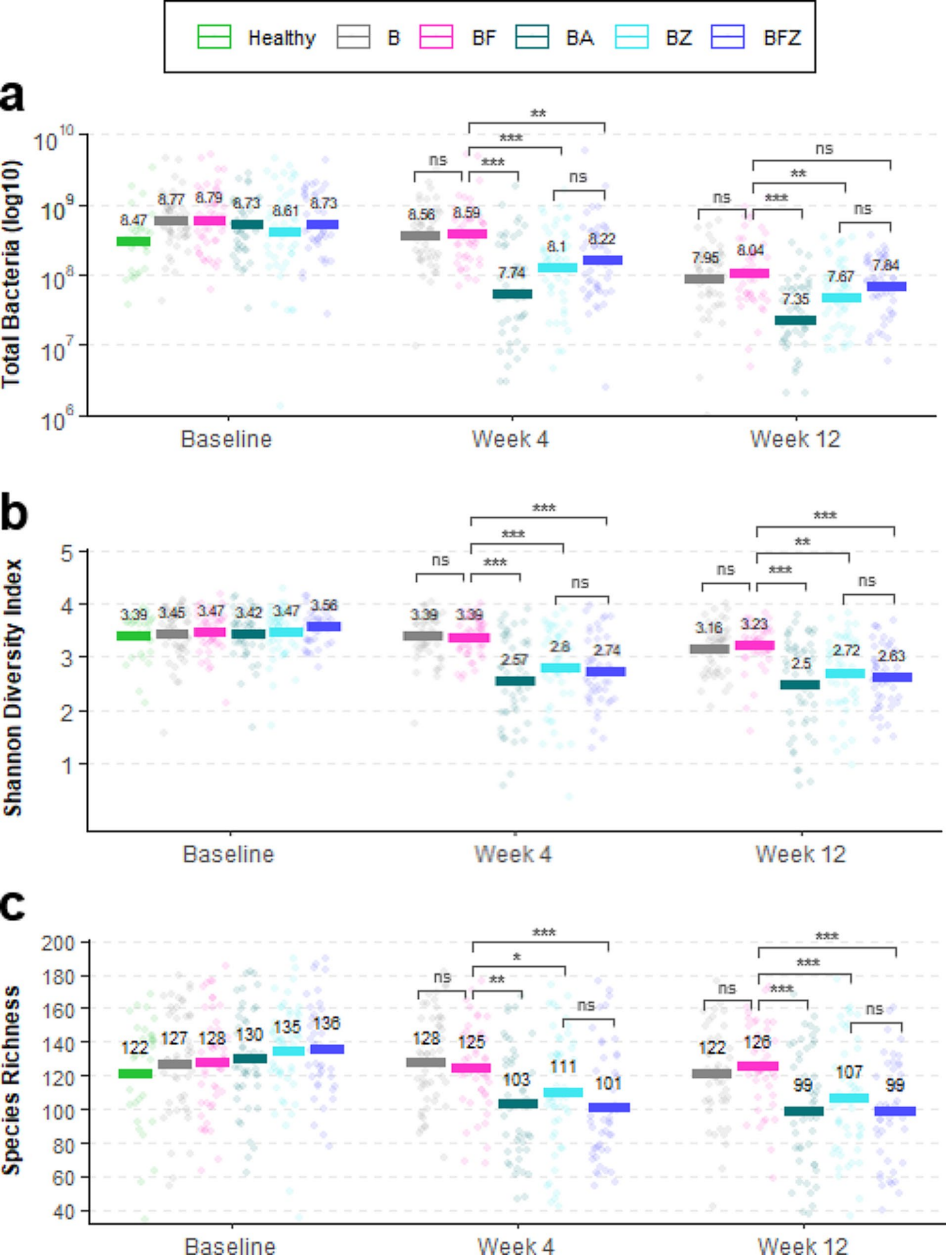
Microbiome assessment of supragingival plaque. The means of (**a**) total oral bacteria abundance in log10 CMU, (**b**) Shannon-Weaver diversity index, and (**c**) observed species richness are shown. Dots represent individual samples. ns = not significant, *p < 0.05, **p < 0.01, ***p < 0.001

**Fig. 3:**
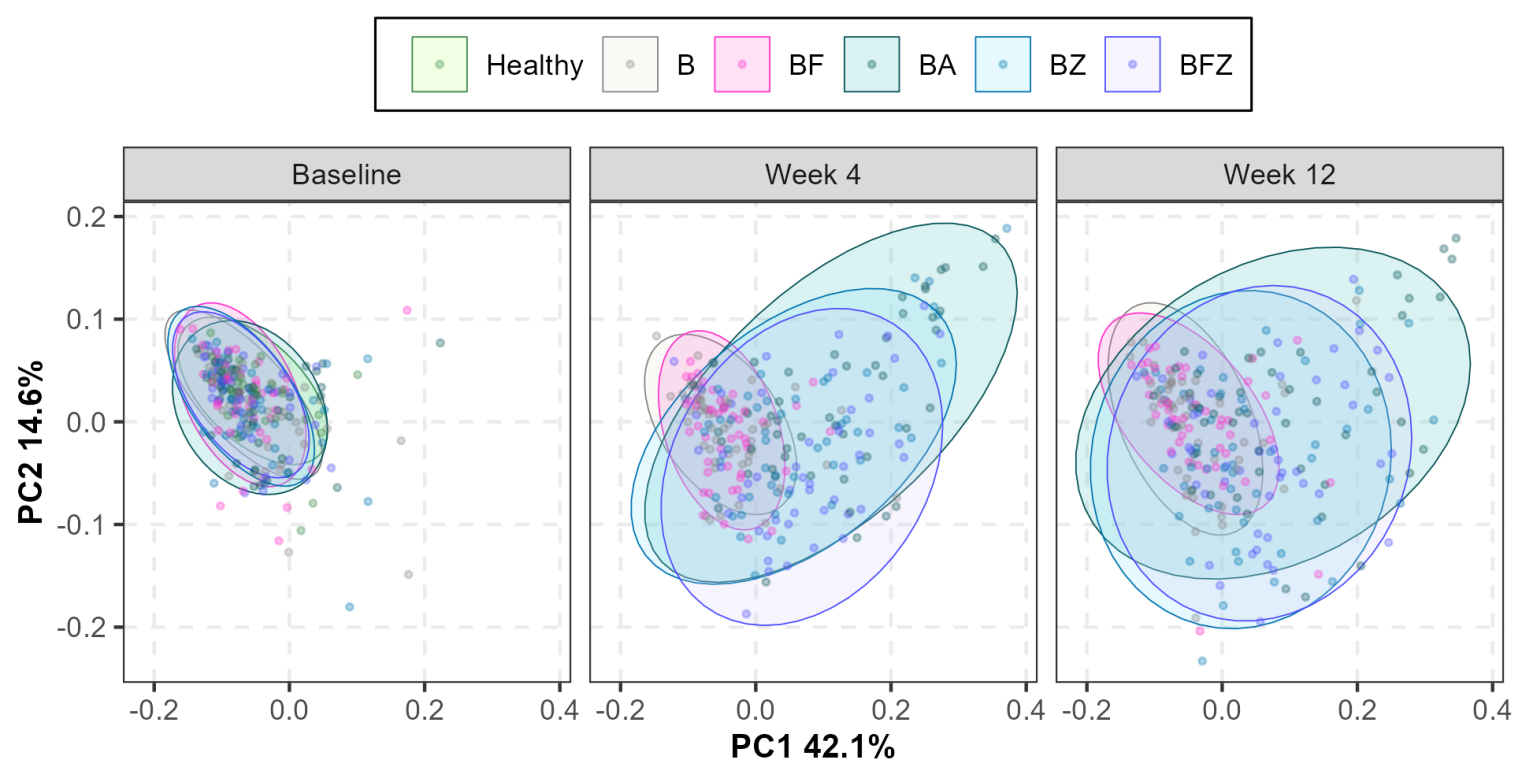
Weighted UniFrac principal coordinate analysis demonstrating time-resolved changes in the beta-diversity of the supragingival plaque microbiome after 4 weeks and 12 weeks of oral care regimen

Despite significant differences in the mean demographic age (*p* = 0.012) and clinical oral health parameters between the periodontally healthy and gingivitis cohorts (Table 1), microbiome analysis of supragingival plaque at subject recruitment showed no statistically significant differences in α-diversity measures, such as the Shannon-Weaver Diversity Index (Fig. 2b, *p =* 0.336) or observed species richness (Fig. 2c, *p =* 0.147), as well as β-diversity using weighted UniFrac PCoA analysis (Fig. 3 Baseline Visit). This compositional similarity coincided with the baseline whole-mouth and interproximal mean TPI scores showing the least amount of differentiation (Table 1, Δ = 0.5) compared to MGI or EBI (Table 1, Δ = 2 or 3). Quantification of total plaque bacteria, however, showed that healthy subjects had significantly lower abundances compared to subjects with gingivitis (Fig. 2a, *p* = 0.012). A detailed low-level comparison of individual bacteria demonstrated that 36 species were significantly more abundant in subjects with gingivitis than in healthy subjects (Table 3).

**Table 3:** Supragingival plaque bacteria with significant differences in abundances between subjects with gingivitis (*n* = 258) and the naturally healthy reference cohort (*n* = 30) at baseline. Abundance values are means of log_10_ CMUs ± SD, whereas *p-*values represent two-sample Wilcoxon rank sum test

Species	Healthy Subjects	Gingivitis Subjects	Difference	*p*-values
*Abiotrophia defectiva*	4.020 ± 2.968	5.207 ± 2.767	1.188	0.002
*Actinomyces dentalis*	6.064 ± 1.912	6.735 ± 1.429	0.671	0.015
*Actinomyces naeslundii*	6.654 ± 0.752	7.041 ± 0.687	0.388	0.004
*Actinomyces oris*	6.981 ± 0.571	7.289 ± 0.554	0.308	0.003
*Actinomyces viscosus*	7.129 ± 0.532	7.383 ± 0.562	0.254	0.009
*Alloprevotella tannerae*	2.146 ± 2.745	3.371 ± 2.933	1.225	0.025
*Atopobium parvulum*	3.662 ± 2.707	4.556 ± 2.605	0.894	0.034
*Campylobacter curvus*	0.629 ± 1.635	1.802 ± 2.425	1.174	0.011
*Campylobacter gracilis*	5.391 ± 1.238	5.637 ± 1.644	0.246	0.005
*Capnocytophaga endodontalis*	5.596 ± 1.219	5.948 ± 1.020	0.351	0.039
*Capnocytophaga ochracea*	5.893 ± 1.358	6.231 ± 1.441	0.337	0.008
*Fusobacterium hwasookii*	2.508 ± 2.466	3.456 ± 2.299	0.948	0.037
*Fusobacterium nucleatum*	4.815 ± 1.538	5.324 ± 1.322	0.509	0.013
*Granulicatella adiacens*	6.289 ± 1.354	6.742 ± 0.899	0.452	0.011
*Lachnoanaerobaculum saburreum*	4.674 ± 2.309	5.726 ± 1.678	1.052	0.003
*Leptotrichia buccalis*	3.646 ± 2.365	4.997 ± 2.126	1.351	< 0.001
*Leptotrichia goodfellowii*	1.203 ± 2.224	2.483 ± 2.603	1.281	0.015
*Leptotrichia hofstadii*	4.694 ± 2.272	5.566 ± 1.779	0.872	0.008
*Leptotrichia massiliensis*	4.029 ± 2.157	5.118 ± 1.990	1.089	< 0.001
*Leptotrichia shahii*	3.321 ± 2.339	4.621 ± 2.010	1.3	< 0.001
*Leptotrichia trevisanii*	3.743 ± 2.020	4.602 ± 1.826	0.859	< 0.001
*Leptotrichia wadei*	4.344 ± 2.384	5.409 ± 1.967	1.065	0.004
*Prevotella melaninogenica*	3.249 ± 2.754	4.375 ± 2.430	1.126	0.031
*Prevotella oralis*	0.195 ± 1.070	1.156 ± 2.226	0.961	0.02
*Streptococcus gordonii*	5.853 ± 0.844	6.372 ± 0.980	0.518	0.001
*Streptococcus halitosis*	5.393 ± 0.663	5.669 ± 0.940	0.277	0.008
*Streptococcus intermedius*	4.549 ± 2.282	5.253 ± 1.965	0.703	0.049
*Streptococcus mitis*	5.870 ± 0.758	6.114 ± 0.986	0.244	0.026
*Streptococcus mutans*	1.368 ± 2.808	2.814 ± 3.321	1.445	0.03
*Streptococcus sanguinis*	6.374 ± 0.832	6.657 ± 0.785	0.283	0.04
*Streptococcus sinensis*	3.999 ± 1.889	4.689 ± 1.367	0.691	0.008
*Streptococcus symci*	3.820 ± 1.794	4.274 ± 1.737	0.453	0.015
*Veillonella dispar*	6.922 ± 0.571	7.110 ± 1.068	0.188	0.007
*Veillonella infantium*	3.981 ± 1.903	4.370 ± 1.842	0.389	0.04
*Veillonella parvula*	6.930 ± 0.580	7.158 ± 0.938	0.228	0.007
*Veillonella tobetsuensis*	1.193 ± 2.039	2.207 ± 2.431	1.014	0.025

### Impact of the oral care regimen on supragingival plaque

**Fig. 4:**
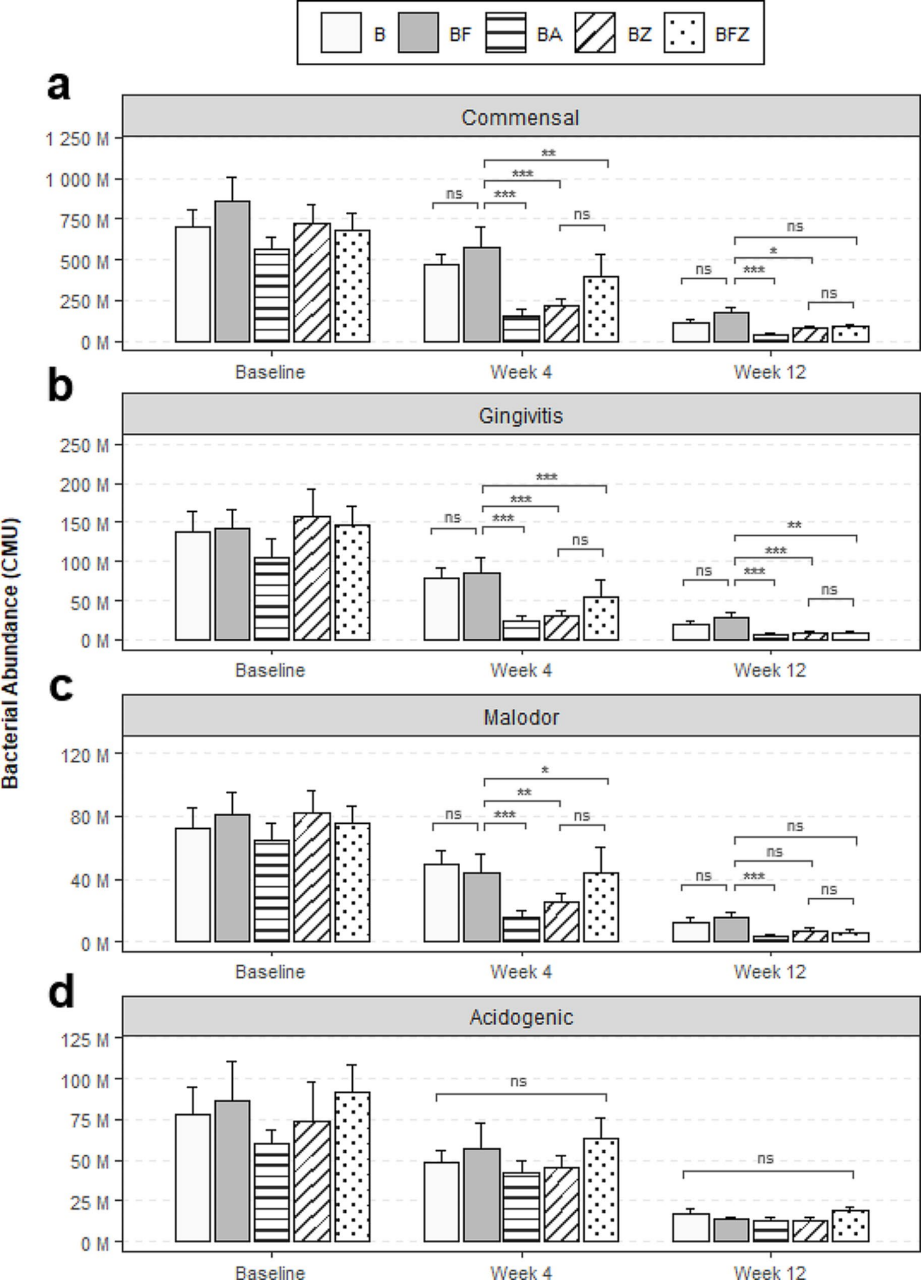
Impact of the oral care regimen on the supragingival plaque microbiome. The mean abundances of bacterial species that are (**a**) oral commensal, (**b**) associated with gingivitis, (**c**) producing volatile sulfur compounds, and (**d**) acidogenic are shown. Error bars represent the standard error of the mean. ns = not significant, *p < 0.05, **p < 0.01, ***p < 0.001

Quantitative analysis of supragingival plaque collected from subjects with gingivitis revealed significant differences between the mechanical and chemotherapeutic actions of oral care regimen after 4 weeks and 12 weeks. Specifically, compared to B, BF had no effects on Shannon-Weaver Diversity, observed species richness, total bacteria abundance, and β-diversity assessed by weighted UniFrac, showing lack of antimicrobial control against supragingival plaque (Figs. 2 and 3, BF vs. B). Further detailed analyses demonstrated that BF had no effects against commensal, gingivitis, malodor, or acidogenic groups of bacteria (Fig. 4, BF vs. B). Moreover, at the individual species level, there were no significant differences in bacterial abundances between the BF and B groups except for 11 commensal species, which increased in abundance after 12 weeks (Table 4, BF vs. B). The clinical endpoint measures for plaque also showed no statistically significant differences between B and BF [22] using interproximal mean TPI at week 4 (*p =* 0.696) and at week 12 (*p =* 0.164) and whole-mouth mean TPI at week 4 (*p =* 0.430) and at week 12 (*p =* 0.229).

**Table 4:** Supragingival plaque bacteria with significant differences in abundances after a product intervention for 12 weeks. Abundance values are the means of log_10_ CMUs. Only significant values compared to brushing (*p ≤* 0.05) are shown based on two-sample Wilcoxon rank sum test. n.s. – not significant

Species	Association	B	BF	BA	BZ	BFZ
*Actinomyces dentalis*	Gingivitis	5.319	n.s.	3.975	4.732	4.167
*Actinomyces israelii*		4.351	n.s.	3.14	3.363	3.75
*Aggregatibacter actinomycetemcomitans*		0.776	n.s.	0.191	n.s.	0.136
*Campylobacter rectus*		3.766	n.s.	2.66	n.s.	2.799
*Campylobacter showae*		4.395	n.s.	3.38	n.s.	n.s.
*Cardiobacterium hominis*		5.835	n.s.	4.097	5.108	5.061
*Cardiobacterium valvarum*		4.438	n.s.	2.772	n.s.	3.362
*Cryptobacterium curtum*		0.84	0.222	n.s.	n.s.	0.226
*Dialister invisus*		3.953	n.s.	3.018	n.s.	2.644
*Eikenella corrodens*		4.957	n.s.	3.481	4.352	4.247
Fusobacterium hwasookii		2.306	*n*.s.	1.143	1.384	1.11
*Fusobacterium nucleatum*		3.935	n.s.	3.006	3.322	2.829
*Lachnoanaerobaculum gingivalis*		2.709	n.s.	1.672	n.s.	n.s.
*Lautropia dentalis*		3.562	n.s.	1.152	2.65	2.312
*Leptotrichia buccalis*		3.424	n.s.	2.109	1.806	1.879
*Leptotrichia hongkongensis*		4.404	n.s.	2.444	3.206	3.547
*Leptotrichia shahii*		3.114	n.s.	1.842	1.775	1.834
*Peptidiphaga gingivicola*		3.472	n.s.	2.083	1.694	2.057
*Porphyromonas gingivalis*		1.618	n.s.	0.867	n.s.	0.635
*Prevotella loescheii*		3.15	n.s.	1.818	2.33	1.65
*Pseudopropionibacterium propionicum*		5.057	n.s.	3.422	3.788	3.474
*Selenomonas noxia*		5.286	n.s.	3.572	4.262	4.205

*Actinomyces odontolyticus*	Malodor	5.366	n.s.	3.381	4.852	n.s.
*Atopobium parvulum*		3.618	n.s.	2.597	n.s.	n.s.
*Centipeda periodontii*		2.317	n.s.	1.436	1.347	1.238
*Fusobacterium periodonticum*		3.151	n.s.	1.727	n.s.	2.119
*Megasphaera micronuciformis*		2.478	n.s.	0.885	n.s.	n.s.
Prevotella melaninogenica		4.134	*n*.s.	1.905	3.037	3.015
*Prevotella pallens*		2.74	n.s.	1.199	1.244	1.614
*Prevotella shahii*		1.8	n.s.	0.942	n.s.	0.715
*Streptococcus halitosis*		4.561	n.s.	3.983	n.s.	n.s.
*Veillonella dispar*		6.148	n.s.	5.336	n.s.	n.s.

*Bifidobacterium dentium*	Acidogenic	0.303	n.s.	0.749	n.s.	n.s.
*Lactobacillus fermentum*		0.498	0	n.s.	0	n.s.
Prevotella histicola		2.384	*n*.s.	1.615	1.359	*n*.s.
*Scardovia wiggsiae*		2.425	n.s.	1.342	1.358	n.s.

*Abiotrophia defectiva*	Commensal	2.873	n.s.	1.29	1.245	0.85
*Actinomyces gerencseriae*		5.156	n.s.	4.123	n.s.	n.s.
*Actinomyces graevenitzii*		2.101	n.s.	0.782	n.s.	n.s.
*Actinomyces johnsonii*		5.764	n.s.	4.709	5.368	5.386
*Actinomyces massiliensis*		5.746	n.s.	4.464	5.58	n.s.
*Actinomyces naeslundii*		6.197	n.s.	5.191	n.s.	n.s.
*Actinomyces oris*		6.594	n.s.	6.024	n.s.	n.s.
*Actinomyces slackii*		0.597	n.s.	0.108	n.s.	n.s.
*Actinomyces timonensis*		3.715	n.s.	2.58	n.s.	n.s.
*Actinomyces viscosus*		6.569	n.s.	6.219	n.s.	6.855
*Aggregatibacter aphrophilus*		3.899	n.s.	2.133	3.114	2.68
*Aggregatibacter segnis*		4.312	n.s.	2.357	3.37	2.752
*Campylobacter concisus*		3.666	n.s.	2.532	n.s.	n.s.
*Campylobacter gracilis*		4.789	n.s.	3.94	n.s.	n.s.
*Capnocytophaga endodontalis*		4.927	n.s.	3.562	n.s.	4.223
*Capnocytophaga gingivalis*		5.592	n.s.	4.586	5.287	n.s.
*Capnocytophaga granulosa*		4.772	n.s.	3.418	3.96	3.896
*Capnocytophaga ochracea*		5.37	n.s.	3.936	4.5	4.359
*Capnocytophaga sputigena*		5.16	n.s.	3.847	4.54	4.614
*Corynebacterium durum*		6.045	n.s.	4.48	5.748	5.549
*Corynebacterium matruchotii*		6.487	n.s.	4.892	5.716	5.973
*Gemella morbillorum*		4.531	n.s.	2.985	4.028	3.228
*Gemella sanguinis*		3.546	n.s.	2.593	n.s.	2.689
*Granulicatella adiacens*		5.508	n.s.	4.342	4.782	4.542
*Granulicatella elegans*		0.774	1.614	n.s.	n.s.	n.s.
*Haemophilus haemolyticus*		3.394	n.s.	2.386	2.965	2.54
*Haemophilus influenzae*		3.205	n.s.	1.851	n.s.	n.s.
*Haemophilus parainfluenzae*		5.285	6.057	4.087	n.s.	n.s.
*Haemophilus pittmaniae*		1.545	n.s.	0.404	n.s.	n.s.
*Haemophilus sputorum*		1.941	n.s.	0.491	n.s.	0.932
*Kingella denitrificans*		3.762	n.s.	1.959	2.629	2.87
*Kingella oralis*		5.87	n.s.	5.085	n.s.	n.s.
*Lachnoanaerobaculum orale*		2.861	n.s.	1.711	n.s.	n.s.
*Lachnoanaerobaculum saburreum*		4.332	n.s.	2.821	n.s.	3.434
*Lautropia mirabilis*		5.487	n.s.	2.692	4.081	3.931
*Leptotrichia hofstadii*		4.045	4.871	2.182	2.558	2.654
*Leptotrichia massiliensis*		3.965	n.s.	1.929	1.941	2.094
*Leptotrichia trevisanii*		3.341	n.s.	1.968	1.966	1.871
*Leptotrichia wadei*		4.229	n.s.	2.619	2.673	2.909
*Mogibacterium divearsum*		1.633	n.s.	0.608	n.s.	n.s.
*Morococcus cerebrosus*		3.555	n.s.	1.868	2.431	2.284
*Neisseria bacilliformis*		3.938	n.s.	2.486	n.s.	3.134
*Neisseria bergeri*		2.141	n.s.	0.514	0.787	0.728
*Neisseria cinerea*		2.054	3.24	1.015	1.239	0.69
*Neisseria elongata*		4.677	n.s.	3.304	4.05	n.s.
*Neisseria flavescens*		3.351	4.428	1.384	1.704	1.966
*Neisseria gonorrhoeae*		1.612	2.564	0.281	0.539	0.527
*Neisseria lactamica*		2.242	n.s.	0.947	1.406	n.s.
*Neisseria macacae*		3.691	n.s.	1.935	2.588	2.689
*Neisseria meningitidis*		3.595	4.558	1.816	1.954	2.3
*Neisseria mucosa*		3.213	4.471	1.723	2.172	2.257
*Neisseria polysaccharea*		2.026	n.s.	0.73	0.977	0.595
*Neisseria sicca*		3.901	n.s.	2.175	2.614	2.693
*Neisseria subflava*		2.203	3.275	0.5	1.109	0.944

*Oribacterium sinus*	Commensal	2.064	n.s.	1.062	n.s.	n.s.
*Peptoniphilus lacrimalis*		3.832	n.s.	3.382	n.s.	n.s.
Porphyromonas catoniae		3.73	*n*.s.	2.608	*n*.s.	2.579
*Prevotella nanceiensis*		2.395	n.s.	0.905	n.s.	1.372
*Prevotella oulorum*		3.439	n.s.	2.536	2.267	n.s.
*Prevotella salivae*		2.415	n.s.	1.271	n.s.	n.s.
*Prevotella scopos*		2.042	n.s.	0.848	n.s.	1.035
*Rothia aeria*		5.895	n.s.	4.872	5.537	5.445
*Schaalia meyeri*		1.599	n.s.	0.791	n.s.	n.s.
*Selenomonas artemidis*		4.56	n.s.	2.785	n.s.	n.s.
*Selenomonas flueggei*		3.039	n.s.	1.891	n.s.	1.873
*Selenomonas massiliensis*		2.075	n.s.	n.s.	1.133	1.231
*Streptococcus agalactiae*		2.572	n.s.	1.405	1.331	n.s.
*Streptococcus australis*		3.045	n.s.	2.333	n.s.	n.s.
*Streptococcus chosunense*		3.121	3.945	n.s.	n.s.	n.s.
*Streptococcus cristatus*		5.57	n.s.	3.708	4.892	4.851
*Streptococcus gordonii*		5.106	n.s.	4.417	n.s.	n.s.
*Streptococcus gwangjuense*		3.23	n.s.	2.996	n.s.	n.s.
*Streptococcus infantis*		4.212	n.s.	3.548	n.s.	n.s.
*Streptococcus intermedius*		3.808	n.s.	n.s.	3.235	n.s.
*Streptococcus koreensis*		1.782	2.697	n.s.	n.s.	n.s.
*Streptococcus mitis*		5.035	n.s.	4.449	n.s.	n.s.
*Streptococcus pneumoniae*		3.926	n.s.	3.321	n.s.	n.s.
*Streptococcus pseudopneumoniae*		2.675	n.s.	2.249	n.s.	n.s.
*Streptococcus rubneri*		1.936	2.908	n.s.	n.s.	n.s.
*Streptococcus sanguinis*		5.882	n.s.	5.107	5.615	n.s.
*Streptococcus sinensis*		3.794	n.s.	1.923	3.064	3.159
*Streptococcus symci*		3.347	n.s.	2.713	n.s.	n.s.
*Streptococcus xiaochunlingii*		2.896	n.s.	1.845	n.s.	n.s.
*Veillonella atypica*		2.975	n.s.	1.779	1.711	n.s.
*Veillonella infantium*		3.558	n.s.	2.504	n.s.	n.s.
*Veillonella parvula*		6.132	n.s.	5.413	n.s.	n.s.

In contrast, however, the mouthrinse containing BA, BZ, and BFZ groups had significant reductions in Shannon-Weaver Diversity, observed species richness, and total bacteria compared to the B or BF groups (Figs. 2 and 3, BA, BZ, BFZ). Complete eradication of the supragingival plaque microbiota was not observed, but the results showed attenuated α-diversity and bacterial abundances consistent with microbial ecology curtailed of biomass accumulation. Amongst the mouthrinsing groups, impact assessment against clinically relevant groups of bacteria revealed that the BA group had greater bacterial reductions than the BZ and BFZ groups, likely arising from differences in formulations (Fig. 4). Comparisons versus the BF group showed that, after 4 and 12 weeks, BA significantly reduced bacterial abundances by 82.0% and 75.4% for commensal species, 93.6% and 91.3% for gingivitis species, and 88.5% and 85.2% for malodor species, respectively. BZ, on the other hand, significantly reduced bacterial abundances by 58.2% and 46.6% for commensal species, 85.8% and 80.2% for gingivitis species, and 68.5% after 4 weeks for malodor species after 4 and 12 weeks, respectively. While there were no statistically significant differences between the BZ and BFZ groups, comparisons versus the BF group showed that BFZ significantly reduced bacterial abundances for commensal species by 52.6% after 4 weeks; 84.5% and 75.9% for gingivitis species after 4 weeks and 12 weeks, respectively; and 60.7% for malodor species after 4 weeks. A detailed list of the individual bacterial species significantly impacted by the oral care regimen is presented in Table 4. No effects were observed against acidogenic bacteria (Fig. 4d), which were poorly represented in the collected specimens (Table 4, acidogenic species), likely owing to the trial exclusion of subjects with active caries or significant carious lesions. The clinical endpoint measures for plaque showed statistically significant reductions for the mouthrinse containing BA, BZ, BFZ groups after 4 weeks and 12 weeks when compared to B using interproximal mean and whole-mouth mean TPI scores (*p <* 0.001) with BA showing the largest degree of reduction while BZ and BFZ showed similar reductions [22].

### Enumeration of viable bacteria on supragingival and subgingival plaque

**Fig. 5:**
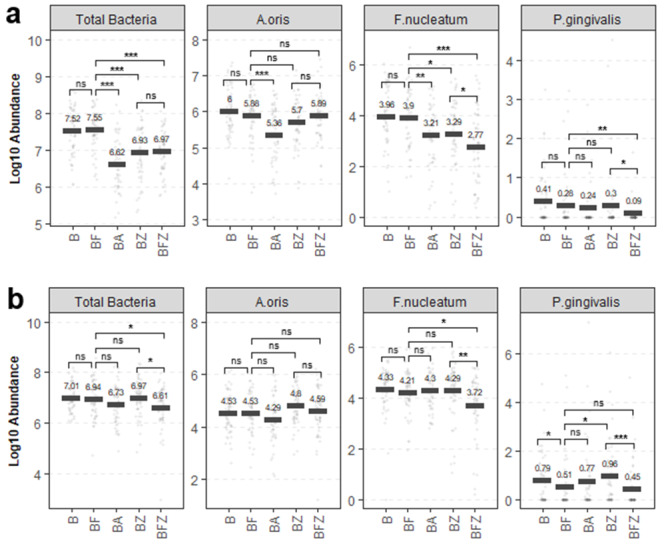
Viability qPCR results demonstrating the impact of the oral care regimen on total oral bacteria and select indicator species. The means of log10 abundance from (**a**) supragingival plaque and (**b**) subgingival plaque are shown. The dots represent individual samples. ns = not significant, *p < 0.05, **p < 0.01, ***p < 0.001

Live bacteria that remained in the plaque were quantified using vPCR targeting total bacteria and three indicator species for precise comparisons of the oral care regimen after 12 weeks (Fig. 5). While very low abundances of live *P. gingivalis* were detected throughout, the results showed marked differences in antimicrobial control based on the plaque location and mechanical and chemotherapeutic actions of the oral care regimen. In supragingival plaque, BF had no effects, while BA, BZ, and BFZ significantly reduced total bacteria and indicator species similarly to metagenome sequencing results (Fig. 5a; Table 4). A synergistic effect of combining flossing and rinsing (BFZ) was observed against *F. nucleatum* and *P. gingivalis* (Fig. 5a, BFZ). In subgingival plaque, flossing (BF) and mouthrinsing (BA, BZ) by themselves generally had no effects against total bacteria and indicator species, except for flossing (BF) against *P. gingivalis* (Fig. 5b). However, synergy was observed for the combined flossing and rinsing regimen (BFZ) against total bacteria, *F. nucleatum*, and *P. gingivalis* (Fig. 5b, BFZ). While the supragingival vPCR results provided support for the quantitative microbiome analysis, the subgingival vPCR results also showed the same trend in clinical endpoint measures for the whole-mouth mean and interproximal mean EBI and MGI scores [22]. The clinical scores showed BF and mouthrinse containing BA, BZ and BFZ groups significantly reduced bleeding and inflammation after 4 weeks (*p <* 0.001) and 12 weeks (*p* < 0.001) with BFZ showing the largest degree of reduction reflecting the synergistic antimicrobial effect against *F. nucleatum* and *P. gingivalis* subgingivally.

## Discussion

This 12-week clinical trial investigated the effects of brushing with a sodium monofluorophosphate toothpaste, plus virtually supervised flossing, and/or using EO-containing mouthrinse regimens [22] on the microbiota of supragingival and subgingival plaque. While clinical reports of superior plaque control by mouthrinses compared to flossing are on the rise [6, 7, 36–39], there is paucity of information on how plaque biofilms are affected by mechanical and chemotherapeutic means of intervention, including how constituent bacterial species and their microbial ecology respond over time.

In this trial, subjects with mild gingivitis used specific oral care regimens for 4 weeks and 12 weeks and returned to the clinic for oral and microbiome evaluations 8–18 h after the last intervention. Subjects in good periodontal and general health were also included at the baseline visit in an observational capacity to determine if different signatures of supragingival plaque microbiome exist compared to the mild gingivitis cohort. While large differences were noted in the whole-mouth and interproximal mean clinical scores for MGI and EBI, TPI showed the least amount of differentiation (Table 1) between these cohorts at recruitment and no significant high-level differences were noted in their microbiome compositions using the α- and β- diversity results (Fig. 2b, c and 3 baseline visit). Total bacterial abundance results, however, showed the mild gingivitis subjects significantly had 44% higher overall abundance compared to the healthy cohort (Fig. 2A baseline, Δ = 0.25, *p* = 0.012) with detailed low-level comparisons showing the presence of 36 species that were more abundant in gingivitis subjects (Table 3). There were no clearly differentiated microbial clusters of health vs. disease recognizable of Socransky’s subgingival plaque microbial complexes [17] or Kolenbrander’s coaggregation-based ecological succession [40] observed in this study population. However, these results demonstrate the importance of biomass accumulation in mild gingivitis subjects which is seldom investigated using relative abundance analysis offered by conventional next-generation DNA sequencing-based approach and point to the presence of different grades of periodontally healthy and early gingivitis states that show large degree of similarity in qualitative microbial diversity assessments.

The plaque microbiota represented in this mild gingivitis population exhibited both long-term accumulated product intervention effects and a short period of bacterial regrowth and recolonization. The quantitative results of supragingival plaque confirmed that daily brushing and flossing alone were insufficient to effectively manage plaque above the gingival margin (Figs. 2, 3 and 5a and a). These supragingival plaque microbiome results closely mirrored the clinical endpoint measures of interproximal mean and whole-mouth mean TPI scores [22]. Notably, the mechanical removal of supragingival plaque by brushing or flossing is likely unable to achieve sustained plaque reductions due to the rapid recolonization of plaque bacteria [41] seeded from unaffected areas of the mouth. The results of the current trial, which showed a lack of significant differences in the microbiome diversity, species richness, and total and individual bacterial abundances between brushing only and brushing and flossing regimens, support this hypothesis (B vs. BF in Figs. 2, 3 and 5a and a; Table 4).

Alcohol and non-alcohol EO-containing mouthrinses demonstrated effective and sustained chemotherapeutic means of managing supragingival plaque by maintaining reduced levels of microbiome diversity and bacterial abundances (BA, BZ, BFZ in Figs. 2, 3 and 5a and a; Table 4). This result is consistent with historically published randomized controlled trials with clinical endpoints of plaque and gingivitis efficacy [4, 5, 38, 42]. Given the results observed in this trial and the evidence base in the literature to date, we propose the following hypothesis regarding a sequence of three distinct mechanistic actions taking place against the supragingival plaque microbiome. First, 99.9% of plaque bacteria are killed within 30 s of contact [43–45], as EOs are able to penetrate thick layers of biofilms [46]. This bactericidal effect, however, is not permanent, since no complete eradication of plaque microbiota is achieved, consistent with total bacteria abundance results from the present trial and the published body of bacterial colony counting data from past clinical studies [5, 11, 13, 38]. Second, given the different antimicrobial properties of EOs compared to cationic antimicrobials that have substantivity, such as chlorhexidine gluconate or cetylpyridinium chloride [47–50], there is an attenuated level of bacterial re-seeding taking place from other areas of the mouth that facilitates plaque recolonization within a few hours. This nascent plaque is enriched with commensal bacteria, while pathogenic species associated with gingivitis or malodor are impeded due to their slow growth rates [51, 52]. The late-colonizing pathogenic species have specific requirements for metabolic and structural support from secondary and tertiary coaggregating partner species during dental plaque biofilm development [17, 53–55]. Our study results corroborate a large presence of commensal bacteria compared to gingivitis or malodor associated bacteria after mouthrinsing regimens (Fig. 4, cca. 0.3–1.1 × 10^8^ commensal versus cca. 1.5–3.8 × 10^6^ for gingivitis or malodor associated bacteria). Third, repeated twice-daily usage of EO mouthrinses helps to continually curtail plaque build-up, which prevents the maturation of biofilm and proliferation of pathogenic species associated with gingivitis and malodor, and lowers the total bacterial bioburden contributing to the maintenance of a health-associated stable oral microbial community or eubiosis (Table 4; Fig. 2a).

The analysis of subgingival plaque in this study indicated a potentially important contribution of mechanical flossing in oral health maintenance. Viable bacteria enumeration by vPCR showed that flossing can act synergistically with mouthrinsing to reduce total bacteria and *F. nucleatum* below the gingival margin (Fig. 5b, BFZ) and can selectively exert significant control against *P. gingivalis* (Fig. 5b, BF). Interestingly, these subgingival plaque vPCR results were also observed in the clinical endpoint measures of bleeding and inflammation as assessed using the interproximal and whole-mouth mean EBI and MGI scores [22] which provides support for the importance of mechanical flossing controlling subgingival plaque in synergy with mouthrinsing. This finding also supports other previous studies that demonstrated clinical improvements in gingival inflammation and bleeding scores despite poor plaque reduction by flossing [6, 7, 36–39] and sheds light on how specific oral care regimens differentially affect distinct communities of the oral microbiome. Further quantitative research is required to understand the ability of different oral care regimens and products to reach not only subgingival plaque but also other oral surfaces, such as the gingiva, cheeks, tongue, oropharynx, and saliva. In addition, immunological evaluation of pro- and anti-inflammatory cytokines with respect to the microbial community clusters that exist during the progression of different gradation of periodontal health and disease are important considerations for future studies to better understand the dynamic nature of microbial recolonization. Such a detailed assessment of microbial ecology is of significant interest for public health, as many oral bacterial species are implicated in various systemic health or disease conditions.

## Conclusions

The results of this 12-week randomized clinical trial provide numerical details of how mechanical and chemotherapeutic oral care regimens affect supragingival and subgingival microbiota. Brushing with a sodium monofluorophosphate toothpaste and flossing with a non-antimicrobial waxed dental floss alone do not appear to provide adequate control of plaque above and below the gingival margin, as constituent bacteria were unaffected, and there were no significant differences in bacterial abundances compared to the brushing control (Figs. 2, 4 and 5; Table 4, BF vs. B). However, alcohol and non-alcohol EO mouthrinses effectively managed supragingival plaque via a quick chemotherapeutic bactericidal mechanism of action, which appeared to be short-termed and allowed attenuated plaque regrowth enriched with commensal species (Figures, 2, 4, 5a, Table 4, BA, BZ). Furthermore, analysis of subgingival plaque when flossing is used in combination with mouthrinsing seemed to implicate a role for mechanical flossing in enabling the antimicrobial effectiveness of EO mouthrinses below the gingival margin (Fig. 5b, BFZ). In conclusion, this trial highlights the superior efficacy of EO mouthrinses at controlling plaque without adversely affecting its microbial ecology and elevates the role of alcohol and non-alcohol EO-containing mouthrinses beyond flossing, in conjunction with toothbrushing.

### Electronic supplementary material

Below is the link to the electronic supplementary material.

Supplementary Material 1

## Data Availability

Shotgun metagenomic sequence data and sample metadata information are available in the NCBI BioProject database under accession number PRJNA984617.

## Abbreviations

B: Brushing only
BF: Brushing and flossing
BA: Brushing and rinsing with Listerine® Cool Mint® Antiseptic
BFZ: Brushing, flossing, and rinsing with Listerine® Cool Mint® Zero Alcohol
BZ: Brushing and rinsing with Listerine® Cool Mint® Zero Alcohol
EBI: Expanded bleeding index
EO: Essential oil
MGI: Modified gingival index
NS: Not significant
PCoA: Principal coordinate analysis
PMA: Propidium monoazide
PPD: Periodontal pocket depth
TPI: Turesky Plaque Index
vPCR: Viability polymerase chain reaction
