# Supplementary material for: Quantitative analysis of the effects of brushing, flossing, and mouthrinsing on supragingival and subgingival plaque microbiota: 12-week clinical trial
Source: BMC Oral Health. 2024 May 17;24:575. doi: 10.1186/s12903-024-04362-y (PMC11102210; doi:10.1186/s12903-024-04362-y)
Supplement: Supplementary file 1 — Supplementary Material 1 [file 12903_2024_4362_MOESM1_ESM.docx]

**List of bacterial species mapped to clinical relevance**

| **Name** | **TaxID** | **Genome** | **Cavities** | **Gingivitis** | **Periodontitis** | **Malodor** | **Commensal** | **Pathogenic** | **Type** | **Heathy** | **Unknown** | **Contaminant** | **PMID** | **PMC** |
| --- | --- | --- | --- | --- | --- | --- | --- | --- | --- | --- | --- | --- | --- | --- |
| [Eubacterium] brachy | 35517 | 1.5445 | No | Yes | Yes | No | No | Yes | Opportunistic | No | No | No | 3475328, 3624445 | 269265 |
| [Eubacterium] infirmum | 56774 | 1.9090 | No | No | No | No | Yes | No |  | No | No | No | 8863423 |  |
| [Eubacterium] nodatum | 35518 | 1.8296 | No | Yes | Yes | Yes | No | Yes | Opportunistic | No | No | No | 3475328, 3624445, 1299802 | 269265 |
| [Eubacterium] saphenum | 51123 | 1.9514 | No | Yes | Yes | Yes | No | No |  | No | No | No | 26416306, 30909081, 26511188 | 2863426 |
| [Eubacterium] sulci | 143393 | 1.7340 |  | Yes | Yes | Yes |  |  |  |  | No | No |  |  |
| [Eubacterium] yurii | 39498 | 2.5325 |  |  | Yes | Yes |  |  |  |  | No | No |  |  |
| [Hallella] seregens | 52229 | 3.2668 |  | Yes | Yes |  |  |  |  |  | No | No |  |  |
| Abiotrophia defectiva | 46125 | 2.0434 |  |  |  |  | Yes |  |  |  | No | No |  |  |
| Acidipropionibacterium acidipropionici | 1748 | 3.6387 | Yes |  |  |  |  |  |  |  | No | No |  |  |
| Actinobaculum sp. oral taxon 183 | 712888 | 2.7690 |  |  | Yes |  |  |  |  |  | No | No |  |  |
| Actinomyces cardiffensis | 181487 | 2.2227 |  |  |  |  |  | Yes |  |  | No | No |  |  |
| Actinomyces dentalis | 272548 | 3.5314 |  | Yes | Yes |  |  |  |  |  | No | No |  |  |
| Actinomyces georgiae | 52768 | 2.4980 |  |  |  |  | Yes |  |  |  | No | No |  |  |
| Actinomyces gerencseriae | 52769 | 3.4200 |  |  |  |  | Yes |  |  |  | No | No | 9495607 |  |
| Actinomyces graevenitzii | 55565 | 2.1079 |  |  |  |  | Yes |  |  |  | No | No |  |  |
| Actinomyces israelii | 1659 | 3.2994 |  | Yes |  |  |  | Yes |  |  | No | No | 9495607 |  |
| Actinomyces johnsonii | 544581 | 3.3527 |  |  |  |  | Yes |  |  |  | No | No |  |  |
| Actinomyces marseillensis | 1852376 | 2.0076 |  |  |  |  | Yes |  |  |  | No | No | 29556407 | 5857164 |
| Actinomyces massiliensis | 461393 | 3.3937 |  |  |  |  | Yes |  |  |  | No | No |  |  |
| Actinomyces naeslundii | 1655 | 3.1478 |  |  |  |  | Yes |  |  |  | No | No | 9495607 |  |
| Actinomyces odontolyticus | 1660 | 2.3940 |  |  |  | Yes | Yes |  |  |  | No | No | 9495607, 16091443 |  |
| Actinomyces oricola | 206043 | 2.9307 |  |  |  |  | Yes | Yes |  |  | No | No | 25788515 | 4402957 |
| Actinomyces oris | 544580 | 3.1092 |  |  |  |  | Yes |  |  |  | No | No |  |  |
| Actinomyces slackii | 52774 | 3.1958 |  |  |  |  | Yes |  |  |  | No | No |  |  |
| Actinomyces timonensis | 1288391 | 2.9329 |  |  |  |  | Yes |  |  |  | No | No |  |  |
| Actinomyces viscosus | 1656 | 3.3364 |  |  |  |  | Yes |  |  |  | No | No |  |  |
| Aggregatibacter actinomycetemcomitans | 714 | 2.1276 |  |  | Yes |  |  | Yes |  |  | No | No | 25862077, 25139407 | 4495649, 4213353 |
| Aggregatibacter aphrophilus | 732 | 2.3329 |  |  |  |  | Yes |  |  |  | No | No | 9495607 |  |
| Aggregatibacter segnis | 739 | 1.9657 |  |  |  |  | Yes |  |  |  | No | No |  |  |
| Alloprevotella rava | 671218 | 2.5939 |  |  |  |  | Yes |  |  |  | No | No |  |  |
| Alloprevotella tannerae | 76122 | 2.5857 |  |  |  |  | Yes |  |  |  | No | No |  |  |
| Alloscardovia omnicolens | 419015 | 1.7924 |  |  |  |  | Yes |  |  |  | No | No |  |  |
| Anaeroglobus geminatus | 156456 | 1.7989 |  |  | Yes |  |  |  |  |  | No | No | 22170420 | 3358035 |
| Atopobium parvulum | 1382 | 1.5358 |  |  |  | Yes | Yes |  |  |  | No | No |  |  |
| Atopobium rimae | 1383 | 1.6281 |  |  |  |  | Yes |  |  |  | No | No |  |  |
| Bacteroides thetaiotaomicron | 818 | 6.3729 |  |  |  |  | Yes |  |  |  | No | No |  |  |
| Bacteroides uniformis | 820 | 4.9218 |  |  |  |  | Yes |  |  |  | No | No |  |  |
| Bacteroidetes oral taxon 274 | 652708 | 2.1135 |  |  |  |  | Yes |  |  |  | No | No |  |  |
| Bifidobacterium dentium | 1689 | 2.6255 | Yes |  |  |  |  |  |  |  | No | No |  |  |
| Bulleidia extructa | 118748 | 1.4196 |  |  |  |  |  | Yes |  |  | No | No |  |  |
| Campylobacter concisus | 199 | 1.9414 |  |  |  |  | Yes |  |  |  | No | No |  |  |
| Campylobacter curvus | 200 | 1.9713 |  |  |  |  | Yes |  |  |  | No | No |  |  |
| Campylobacter gracilis | 824 | 2.2685 |  |  |  |  | Yes |  |  |  | No | No |  |  |
| Campylobacter rectus | 203 | 2.5022 |  |  | Yes |  |  |  |  |  | No | No |  |  |
| Campylobacter showae | 204 | 2.1874 |  |  | Yes |  |  |  |  |  | No | No |  |  |
| candidate division SR1 bacterium MGEHA | 1293577 | 1.1097 |  |  |  | Yes |  |  |  |  | No | No | 23509275 | 3619370 |
| candidate division TM7 single-cell isolate TM7a | 447454 | 2.8644 |  |  |  |  | Yes | No |  |  | No | No | 30894042 | 6481004 |
| candidate division TM7 single-cell isolate TM7b | 447455 | 0.1128 |  |  |  |  | Yes | No |  |  | No | No | 30894042 | 6481004 |
| candidate division TM7 single-cell isolate TM7c | 447456 | 0.4742 |  |  |  |  | Yes | No |  |  | No | No | 30894042 | 6481004 |
| Candidatus Nanosynbacter lyticus | 2093824 | 0.7051 |  |  |  |  | Yes |  |  |  | No | No | 32839546 | 7784981 |
| Candidatus Saccharibacteria oral taxon TM7x | 1476577 | 0.7051 |  |  |  |  | Yes | No |  |  | No | No | 30894042 | 6481004 |
| Capnocytophaga endodontalis | 2708117 | 3.4127 |  |  |  |  | Yes | Yes |  |  | No | No |  |  |
| Capnocytophaga gingivalis | 1017 | 2.7529 |  | Maybe |  |  | Yes | Yes | Opportunistic | No | No | No | 518239 |  |
| Capnocytophaga granulosa | 45242 | 2.7463 |  |  |  |  | Yes | Yes | Opportunistic | No | No | No |  |  |
| Capnocytophaga ochracea | 1018 | 2.6587 |  |  |  |  | Yes |  |  |  | No | No | 9495607 |  |
| Capnocytophaga sputigena | 1019 | 2.9978 |  | Maybe |  |  | Yes |  |  |  | No | No | 9495607, 21895662 | 3253172 |
| Cardiobacterium hominis | 2718 | 2.6038 |  |  | Yes |  |  | Yes | Opportunistic | No | No | No | 25862077, 25139407 | 4495649, 4213353 |
| Cardiobacterium valvarum | 194702 | 2.5535 |  | Yes |  |  |  | Yes | Opportunistic | No | No | No | 31275528 | 6598494 |
| Catonella morbi | 43997 | 3.4792 | Yes |  |  |  |  | Yes | Opportunistic | No | No | No | 28413200, 27087538 |  |
| Centipeda periodontii | 82203 | 2.7613 |  |  | Yes | Yes |  |  |  |  | No | No | 25037463, 2639302 |  |
| Chloroflexi bacterium oral taxon 439 | 712934 | 1.1600 |  |  |  |  | Yes | No |  | No | No | No | 26764907 | 4713201 |
| Corynebacterium durum | 61592 | 3.4016 | No | No | No | No | Yes | No |  | No | No | No | 26811460, 32020052 | 4760785, 7174362 |
| Corynebacterium matruchotii | 43768 | 2.8560 | No | No | No | No | Yes | No |  | No | No | No | 264880 |  |
| Cryptobacterium curtum | 84163 | 1.6178 |  | Yes | Yes | Yes | No | Yes |  | No | No | No | 28979069, 10425779, 17064396 | 5621177, |
| Cutibacterium acnes | 1747 | 2.5024 |  |  |  |  | Yes | Yes | Opportunistic | No | No | No | 26856712 | 5069318 |
| Desulfobulbus sp. oral taxon 041 | 712258 | 2.7042 |  |  |  |  | Yes |  |  |  | No | No | 22170420, 23555659 | 3358035, 3608642 |
| Dialister invisus | 218538 | 1.8960 | Yes | Yes | Yes | Yes | No | Yes | Opportunistic | No | No | No | 14657126, 27219464 | 5122475 |
| Dialister micraerophilus | 309120 | 1.3388 |  |  |  |  | Yes | Yes | Opportunistic |  | No | No | 16280512 |  |
| Dialister pneumosintes | 39950 | 1.2607 |  | Yes | Yes |  | No | Yes |  |  | No | No |  |  |
| Eggerthia catenaformis | 31973 | 1.9452 | Yes | Yes | Yes |  | No | Yes | Opportunistic | No | No | No | 26172397, 31002871 |  |
| Eikenella corrodens | 539 | 2.2431 |  | Yes |  |  |  | Yes | Opportunistic |  | No | No | 9495607 |  |
| Enterobacter quasihormaechei | 2529382 | 4.5560 |  |  |  |  | Yes |  |  |  | No | No |  |  |
| Enterobacter wuhouensis | 2529381 | 4.8974 |  |  |  |  | Yes |  |  |  | No | No |  |  |
| Filifactor alocis | 143361 | 1.9310 | No | No | Yes | No | No | Yes | Infectious | No | No | No | 22170420, 25841800 | 3358035, 4485945 |
| Fretibacterium fastidiosum | 651822 | 2.7283 |  |  | Yes |  | No |  |  | No | No | No | 22493171, 26936213 | 42924544 |
| Fusobacterium hwasookii | 1583098 | 2.4358 |  |  | Yes |  | No |  |  | No | No | No | 25257648 |  |
| Fusobacterium necrophorum | 859 | 2.2018 |  |  |  |  | Yes | Yes |  |  | No | No |  |  |
| Fusobacterium nucleatum | 851 | 2.3924 |  | Yes | Yes | Yes | No | Yes | Opportunistic | No | No | No |  |  |
| Fusobacterium periodonticum | 860 | 2.5463 |  |  |  | Yes |  |  |  |  | No | No | 22355729 | 3253589 |
| Gemella haemolysans | 1379 | 1.9162 |  | Yes |  |  | No | Yes | Opportunistic | No | No | No | 9574693, 22837552 | 104816, 3420397 |
| Gemella morbillorum | 29391 | 1.7569 |  |  |  |  | Yes | Yes | Opportunistic | No | No | No | 22170420, 9495607 | 3358035 |
| Gemella sanguinis | 84135 | 1.7966 |  |  |  |  | Yes | Yes | Opportunistic | No | No | No | 23520516 | 3592792 |
| Granulicatella adiacens | 46124 | 1.9472 |  |  |  |  | Yes | Yes | Opportunistic |  | No | No | 11154413, 10405400, 29716699, 25139407 | 85281, 5993913, 4213353 |
| Granulicatella elegans | 137732 | 1.7434 |  |  |  |  | Yes | Yes | Opportunistic |  | No | No | 11154413, 10405400 | 85281 |
| Haemophilus haemolyticus | 726 | 1.9151 |  |  |  |  | Yes | No |  | No | No | No | 17687018 | 2045313 |
| Haemophilus influenzae | 727 | 1.8477 |  |  |  |  | Yes | Yes | Opportunistic | No | No | No | 17687018 | 2045313 |
| Haemophilus parahaemolyticus | 735 | 2.0940 |  |  |  |  | Yes | Yes | Opportunistic | No | No | No | 24696434 | 3993099 |
| Haemophilus parainfluenzae | 729 | 2.0792 |  |  |  |  | Yes | Yes | Opportunistic | No | No | No | 6500711 | 261613 |
| Haemophilus paraphrohaemolyticus | 736 | 2.0212 |  |  |  |  | Yes | Yes | Opportunistic | No | No | No | 24696434 | 3993099 |
| Haemophilus pittmaniae | 249188 | 2.1826 |  |  |  |  | Yes | Yes | Opportunistic | No | No | No | 24696434 | 3993099 |
| Haemophilus sputorum | 1078480 | 2.0801 |  |  |  |  | Yes | Yes | Opportunistic | No | No | No | 22336150 |  |
| Johnsonella ignava | 43995 | 2.6871 |  |  |  |  | Yes | Yes | Opportunistic | No | No | No | 22817758, 30642137 | 3507910, 6352272 |
| Kingella denitrificans | 502 | 2.2205 |  |  |  |  | Yes | Yes | Opportunistic |  | No | No | 25821962 | 4378984 |
| Kingella oralis | 505 | 2.4067 |  |  |  |  | Yes | No |  | No | No | No | 9467377 |  |
| Lachnoanaerobaculum gingivalis | 2490855 | 3.0980 |  | Yes |  |  | No |  |  |  | No | No |  |  |
| Lachnoanaerobaculum orale | 979627 | 2.8010 |  |  |  |  | Yes |  |  |  | No | No | 22228654 | 3541798 |
| Lachnoanaerobaculum saburreum | 467210 | 3.0741 |  |  |  |  | Yes | No |  | No | No | No | 22228654 | 3541795 |
| Lactobacillus casei | 1582 | 2.9894 | Yes |  |  |  | Yes | No |  | Yes | No | No | 25758458 | 4547204 |
| Lactobacillus fermentum | 1613 | 2.0118 | Yes | No | No |  | Yes | Yes | Opportunistic | Yes | No | No | 25758458 | 4547204 |
| Lactobacillus gasseri | 1596 | 1.9173 | Yes | No | No |  | Yes | Yes | Opportunistic | Yes | No | No | 25758458 | 4547204 |
| Lactobacillus oris | 1632 | 2.1160 | No | No | No |  | Yes | No |  | Yes | No | No | 25758458 | 4547204 |
| Lactobacillus paracasei | 1597 | 3.0001 | No | No | No |  | Yes | No |  | Yes | No | No | 20502929 | 3133768 |
| Lactobacillus rhamnosus | 47715 | 2.9490 | Yes | No | No |  | Yes | Yes | Opportunistic | Yes | No | No | 25758458 | 4547204 |
| Lactobacillus salivarius | 1624 | 1.9836 | Yes |  |  |  | Yes | No |  | Yes | No | No | 25758458 | 4547204 |
| Lactobacillus vaginalis | 1633 | 1.8786 |  |  |  |  | Yes | No |  |  | No | No | 25758458 | 4547204 |
| Lautropia dentalis | 2490857 | 3.8272 |  | Yes |  |  | No |  |  |  | No | No |  |  |
| Lautropia mirabilis | 47671 | 3.1620 |  |  |  |  | Yes | No |  |  | No | No | 8075812 |  |
| Leptotrichia buccalis | 40542 | 2.4656 |  | Yes |  |  |  | Yes | Opportunistic | No | No | No | 29666288 | 5904416 |
| Leptotrichia goodfellowii | 157692 | 2.2873 |  | Yes |  |  | Yes | Yes | Opportunistic | No | No | No | 29081911 | 5646626 |
| Leptotrichia hofstadii | 157688 | 2.5607 |  |  |  |  | Yes | No |  | No | No | No | 15023979 |  |
| Leptotrichia hongkongensis | 554406 | 2.2842 |  | Yes |  |  | No | Yes | Opportunistic |  | No | No |  |  |
| Leptotrichia massiliensis | 1852388 | 2.5386 |  |  |  |  | Yes |  |  |  | No | No | 29556407 | 5857164 |
| Leptotrichia shahii | 157691 | 2.1529 |  | Yes |  |  |  | No |  |  | No | No | 15023979 |  |
| Leptotrichia trevisanii | 109328 | 2.8534 |  |  |  |  | Yes | Yes | Opportunistic |  | No | No | 30547754 | 6295021 |
| Leptotrichia wadei | 157687 | 2.3694 |  |  |  |  | Yes | Yes | Opportunistic |  | No | No | 15023979 |  |
| Megasphaera micronuciformis | 187326 | 1.7655 |  |  | Yes | Yes |  | No |  |  | No | No | 22355729, 17021095 | 3253589, 1594761 |
| Methanobrevibacter oralis | 66851 | 2.1162 |  | Yes | Yes | Yes |  |  |  |  | No | No | 24558239 | 3931360 |
| Mitsuokella sp. oral taxon 131 | 1321780 | 2.2285 |  |  | Yes |  |  | Yes | Infectious | No | No | No | 7478766 |  |
| Mogibacterium diversum | 114527 | 1.4827 |  |  |  |  | Yes |  |  |  | No | No | 11837293 |  |
| Mogibacterium sp. CM50 | 936375 | 1.8885 |  | Yes | Yes |  |  | Yes | Infectious | No | No | No | 22057871, 29339824 | 3255620, 5932080 |
| Mogibacterium timidum | 35519 | 1.8062 |  | Yes | Yes |  |  | Yes | Infectious | No | No | No | 24031909 | 3768883 |
| Morococcus cerebrosus | 1056807 | 2.4482 |  |  |  |  | Yes | Yes | Opportunistic | No | No | No | 29378989 |  |
| Mycoplasma orale | 2121 | 0.7584 |  |  |  |  | Yes | Yes | Opportunistic |  | No | No |  |  |
| Mycoplasma salivarium | 2124 | 1.2254 |  | Yes |  |  |  | Yes | Opportunistic | No | No | No | 4254176, 3711294 | 268787 |
| Neisseria bacilliformis | 267212 | 2.4000 |  |  |  |  | Yes | Yes | Opportunistic | No | No | No | 16455901 | 1392657 |
| Neisseria bergeri | 1906581 | 2.1293 |  |  |  |  | Yes |  |  |  | No | No |  |  |
| Neisseria cinerea | 483 | 1.8745 |  |  |  |  | Yes | Yes | Opportunistic | No | No | No | 6361062 | 270980 |
| Neisseria elongata | 495 | 2.3979 |  |  |  |  | Yes | Yes | Opportunistic | No | No | No | 25814039, 5488467 |  |
| Neisseria flavescens | 484 | 2.2614 |  |  |  | Yes | Yes | Yes | Opportunistic | No | No | No | 22355729 | 3253589 |
| Neisseria gonorrhoeae | 485 | 2.1450 |  |  |  |  | Yes | Yes | Opportunistic | No | No | No | 28369241, 24562188 | 3969750 |
| Neisseria lactamica | 486 | 2.1855 |  |  |  |  | Yes | Yes | Opportunistic | No | No | No | 31959912, 27572971 | 6971049 |
| Neisseria macacae | 496 | 2.7484 |  |  |  |  | Yes | Yes | Opportunistic | No | No | No | 24097834 | 3799226 |
| Neisseria meningitidis | 487 | 2.1310 |  |  |  |  | Yes | Yes | Opportunistic | No | No | No | 19464092 | 2719693 |
| Neisseria mucosa | 488 | 2.5169 |  |  |  |  | Yes | Yes | Opportunistic | No | No | No | 22798652 | 3709538 |
| Neisseria polysaccharea | 489 | 2.0908 |  |  |  |  | Yes | No |  | No | No | No |  |  |
| Neisseria sicca | 490 | 2.5214 |  |  |  |  | Yes | No |  | No | No | No | 27572971, 773308 | 169827 |
| Neisseria subflava | 28449 | 2.2624 |  |  |  |  | Yes | Yes | Opportunistic | No | No | No | 25814039 |  |
| Neoactinobaculum massilliense | 2364794 | 1.8677 |  |  |  |  | Yes |  |  |  | No | No | 31737278 | 6849414 |
| Olsenella profusa | 138595 | 2.7249 | Yes | Yes | Yes |  |  | No |  | No | No | No | 11594611 |  |
| Olsenella uli | 133926 | 2.1532 |  | Yes | Yes |  |  | Yes | Infectious | No | No | No | 11594611 |  |
| Oribacterium asaccharolyticum | 1501332 | 2.5206 |  | Yes |  |  |  | No |  | No | No | No | 24824639, 29988721 | 4129163, 6032013 |
| Oribacterium parvum | 1501329 | 2.4764 |  |  |  |  | Yes | No |  | No | No | No | 24824639 | 4129163 |
| Oribacterium sinus | 237576 | 2.7070 |  |  |  |  | Yes | No |  | No | No | No | 15388717, 17021095 | 1594761 |
| Parascardovia denticolens | 78258 | 1.8915 | Yes |  |  |  |  | No |  | No | No | No | 16707878, 12054242 |  |
| Parvimonas micra | 33033 | 1.6791 |  | Yes | Yes |  |  | Yes | Infectious | No | No | No | 23574465 | 3912758 |
| Peptidiphaga gingivicola | 2741497 | 2.5248 |  |  | Yes |  | No |  |  |  | No | No | 29105370 | 5771945 |
| Peptoanaerobacter stomatis | 796937 | 2.5485 |  |  | Yes |  |  | Yes | Infectious | No | No | No | 31052371, 28438978 | 6630776, 5478963 |
| Peptoniphilus lacrimalis | 33031 | 1.6992 |  |  |  |  | Yes | Yes | Opportunitic | No | No | No | 1390111 |  |
| Peptoniphilus mikwangii | 1354300 | 1.4998 |  |  |  |  | Yes | Yes | Opportunitic | No | No | No | 25319028 |  |
| Peptostreptococcus stomatis | 341694 | 1.9880 |  | Yes | Yes |  |  | Yes | Opportunistic | No | No | No | 16585688, 22413030 | 3295795 |
| Porphyromonas catoniae | 41976 | 2.0708 |  |  |  |  | Yes | No |  | No | No | No | 15583276, 8590687 | 535285 |
| Porphyromonas endodontalis | 28124 | 2.0881 |  |  | Yes | Yes |  | Yes | Infectious | No | No | No | 25139407, 2082242 | 4213353 |
| Porphyromonas gingivalis | 837 | 2.3318 |  |  | Yes | Yes |  | Yes | Infectious | No | No | No | 22170420, 9495607, 15752104, 11083813 | 3358035, 97798 |
| Prevotella aurantiaca | 596085 | 3.0022 |  |  | Yes |  |  | No |  | No | No | No | 19654360 |  |
| Prevotella baroniae | 305719 | 3.1162 |  |  | Yes |  |  | Yes | Opportunistic | No | No | No | 16014480 |  |
| Prevotella bivia | 28125 | 2.4903 |  | Yes | Yes |  |  | Yes | Infectious | No | No | No | 19161595 | 2637877 |
| Prevotella buccae | 28126 | 3.2032 |  |  |  |  | Yes | Yes | Opportunistic | No | No | No | 22684253 |  |
| Prevotella buccalis | 28127 | 3.0340 |  |  |  |  | Yes |  |  |  | No | No |  |  |
| Prevotella dentalis | 52227 | 3.3183 |  |  |  |  | Yes | Yes | Opportunistic | No | No | No | 28979069 | 5621177 |
| Prevotella denticola | 28129 | 3.0535 |  | Yes | Yes |  | No |  |  | No | No | No | 22170420, 22684253 | 3358035 |
| Prevotella enoeca | 76123 | 2.8227 |  |  |  |  | Yes | No |  | No | No | No | 7981091 |  |
| Prevotella fusca | 589436 | 3.2178 |  |  |  |  | Yes | No |  | No | No | No | 20495041 |  |
| Prevotella histicola | 470565 | 2.9924 | Yes |  |  |  | Yes | No |  | No | No | No |  |  |
| Prevotella intermedia | 28131 | 2.7778 |  | Yes | Yes | Yes |  |  |  | No | No | No | 22170420, 22684253 | 3358035 |
| Prevotella koreensis | 2490854 | 2.6915 |  |  | Yes |  | No |  |  |  | No | No | 31214821 |  |
| Prevotella loescheii | 840 | 3.4753 |  | Yes | Yes | Yes |  | Yes | Opportunistic | No | No | No | 1390106, 2082242 |  |
| Prevotella maculosa | 439703 | 3.2964 |  |  |  |  | Yes |  |  | No | No | No | 22684253 |  |
| Prevotella marshii | 189722 | 2.5388 |  |  |  |  | Yes | Yes | Opportunistic | No | No | No | 16014480 |  |
| Prevotella melaninogenica | 28132 | 3.1682 |  |  |  | Yes | Yes |  |  | No | No | No | 22355729, 22684253 | 3253589 |
| Prevotella micans | 189723 | 2.4353 |  |  |  |  | Yes | No |  | No | No | No | 19329604 |  |
| Prevotella multiformis | 282402 | 3.0574 |  |  |  |  | Yes |  |  | No | No | No | 22684253 |  |
| Prevotella multisaccharivorax | 310514 | 3.3886 | Yes |  | Yes |  |  | No |  | No | No | No | 19801230 |  |
| Prevotella nanceiensis | 425941 | 2.6488 |  |  |  |  | Yes | Yes | Opportunistic | No | No | No | 17911286 |  |
| Prevotella nigrescens | 28133 | 2.8380 | Yes |  |  |  | Yes |  |  | No | No | No | 9495607, 22684253 |  |
| Prevotella oralis | 28134 | 2.8574 |  |  |  |  | Yes |  |  | No | No | No | 22684253 |  |
| Prevotella oris | 28135 | 3.1682 |  |  |  |  | Yes |  |  | No | No | No | 22684253 |  |
| Prevotella oulorum | 28136 | 2.8278 |  |  |  |  | Yes |  |  | No | No | No | 22684253 |  |
| Prevotella pallens | 60133 | 3.1087 |  |  |  | Yes |  |  |  | No | No | No | 22355729 | 3253589 |
| Prevotella pleuritidis | 407975 | 2.6350 |  |  |  |  | Yes |  |  | No | No | No | 22684253 |  |
| Prevotella saccharolytica | 633701 | 2.9145 |  |  |  |  | Yes | No |  | No | No | No | 19946051 | 3931283 |
| Prevotella salivae | 228604 | 3.2746 |  |  |  |  | Yes |  |  | No | No | No | 15143039 |  |
| Prevotella scopos | 589437 | 3.2099 |  |  |  |  | Yes | No |  | No | No | No | 20495041 |  |
| Prevotella shahii | 228603 | 3.5007 |  |  |  | Yes | Yes | No |  | No | No | No | 22355729 | 3253589 |
| Prevotella veroralis | 28137 | 2.8458 |  |  |  | Yes | Yes | No |  | No | No | No | 1390106 |  |
| Propionibacterium acidifaciens | 556499 | 3.0439 | Yes |  |  |  |  |  |  | No | No | No | 31254840 |  |
| Pseudopropionibacterium propionicum | 1750 | 3.4051 |  |  | Yes |  |  | Yes | Opportunistic | No | No | No | 31052361 | 6630690 |
| Pseudoramibacter alactolyticus | 113287 | 2.3629 |  |  | Yes |  |  | Yes | Opportunistic | No | No | No | 14651280 |  |
| Pyramidobacter piscolens | 638849 | 2.9312 | Yes |  | Yes |  |  | Yes | Opportunistic | No | No | No | 19406777 | 2868594 |
| Rothia aeria | 172042 | 2.5887 |  |  |  |  | Yes | Yes | Opportunistic | No | No | No | 28082174, 28082174 |  |
| Rothia dentocariosa | 2047 | 2.4928 | Yes |  |  |  | Yes | Yes | Opportunistic | No | No | No | 9495607, 3475328 |  |
| Rothia mucilaginosa | 43675 | 2.2852 | Yes |  |  |  | Yes | Yes | Opportunistic | No | No | No |  |  |
| Scardovia inopinata | 78259 | 1.8009 | Yes |  |  |  |  | Yes | Opportunistic | No | No | No | 12054242 |  |
| Scardovia wiggsiae | 230143 | 1.5532 | Yes |  |  |  |  | No |  | No | No | No | 29104444 | 5665406 |
| Schaalia meyeri | 52773 | 2.0378 |  |  |  |  | Yes | Yes | Opportunistic |  | No | No |  |  |
| Selenomonas artemidis | 671224 | 2.2805 |  |  |  | Yes | Yes | Yes | Opportunistic | No | No | No | 2405009 | 269556 |
| Selenomonas felix | 1944634 | 2.4032 |  |  |  |  | Yes |  |  |  | No | No | 31333847 | 6614701 |
| Selenomonas flueggei | 135080 | 2.1661 |  |  |  |  | Yes | No |  | No | No | No | 20831580 |  |
| Selenomonas infelix | 135082 | 2.4136 |  |  |  |  | Yes | Yes | Opportunistic | No | No | No | 2405009 | 269556 |
| Selenomonas massiliensis | 2058293 | 2.5870 |  |  |  |  | Yes |  |  |  | No | No | 29922467 | 6004730 |
| Selenomonas noxia | 135083 | 2.0842 |  |  | Yes |  |  | Yes | Infectious | No | No | No | 9495607 |  |
| Selenomonas sputigena | 69823 | 2.5639 |  |  | Yes |  |  | Yes | Infectious | No | No | No | 129552, 27563202 | 4976549 |
| Shuttleworthia satelles | 177972 | 2.1695 |  | Yes | Yes |  |  | Yes | Opportunistic | No | No | No | 12361248 |  |
| Slackia exigua | 84109 | 2.0995 |  | Yes | Yes | Yes |  | Yes | Opportunistic | No | No | No | 28979069 | 5621177 |
| Solobacterium moorei | 102148 | 1.9990 |  |  |  | Yes | Yes | Yes | Opportunistic | No | No | No | 22355729, 21525228 | 3253589, 3147872 |
| Stomatobaculum longum | 796942 | 2.3136 |  |  |  |  | Yes | Yes | Opportunistic | No | No | No | 22843721, 31874981 | 3709536, 6930300 |
| Streptobacillus hongkongensis | 1162717 | 1.4605 |  |  |  |  | Yes |  |  |  | No | No | 27074987 | 4831002 |
| Streptococcus agalactiae | 1311 | 2.0829 |  |  |  |  | Yes | Yes | Opportunistic | No | No | No | 19721085 | 2738137 |
| Streptococcus anginosus | 1328 | 1.9582 |  |  |  |  | Yes | Yes | Opportunistic | No | No | No | 10843047 |  |
| Streptococcus australis | 113107 | 1.9624 |  |  |  |  | Yes | Yes | Opportunistic | No | No | No | 11491323 |  |
| Streptococcus chosunense | 2707003 | 1.9021 |  |  |  |  | Yes | Yes | Opportunistic |  | No | No | 31332483 |  |
| Streptococcus constellatus | 76860 | 1.9139 |  | Yes | Yes |  | Yes | Yes | Opportunistic | No | No | No | 19829816 | 2740169 |
| Streptococcus cristatus | 45634 | 2.0731 |  |  |  |  | Yes | No |  | Yes | No | No | 20826648 | 3020839 |
| Streptococcus gordonii | 1302 | 2.1926 |  |  |  |  | Yes | Yes | Opportunistic | Yes | No | No | 30338752, 26875613 | 6287261 |
| Streptococcus gwangjuense | 1433513 | 1.9725 |  |  |  |  | Yes | Yes | Opportunistic |  | No | No | 31028412 |  |
| Streptococcus halitosis | 2172545 | 1.8807 |  |  |  | Yes | Yes |  |  |  | No | No | 30701262 | 6346211 |
| Streptococcus infantis | 68892 | 1.8695 |  |  |  |  | Yes | No |  | No | No | No | 9734047 |  |
| Streptococcus intermedius | 1338 | 1.9424 |  |  |  |  | Yes | Yes | Opportunistic | No | No | No | 9495607, 21738290 | 3124902 |
| Streptococcus koreensis | 2382163 | 2.0096 |  |  |  |  | Yes |  |  |  | No | No |  |  |
| Streptococcus massiliensis | 313439 | 1.8642 |  |  |  |  | Yes | No |  | No | No | No | 26782571 |  |
| Streptococcus milleri | 33040 | 1.8909 |  |  |  |  | Yes | Yes | Opportunistic |  | No | No |  |  |
| Streptococcus mitis | 28037 | 2.0027 |  |  |  |  | Yes | Yes | Opportunistic | Yes | No | No | 3475328, 30338752, 26875613 | 6287261 |
| Streptococcus mutans | 1309 | 1.9839 | Yes |  |  |  | Yes | No |  | No | No | No | 9495607, 30338752 | 6287261 |
| Streptococcus oralis | 1303 | 1.9698 |  |  |  |  | Yes | Yes | Opportunistic | Yes | No | No | 9495607, 30338752, 26875613 | 6287261 |
| Streptococcus parasanguinis | 1318 | 2.1351 |  |  |  |  | Yes | Yes | Opportunistic | Yes | No | No | 30338752, 26875613 | 6287261 |
| Streptococcus periodonticum | 2490633 | 1.8774 |  |  |  |  | Yes |  |  |  | No | No |  |  |
| Streptococcus peroris | 68891 | 1.6399 |  |  |  |  | Yes | No |  | No | No | No | 9734047 |  |
| Streptococcus pneumoniae | 1313 | 2.0860 |  |  |  |  | Yes | Yes | Opportunistic | No | No | No | 10678950 | 97291 |
| Streptococcus pseudopneumoniae | 257758 | 2.1726 |  |  |  |  | Yes | Yes | Opportunistic | No | No | No |  |  |
| Streptococcus pyogenes | 1314 | 1.7914 |  |  |  |  |  | Yes | Infectious | No | No | No | 19721085 | 2738137 |
| Streptococcus rubneri | 1234680 | 2.1601 |  |  |  |  | Yes |  |  |  | No | No | 23749274 |  |
| Streptococcus salivarius | 1304 | 2.1855 |  |  |  |  | Yes | Yes | Opportunistic | Yes | No | No | 24271166 | 3911234 |
| Streptococcus sanguinis | 1305 | 2.3622 |  |  |  |  | Yes | Yes | Opportunistic | No | No | No | 22170420, 30338752, 26875613 | 3358035, 6287261 |
| Streptococcus sinensis | 176090 | 2.0618 |  |  |  |  | Yes | Yes | Opportunistic | No | No | No | 11880397, 19330895 | 120286 |
| Streptococcus sobrinus | 1310 | 2.1125 | Yes |  |  |  | Yes | No |  | No | No | No | 30338752 | 6287261 |
| Streptococcus symci | 2588991 | 2.1894 |  |  |  |  | Yes |  |  |  | No | No | 33387140 | 7878260 |
| Streptococcus vestibularis | 1343 | 1.8980 |  |  |  | Yes | Yes | Yes | Opportunistic | No | No | No | 25983909, 16091443 | 4421209 |
| Streptococcus xiaochunlingii | 2589788 | 1.9507 |  |  |  |  | Yes |  |  |  | No | No | 33133039 | 7550633 |
| Tannerella forsythia | 28112 | 3.3002 |  |  | Yes | Yes |  | Yes | Infectious | No | No | No | 9495607, 25139407, 15752104 | 4213353 |
| TM7 phylum sp. oral taxon 346 | 713049 | 0.7205 |  |  |  |  | Yes |  |  |  | No | No |  |  |
| TM7 phylum sp. oral taxon 348 | 671231 | 0.7205 |  |  |  |  | Yes |  |  |  | No | No |  |  |
| TM7 phylum sp. oral taxon 350 | 713052 | 0.7205 |  |  |  |  | Yes |  |  |  | No | No |  |  |
| TM7 phylum sp. oral taxon 351 | 713053 | 0.7033 |  |  |  |  | Yes |  |  |  | No | No |  |  |
| TM7 phylum sp. oral taxon 352 | 713054 | 0.7033 |  |  |  |  | Yes |  |  |  | No | No |  |  |
| TM7 phylum sp. oral taxon 353 | 713055 | 0.7033 |  |  |  |  | Yes |  |  |  | No | No |  |  |
| TM7 phylum sp. oral taxon 356 | 713057 | 0.7205 |  |  |  |  | Yes |  |  |  | No | No |  |  |
| Treponema denticola | 158 | 2.8254 |  |  | Yes | Yes |  | Yes | Infectious | No | No | No | 22170420, 15752104 | 3358035 |
| Treponema lecithinolyticum | 53418 | 2.3409 |  |  | Yes |  |  | Yes | Infectious | No | No | No | 10555310, 15845368 |  |
| Treponema maltophilum | 51160 | 2.5304 |  |  | Yes |  |  | Yes | Infectious | No | No | No | 8782684 |  |
| Treponema medium | 58231 | 2.7275 |  |  | Yes |  |  | Yes | Infectious | No | No | No | 22170420, 8995804 | 3358035 |
| Treponema pectinovorum | 164 | 2.2240 |  | Yes | Yes |  |  | Yes | Infectious | No | No | No | 11411720 |  |
| Treponema putidum | 221027 | 2.7865 |  |  | Yes |  |  | Yes | Infectious | No | No | No | 16238598 |  |
| Treponema socranskii | 53419 | 2.8046 |  |  | Yes |  |  | Yes | Infectious | No | No | No | 3475328 |  |
| Treponema vincentii | 69710 | 2.6040 |  |  | Yes |  |  | Yes | Infectious | No | No | No | 22170420 | 3358035 |
| Veillonella atypica | 39777 | 2.0720 |  |  |  |  | Yes | No |  | No | No | No | 28473967 | 5397411 |
| Veillonella dispar | 39778 | 2.0843 |  |  |  | Yes | Yes | No |  | No | No | No | 28473967, 16091443 | 5397411 |
| Veillonella infantium | 1911679 | 2.0213 |  |  |  |  | Yes |  |  |  | No | No | 29458564 |  |
| Veillonella parvula | 29466 | 2.1442 |  |  |  |  | Yes | Yes | Opportunistic | No | No | No | 9495607, 28473967 | 5397411 |
| Veillonella tobetsuensis | 1110546 | 2.0986 |  |  |  |  | Yes |  |  |  | No | No | 22843723 |  |
| [Eubacterium] rectale | 39491 | 3.2345 |  |  |  |  |  |  |  |  |  | Yes | 26619944 |  |
| [Kluyvera] intestini | 1898961 | 5.7850 |  |  |  |  |  |  |  |  |  | Yes | 29074664 | 5658502 |
| [Lactobacillus] timonensis | 1970790 | 1.2807 |  |  |  |  |  |  |  |  |  | Yes | 28794887 | 5545815 |
| [Propionibacterium] humerusii | 1050843 | 2.6233 |  |  |  |  |  |  |  |  |  | Yes |  |  |
| [Propionibacterium] namnetense | 1574624 | 2.3866 |  |  |  |  |  |  |  |  |  | Yes |  |  |
| [Pseudomonas] geniculata | 86188 | 4.7140 |  |  |  |  |  |  |  |  |  | Yes |  |  |
| [Scytonema hofmanni] UTEX B 1581 | 379535 | 8.1334 |  |  |  |  |  |  |  |  |  | Yes |  |  |
| Abyssicoccus albus | 1817405 | 1.7710 |  |  |  |  |  |  |  |  |  | Yes | 27272908 |  |
| Achromobacter insuavis | 1287735 | 6.8761 |  |  |  |  |  |  |  |  |  | Yes |  |  |
| Acidaminococcus fermentans | 905 | 2.1704 |  |  |  |  |  |  |  |  |  | Yes | 21304687 | 3035267 |
| Acidovorax carolinensis | 553814 | 4.3441 |  |  |  |  |  |  |  |  |  | Yes | 29937052 | 6361392 |
| Acidovorax ebreus | 721785 | 4.2514 |  |  |  |  |  |  |  |  |  | Yes | 20023012 | 2820844 |
| Acidovorax temperans | 80878 | 4.6514 |  |  |  |  |  |  |  |  |  | Yes |  |  |
| Acinetobacter baumannii | 470 | 3.9747 |  |  |  |  |  |  |  |  |  | Yes |  |  |
| Acinetobacter bereziniae | 106648 | 4.7317 |  |  |  |  |  |  |  |  |  | Yes |  |  |
| Acinetobacter calcoaceticus/baumannii complex | 909768 | 3.6702 |  |  |  |  |  |  |  |  |  | Yes |  |  |
| Acinetobacter gyllenbergii | 134534 | 4.3059 |  |  |  |  |  |  |  |  |  | Yes |  |  |
| Acinetobacter idrijaensis | 1507807 | 2.2351 |  |  |  |  |  |  |  |  |  | Yes | 25395645 | 4241671 |
| Acinetobacter indicus | 756892 | 3.0337 |  |  |  |  |  |  |  |  |  | Yes | 22247213 |  |
| Acinetobacter johnsonii | 40214 | 3.5622 |  |  |  |  |  |  |  |  |  | Yes |  |  |
| Acinetobacter junii | 40215 | 3.3703 |  |  |  |  |  |  |  |  |  | Yes |  |  |
| Acinetobacter lwoffii | 28090 | 3.3531 |  |  |  |  |  |  |  |  |  | Yes |  |  |
| Acinetobacter marinus | 281375 | 2.9948 |  |  |  |  |  |  |  |  |  | Yes |  |  |
| Acinetobacter pittii | 48296 | 3.9936 |  |  |  |  |  |  |  |  |  | Yes |  |  |
| Acinetobacter proteolyticus | 1776741 | 4.3288 |  |  |  |  |  |  |  |  |  | Yes |  |  |
| Acinetobacter soli | 487316 | 3.4710 |  |  |  |  |  |  |  |  |  | Yes |  |  |
| Acinetobacter ursingii | 108980 | 3.4812 |  |  |  |  |  |  |  |  |  | Yes |  |  |
| Actinobacillus minor | 51047 | 2.2762 |  |  |  |  |  |  |  |  |  | Yes |  |  |
| Actinobacillus pleuropneumoniae | 715 | 2.2745 |  |  |  |  |  |  |  |  |  | Yes |  |  |
| Actinobacillus ureae | 723 | 2.4748 |  |  |  |  |  |  |  |  |  | Yes |  |  |
| Actinomyces bouchesdurhonensis | 1852361 | 2.2066 |  |  |  |  |  |  |  |  |  | Yes |  |  |
| Actinomyces bovis | 1658 | 2.5956 |  |  |  |  |  |  |  |  |  | Yes |  |  |
| Actinomyces culturomici | 1926276 | 2.5624 |  |  |  |  |  |  |  |  |  | Yes |  |  |
| Actinomyces denticolens | 52767 | 2.8551 |  |  |  |  |  |  |  |  |  | Yes |  |  |
| Actinomyces gaoshouyii | 1960083 | 2.2652 |  |  |  |  |  |  |  |  |  | Yes | 28857023 |  |
| Actinomyces howellii | 52771 | 3.1128 |  |  |  |  |  |  |  |  |  | Yes |  |  |
| Actinomyces ihuae | 1673722 | 2.4584 |  |  |  |  |  |  |  |  |  | Yes | 29992027 | 6036942 |
| Actinomyces mediterranea | 1871028 | 2.3956 |  |  |  |  |  |  |  |  |  | Yes |  |  |
| Actinomyces neuii | 33007 | 2.3076 |  |  |  |  |  | Yes |  |  |  | Yes |  |  |
| Actinomyces pacaensis | 1852377 | 1.8926 |  |  |  |  |  |  |  |  |  | Yes |  |  |
| Actinomyces polynesiensis | 1325934 | 2.8785 |  |  |  |  |  |  |  |  |  | Yes |  |  |
| Actinomyces procaprae | 2560010 | 3.6908 |  |  |  |  |  |  |  |  |  | Yes |  |  |
| Actinomyces ruminicola | 332524 | 3.0751 |  |  |  |  |  |  |  |  |  | Yes |  |  |
| Actinomyces turicensis | 131111 | 1.9888 |  |  |  |  |  |  |  |  |  | Yes |  |  |
| Actinomyces urogenitalis | 103621 | 2.6044 |  |  |  |  |  |  |  |  |  | Yes |  |  |
| Adlercreutzia caecicola | 747645 | 2.4701 |  |  |  |  |  |  |  |  |  | Yes | 34495823 | 8549271 |
| Adlercreutzia muris | 1796610 | 2.7846 |  |  |  |  |  |  |  |  |  | Yes | 27670113 |  |
| Advenella kashmirensis | 310575 | 4.5487 |  |  |  |  |  |  |  |  |  | Yes | 16166666 |  |
| Aerococcus viridans | 1377 | 2.0048 |  |  |  |  |  |  |  |  |  | Yes |  |  |
| Aeromonas caviae | 648 | 4.6330 |  |  |  |  |  |  |  |  |  | Yes | 21183677 | 3067608 |
| Aeromonas hydrophila | 644 | 4.9329 |  |  |  |  |  |  |  |  |  | Yes |  |  |
| Aeromonas rivipollensis | 948519 | 4.5752 |  |  |  |  |  |  |  |  |  | Yes | 26630331 |  |
| Afipia broomeae | 56946 | 5.2620 |  |  |  |  |  |  |  |  |  | Yes | 25874801 | 4398416 |
| Aggregatibacter kilianii | 2025884 | 2.3678 |  |  |  |  |  |  |  |  |  | Yes | 29695522 | 6018338 |
| Agrobacterium genomosp. 3 | 1183410 | 5.5293 |  |  |  |  |  |  |  |  |  | Yes |  |  |
| Agrobacterium tumefaciens | 358 | 5.6368 |  |  |  |  |  |  |  |  |  | Yes |  |  |
| Agrococcus lahaulensis | 341722 | 2.6592 |  |  |  |  |  |  |  |  |  | Yes | 16902012 |  |
| Akkermansia muciniphila | 239935 | 2.7625 |  |  |  |  |  |  |  |  |  | Yes |  |  |
| Alcaligenes faecalis | 511 | 4.1581 |  |  |  |  |  |  |  |  |  | Yes |  |  |
| Alishewanella aestuarii | 453835 | 3.5881 |  |  |  |  |  |  |  |  |  | Yes |  |  |
| Alishewanella agri | 553384 | 3.4917 |  |  |  |  |  |  |  |  |  | Yes |  |  |
| Alishewanella jeotgali | 545533 | 3.8314 |  |  |  |  |  |  |  |  |  | Yes | 19620373 |  |
| Alistipes finegoldii | 214856 | 3.5024 |  |  |  |  |  |  |  |  |  | Yes |  |  |
| Alistipes ihumii | 1470347 | 2.7728 |  |  |  |  |  |  |  |  |  | Yes |  |  |
| Alistipes indistinctus | 626932 | 2.8554 |  |  |  |  |  |  |  |  |  | Yes |  |  |
| Alistipes inops | 1501391 | 2.3048 |  |  |  |  |  |  |  |  |  | Yes |  |  |
| Alistipes obesi | 1118061 | 3.1637 |  |  |  |  |  |  |  |  |  | Yes |  |  |
| Alistipes putredinis | 28117 | 2.3723 |  |  |  |  |  |  |  |  |  | Yes |  |  |
| Alistipes senegalensis | 1288121 | 3.8938 |  |  |  |  |  |  |  |  |  | Yes |  |  |
| Alistipes shahii | 328814 | 3.3784 |  |  |  |  |  |  |  |  |  | Yes |  |  |
| Aliterella atlantica | 1827278 | 5.2657 |  |  |  |  |  |  |  |  |  | Yes | 27054834 |  |
| Alkalihalobacillus okhensis | 333138 | 4.8653 |  |  |  |  |  |  |  |  |  | Yes |  |  |
| Alloscardovia theropitheci | 2496842 | 1.9598 |  |  |  |  |  |  |  |  |  | Yes |  |  |
| Alterileibacterium massiliense | 1870997 | 1.4508 |  |  |  |  |  |  |  |  |  | Yes |  |  |
| Amycolatopsis acidicola | 2596893 | 9.4652 |  |  |  |  |  |  |  |  |  | Yes | 31851605 |  |
| Anaerococcus nagyae | 1755241 | 1.7882 |  |  |  |  |  |  |  |  |  | Yes | 26639871 |  |
| Anaerococcus octavius | 54007 | 1.9467 |  |  |  |  |  |  |  |  |  | Yes | 31765706 |  |
| Anaerococcus urinomassiliensis | 1745712 | 2.1901 |  |  |  |  |  |  |  |  |  | Yes | 33514860 | 7846727 |
| Anaerococcus vaginalis | 33037 | 1.9980 |  |  |  |  |  |  |  |  |  | Yes |  |  |
| Anaerolactibacter massiliensis | 2044573 | 3.3017 |  |  |  |  |  |  |  |  |  | Yes | 31011429 | 6462784 |
| Anaerovibrio slackiae | 2652309 | 3.0151 |  |  |  |  |  |  |  |  |  | Yes | 33319778 | 7738495 |
| Anoxybacillus flavithermus | 33934 | 2.7726 |  |  |  |  |  |  |  |  |  | Yes |  |  |
| Aquabacterium parvum | 70584 | 2.9074 |  |  |  |  |  |  |  |  |  | Yes |  |  |
| Aquincola tertiaricarbonis | 391953 | 6.7149 |  |  |  |  |  |  |  |  |  | Yes | 17551046 |  |
| Arcobacter suis | 1278212 | 2.6305 |  |  |  |  |  |  |  |  |  | Yes | 23265195 |  |
| Arsenicicoccus bolidensis | 229480 | 3.9206 |  |  |  |  |  |  |  |  |  | Yes | 15023982 |  |
| Atlantibacter hermannii | 565 | 4.5543 |  |  |  |  |  |  |  |  |  | Yes | 26970508 |  |
| Atopobium deltae | 1393034 | 1.4521 |  |  |  |  |  |  |  |  |  | Yes | 24944340 |  |
| Atopobium vaginae | 82135 | 1.4507 |  |  |  |  |  |  |  |  |  | Yes |  |  |
| Aureimonas altamirensis | 370622 | 4.1910 |  |  |  |  |  |  |  |  |  | Yes | 31240890 | 6660327 |
| Azospirillum lipoferum | 193 | 7.7707 |  |  |  |  |  |  |  |  |  | Yes |  |  |
| Bacillus atrophaeus | 1452 | 4.1294 |  |  |  |  |  |  |  |  |  | Yes |  |  |
| Bacillus cereus | 1396 | 5.7571 |  |  |  |  |  |  |  |  |  | Yes |  |  |
| Bacillus coagulans | 1398 | 3.4053 |  |  |  |  |  |  |  |  |  | Yes |  |  |
| Bacillus hisashii | 996558 | 3.9129 |  |  |  |  |  |  |  |  |  | Yes | 26268484 |  |
| Bacillus intestinalis | 1963032 | 4.0473 |  |  |  |  |  |  |  |  |  | Yes | 28572333 | 5454216 |
| Bacillus methanolicus | 1471 | 3.5583 |  |  |  |  |  |  |  |  |  | Yes | 1380290 |  |
| Bacillus pumilus | 1408 | 3.6656 |  |  |  |  |  |  |  |  |  | Yes | 24031357 | 3769717 |
| Bacillus spizizenii | 96241 | 4.0169 |  |  |  |  |  |  |  |  |  | Yes | 31721032 |  |
| Bacillus subtilis | 1423 | 4.1348 |  |  |  |  |  |  |  |  |  | Yes |  |  |
| Bacillus thermoamylovorans | 35841 | 3.8241 |  |  |  |  |  |  |  |  |  | Yes |  |  |
| Bacteroides caccae | 47678 | 5.3892 |  |  |  |  |  |  |  |  |  | Yes |  |  |
| Bacteroides cellulosilyticus | 246787 | 6.9753 |  |  |  |  |  |  |  |  |  | Yes |  |  |
| Bacteroides coprocola | 310298 | 4.1386 |  |  |  |  |  |  |  |  |  | Yes |  |  |
| Bacteroides coprophilus | 387090 | 3.8527 |  |  |  |  |  |  |  |  |  | Yes |  |  |
| Bacteroides dorei | 357276 | 5.4449 |  |  |  |  |  |  |  |  |  | Yes |  |  |
| Bacteroides eggerthii | 28111 | 4.3035 |  |  |  |  |  |  |  |  |  | Yes |  |  |
| Bacteroides finegoldii | 338188 | 5.0046 |  |  |  |  |  |  |  |  |  | Yes |  |  |
| Bacteroides fragilis | 817 | 5.2708 |  |  |  |  |  |  |  |  |  | Yes |  |  |
| Bacteroides graminisolvens | 477666 | 2.6105 |  |  |  |  |  |  |  |  |  | Yes | 19567576 |  |
| Bacteroides massiliensis | 204516 | 4.4430 |  |  |  |  |  |  |  |  |  | Yes |  |  |
| Bacteroides ovatus | 28116 | 6.7168 |  |  |  |  |  |  |  |  |  | Yes |  |  |
| Bacteroides pyogenes | 310300 | 3.4161 |  |  |  |  |  |  |  |  |  | Yes |  |  |
| Bacteroides stercoris | 46506 | 4.0801 |  |  |  |  |  |  |  |  |  | Yes |  |  |
| Bacteroides vulgatus | 821 | 5.0501 |  |  |  |  |  |  |  |  |  | Yes |  |  |
| Bacteroides xylanisolvens | 371601 | 6.3584 |  |  |  |  |  |  |  |  |  | Yes |  |  |
| Barnesiella intestinihominis | 487174 | 3.1501 |  |  |  |  |  |  |  |  |  | Yes |  |  |
| Bergeyella cardium | 1585976 | 1.9592 |  |  |  |  |  |  |  |  |  | Yes |  |  |
| Bifidobacterium adolescentis | 1680 | 2.2030 |  |  |  |  |  |  |  |  |  | Yes |  |  |
| Bifidobacterium animalis | 28025 | 1.9327 |  |  |  |  |  |  |  |  |  | Yes |  |  |
| Bifidobacterium animalis subsp. lactis HN019 | 486409 | 1.9327 |  |  |  |  |  |  |  |  |  | Yes |  |  |
| Bifidobacterium bifidum | 1681 | 2.2034 |  |  |  |  |  |  |  |  |  | Yes |  |  |
| Bifidobacterium breve | 1685 | 2.3316 |  |  |  |  |  |  |  |  |  | Yes |  |  |
| Bifidobacterium catenulatum | 1686 | 2.0828 |  |  |  |  |  |  |  |  |  | Yes |  |  |
| Bifidobacterium longum | 216816 | 2.3955 |  |  |  |  |  |  |  |  |  | Yes |  |  |
| Bifidobacterium moukalabense | 1333651 | 2.5227 |  |  |  |  |  |  |  |  |  | Yes |  |  |
| Bifidobacterium pseudocatenulatum | 28026 | 2.5227 |  |  |  |  |  |  |  |  |  | Yes |  |  |
| Bifidobacterium pseudolongum | 1694 | 2.0168 |  |  |  |  |  |  |  |  |  | Yes |  |  |
| Bifidobacterium pullorum | 78448 | 2.1010 |  |  |  |  |  |  |  |  |  | Yes |  |  |
| Bifidobacterium scardovii | 158787 | 3.1440 |  |  |  |  |  |  |  |  |  | Yes |  |  |
| Bifidobacterium thermacidophilum | 246618 | 2.2210 |  |  |  |  |  |  |  |  |  | Yes |  |  |
| Bilophila sp. 4_1_30 | 693988 | 4.0137 |  |  |  |  |  |  |  |  |  | Yes |  |  |
| Bilophila wadsworthia | 35833 | 4.3912 |  |  |  |  |  |  |  |  |  | Yes |  |  |
| Blautia obeum | 40520 | 3.6611 |  |  |  |  |  |  |  |  |  | Yes | 25481290 |  |
| Bordetella bronchiseptica | 518 | 5.1999 |  |  |  |  |  |  |  |  |  | Yes |  |  |
| Bordetella hinzii | 103855 | 4.8964 |  |  |  |  |  |  |  |  |  | Yes | 8815118 | 228927 |
| Bordetella pertussis | 520 | 4.1043 |  |  |  |  |  | Yes | Infectious |  |  | Yes | 26906206 | 4846492 |
| Bordetella trematum | 123899 | 4.4453 |  |  |  |  |  |  |  |  |  | Yes | 8863408 |  |
| Brachybacterium massiliense | 1755098 | 3.3629 |  |  |  |  |  |  |  |  |  | Yes | 31463068 | 6710231 |
| Brachybacterium muris | 219301 | 3.2575 |  |  |  |  |  |  |  |  |  | Yes |  |  |
| Brachybacterium paraconglomeratum | 173362 | 3.7842 |  |  |  |  |  |  |  |  |  | Yes |  |  |
| Bradyrhizobium japonicum | 375 | 9.4608 |  |  |  |  |  |  |  |  |  | Yes |  |  |
| Brevibacterium casei | 33889 | 3.8513 |  |  |  |  |  |  |  |  |  | Yes | 15184484, 24648477 | 427857, 3962939 |
| Brevibacterium linens | 1703 | 3.9202 |  |  |  |  |  |  |  |  |  | Yes |  |  |
| Brevibacterium luteolum | 199591 | 3.0428 |  |  |  |  |  |  |  |  |  | Yes | 13130013 |  |
| Brevilactibacter sinopodophylli | 1837344 | 3.0745 |  |  |  |  |  |  |  |  |  | Yes | 28901898 |  |
| Brevundimonas naejangsanensis | 588932 | 3.0000 |  |  |  |  |  |  |  |  |  | Yes |  |  |
| Brevundimonas nasdae | 172043 | 3.4078 |  |  |  |  |  |  |  |  |  | Yes |  |  |
| Brochothrix thermosphacta | 2756 | 2.5456 |  |  |  |  |  |  |  |  |  | Yes |  |  |
| Burkholderia cenocepacia | 95486 | 7.9616 |  |  |  |  |  |  |  |  |  | Yes | 12648723 |  |
| Burkholderia multivorans | 87883 | 6.4468 |  |  |  |  |  |  |  |  |  | Yes | 9336927 |  |
| Butyricicoccus porcorum | 1945634 | 2.5574 |  |  |  |  |  |  |  |  |  | Yes | 29620502 |  |
| Caldibacillus debilis | 301148 | 3.0591 |  |  |  |  |  |  |  |  |  | Yes | 15545458 |  |
| Campylobacter hominis | 76517 | 1.7150 |  |  |  |  |  |  |  |  |  | Yes |  |  |
| Campylobacter jejuni | 197 | 1.6811 |  |  |  |  |  |  |  |  |  | Yes |  |  |
| Campylobacter ureolyticus | 827 | 1.6657 |  |  |  |  |  |  |  |  |  | Yes |  |  |
| Candidatus Endolissoclinum faulkneri | 1263979 | 1.4999 |  |  |  |  |  |  |  |  |  | Yes |  |  |
| Candidatus Fervidibacteria_u_s | 1383058 | 1.0555 |  |  |  |  |  |  |  |  |  | Yes |  |  |
| Candidatus Mycoplasma girerdii | 1318617 | 0.6242 |  |  |  |  |  |  |  |  |  | Yes | 25337710 | 4206474 |
| Candidatus Pantoea carbekii | 1235990 | 1.1740 |  |  |  |  |  |  |  |  |  | Yes | 24874153 |  |
| Candidatus Portiera aleyrodidarum | 91844 | 0.3521 |  |  |  |  |  |  |  |  |  | Yes |  |  |
| Candidatus Riesia_u_s | 401618 | 0.5344 |  |  |  |  |  |  |  |  |  | Yes |  |  |
| Candidatus Saccharimonas aalborgensis | 1332188 | 1.0138 |  |  |  |  |  |  |  |  |  | Yes |  |  |
| Candidatus Sulcia muelleri | 336810 | 0.2700 |  |  |  |  |  |  |  |  |  | Yes |  |  |
| Candidatus Tremblaya phenacola | 1010676 | 0.2189 |  |  |  |  |  |  |  |  |  | Yes | 20851962 | 2976180 |
| Carnobacterium inhibens | 147709 | 2.4971 |  |  |  |  |  |  |  |  |  | Yes |  |  |
| Carnobacterium maltaromaticum | 2751 | 3.5024 |  |  |  |  |  |  |  |  |  | Yes | 17696886 | 2040187 |
| Catabacter hongkongensis | 270498 | 3.1521 |  |  |  |  |  |  |  |  |  | Yes | 17122022 | 1829005 |
| Cellulomonas carbonis | 1386092 | 3.9402 |  |  |  |  |  |  |  |  |  | Yes | 22021576 |  |
| Cellulosimicrobium aquatile | 1612203 | 4.3609 |  |  |  |  |  |  |  |  |  | Yes |  |  |
| Cellulosimicrobium cellulans | 1710 | 4.2751 |  |  |  |  |  |  |  |  |  | Yes |  |  |
| Cellulosimicrobium sp. MM | 1523621 | 4.3220 |  |  |  |  |  |  |  |  |  | Yes |  |  |
| Christensenella minuta | 626937 | 2.9402 |  |  |  |  |  |  |  |  |  | Yes |  |  |
| Christensenella timonensis | 1816678 | 2.6509 |  |  |  |  |  |  |  |  |  | Yes |  |  |
| Chryseobacterium gleum | 250 | 5.2007 |  |  |  |  |  |  |  |  |  | Yes |  |  |
| Chryseobacterium haifense | 421525 | 2.8457 |  |  |  |  |  |  |  |  |  | Yes | 17911308 |  |
| Citricoccus sp. CH26A | 1045009 | 3.5613 |  |  |  |  |  |  |  |  |  | Yes |  |  |
| Citrobacter freundii | 546 | 5.2576 |  |  |  |  |  |  |  |  |  | Yes |  |  |
| Citrobacter portucalensis | 1639133 | 5.1114 |  |  |  |  |  |  |  |  |  | Yes | 28857032 |  |
| Clostridioides difficile | 1496 | 4.1534 |  |  |  |  |  |  |  |  |  | Yes |  |  |
| Clostridium perfringens | 1502 | 3.3457 |  |  |  |  |  |  |  |  |  | Yes |  |  |
| Collinsella aerofaciens | 74426 | 2.2041 |  |  |  |  |  |  |  |  |  | Yes |  |  |
| Collinsella intestinalis | 147207 | 1.8051 |  |  |  |  |  |  |  |  |  | Yes | 11034485 |  |
| Comamonas aquatica | 225991 | 3.9155 |  |  |  |  |  |  |  |  |  | Yes | 12807213 |  |
| Comamonas testosteroni | 285 | 5.6494 |  |  |  |  |  |  |  |  |  | Yes |  |  |
| Companilactobacillus paralimentarius | 83526 | 2.7270 |  |  |  |  |  |  |  |  |  | Yes | 32760353 | 7374928 |
| Conchiformibius kuhniae | 1121351 | 2.1186 |  |  |  |  |  |  |  |  |  | Yes |  |  |
| Coprococcus catus | 116085 | 3.5223 |  |  |  |  |  |  |  |  |  | Yes |  |  |
| Coprococcus comes | 410072 | 3.3395 |  |  |  |  |  |  |  |  |  | Yes |  |  |
| Coprococcus sp. ART55/1 | 751585 | 2.9105 |  |  |  |  |  |  |  |  |  | Yes |  |  |
| Coriobacteriaceae bacterium BV3Ac1 | 1111135 | 1.8014 |  |  |  |  |  |  |  |  |  | Yes |  |  |
| Corynebacterium accolens | 38284 | 2.4656 |  |  |  |  |  |  |  |  |  | Yes | 26733066 | 4725001 |
| Corynebacterium appendicis | 163202 | 2.2481 |  |  |  |  |  |  |  |  |  | Yes | 12148623 |  |
| Corynebacterium argentoratense | 42817 | 2.0209 |  |  |  |  |  | Yes | Opportunistic |  |  | Yes | 26933505 | 4765771 |
| Corynebacterium bovis | 36808 | 2.5916 |  |  |  |  |  |  |  |  |  | Yes |  |  |
| Corynebacterium crenatum | 168810 | 3.3077 |  |  |  |  |  |  |  |  |  | Yes |  |  |
| Corynebacterium falsenii | 108486 | 2.3818 |  |  |  |  |  |  |  |  |  | Yes |  |  |
| Corynebacterium freneyi | 134034 | 2.8818 |  |  |  |  |  |  |  |  |  | Yes | 11594602 |  |
| Corynebacterium genitalium | 38288 | 2.3336 |  |  |  |  |  |  |  |  |  | Yes |  |  |
| Corynebacterium hadale | 2026255 | 2.5643 |  |  |  |  |  |  |  |  |  | Yes | 29557772 |  |
| Corynebacterium ihumii | 1232427 | 2.2513 |  |  |  |  |  |  |  |  |  | Yes | 25197488 | 4149009 |
| Corynebacterium imitans | 156978 | 2.5655 |  |  |  |  |  | Yes | Infectious | No | No | Yes | 9230366 | 229887 |
| Corynebacterium jeddahense | 1414719 | 2.4721 |  |  |  |  |  |  |  |  |  | Yes | 25197478 | 4149016 |
| Corynebacterium jeikeium | 38289 | 2.4147 |  |  |  |  |  | Yes | Opportunistic |  |  | Yes |  |  |
| Corynebacterium kroppenstedtii | 161879 | 2.5153 |  |  |  |  |  |  |  |  |  | Yes |  |  |
| Corynebacterium lipophiloflavum | 161889 | 2.3858 |  |  |  |  |  |  |  |  |  | Yes |  |  |
| Corynebacterium maris | 575200 | 2.8336 |  |  |  |  |  |  |  |  |  | Yes | 19622641 |  |
| Corynebacterium minutissimum | 38301 | 2.6960 |  |  |  |  |  | Yes | Infectious |  |  | Yes |  |  |
| Corynebacterium mucifaciens | 57171 | 2.1857 |  |  |  |  |  |  |  |  |  | Yes |  |  |
| Corynebacterium propinquum | 43769 | 2.5531 |  |  |  |  |  |  |  |  |  | Yes |  |  |
| Corynebacterium pseudodiphtheriticum | 37637 | 2.3245 |  |  |  |  | Yes |  |  |  |  | Yes | 26252066 | 4720244 |
| Corynebacterium pseudogenitalium | 38303 | 2.6014 |  |  |  |  |  |  |  |  |  | Yes |  |  |
| Corynebacterium singulare | 161899 | 2.8305 |  |  |  |  |  |  |  |  |  | Yes | 9336912 |  |
| Corynebacterium tuberculostearicum | 38304 | 2.3723 |  |  |  |  |  |  |  |  |  | Yes |  |  |
| Corynebacterium tuscaniense | 302449 | 2.2413 |  |  |  |  |  |  |  |  |  | Yes | 16455875 | 1392639 |
| Corynebacterium urealyticum | 43771 | 2.3692 |  |  |  |  |  |  |  |  |  | Yes |  |  |
| Corynebacterium ureicelerivorans | 401472 | 2.3283 |  |  |  |  |  |  |  |  |  | Yes | 17551029 |  |
| Corynebacterium variabile | 1727 | 3.1891 |  |  |  |  |  |  |  |  |  | Yes |  |  |
| Cronobacter condimenti | 1163710 | 4.4995 |  |  |  |  |  |  |  |  |  | Yes | 22661070 |  |
| Cronobacter dublinensis | 413497 | 4.5343 |  |  |  |  |  |  |  |  |  | Yes | 18523192 |  |
| Cronobacter malonaticus | 413503 | 4.4483 |  |  |  |  |  |  |  |  |  | Yes | 18523192 |  |
| Cronobacter muytjensii | 413501 | 4.3545 |  |  |  |  |  |  |  |  |  | Yes | 18523192 |  |
| Cronobacter sakazakii | 28141 | 4.4734 |  |  |  |  |  |  |  |  |  | Yes | 18523192 |  |
| Cronobacter turicensis | 413502 | 4.4936 |  |  |  |  |  |  |  |  |  | Yes | 18523192 |  |
| Cronobacter universalis | 535744 | 4.4369 |  |  |  |  |  |  |  |  |  | Yes | 22661070 |  |
| Cupriavidus basilensis | 68895 | 8.4839 |  |  |  |  |  |  |  |  |  | Yes |  |  |
| Cutibacterium avidum | 33010 | 2.5406 |  |  |  |  |  |  |  |  |  | Yes |  |  |
| Cutibacterium granulosum | 33011 | 2.1343 |  |  |  |  |  |  |  |  |  | Yes |  |  |
| Cutibacterium namnetense | 1574624 | 2.3866 |  |  |  |  |  |  |  |  |  | Yes |  |  |
| Dankookia rubra | 1442381 | 7.7823 |  |  |  |  |  |  |  |  |  | Yes | 27225458 |  |
| Dechloromonas agitata | 73030 | 3.3057 |  |  |  |  |  |  |  |  |  | Yes | 11321099 |  |
| Dehalococcoides mccartyi | 61435 | 1.3889 |  |  |  |  |  |  |  |  |  | Yes |  |  |
| Deinococcus swuensis | 1182571 | 3.5314 |  |  |  |  |  |  |  |  |  | Yes |  |  |
| Deinococcus wulumuqiensis | 980427 | 3.5060 |  |  |  |  |  |  |  |  |  | Yes | 19801390 |  |
| Delftia acidovorans | 80866 | 6.5494 |  |  |  |  |  |  |  |  |  | Yes |  |  |
| Dermabacter hominis | 36740 | 2.2819 |  |  |  |  |  |  |  |  |  | Yes | 25356327 | 4184692 |
| Dermabacter jinjuensis | 1667168 | 2.3777 |  |  |  |  |  |  |  |  |  | Yes | 27088668 |  |
| Dermacoccus nishinomiyaensis | 1274 | 3.0981 |  |  |  |  |  |  |  |  |  | Yes |  |  |
| Desulfovibrio desulfuricans | 876 | 3.2514 |  |  |  |  |  |  |  |  |  | Yes |  |  |
| Desulfovibrio porci | 2605782 | 3.3112 |  |  |  |  |  |  |  |  |  | Yes | 33319778 | 7738495 |
| Devosia elaeis | 1770058 | 3.8782 |  |  |  |  |  |  |  |  |  | Yes | 27902276 |  |
| Devosia limi | 288995 | 4.3860 |  |  |  |  |  |  |  |  |  | Yes | 16166701 |  |
| Dietzia cinnamea | 321318 | 3.5907 |  |  |  |  |  |  |  |  |  | Yes |  |  |
| Dietzia kunjamensis | 322509 | 3.5443 |  |  |  |  |  |  |  |  |  | Yes | 16825647 |  |
| Dietzia maris | 37915 | 3.2428 |  |  |  |  |  |  |  |  |  | Yes |  |  |
| Dolosigranulum pigrum | 29394 | 1.9206 |  |  |  |  |  |  |  |  |  | Yes | 8294309 |  |
| Dorea longicatena | 88431 | 3.1266 |  |  |  |  |  |  |  |  |  | Yes |  |  |
| Duganella zoogloeoides | 75659 | 6.2730 |  |  |  |  |  |  |  |  |  | Yes | 9336937 |  |
| Duncaniella freteri | 2530391 | 3.5519 |  |  |  |  |  |  |  |  |  | Yes | 32250242 |  |
| Duncaniella muris | 2094150 | 3.2688 |  |  |  |  |  |  |  |  |  | Yes |  |  |
| Eggerthella lenta | 84112 | 3.4231 |  |  |  |  |  |  |  |  |  | Yes |  |  |
| Eikenella exigua | 2528037 | 1.9540 |  |  |  |  |  |  |  |  | No | Yes |  |  |
| Eikenella halliae | 1795832 | 2.5456 |  |  |  |  |  |  |  |  |  | Yes |  |  |
| Eikenella longinqua | 1795827 | 2.2359 |  |  |  |  |  |  |  |  |  | Yes |  |  |
| Elizabethkingia anophelis | 1117645 | 4.0580 |  |  |  |  |  |  |  |  |  | Yes | 21169462 |  |
| Elizabethkingia bruuniana | 1756149 | 4.3507 |  |  |  |  |  |  |  |  |  | Yes | 28856455 | 5862389 |
| Enhydrobacter aerosaccus | 225324 | 6.7698 |  |  |  |  |  |  |  |  |  | Yes |  |  |
| Enorma burkinafasonensis | 2590867 | 2.0968 |  |  |  |  |  |  |  |  |  | Yes | 32528685 | 7283140 |
| Enterobacter asburiae | 61645 | 4.9014 |  |  |  |  |  |  |  |  |  | Yes | 3711302 | 268805 |
| Enterobacter cloacae | 550 | 4.9591 |  |  |  |  |  |  |  |  |  | Yes |  |  |
| Enterobacter cloacae complex | 354276 | 4.9591 |  |  |  |  |  |  |  |  |  | Yes |  |  |
| Enterobacter hormaechei | 158836 | 4.9600 |  |  |  |  |  |  |  |  |  | Yes |  |  |
| Enterobacter ludwigii | 299767 | 4.9096 |  |  |  |  |  |  |  |  |  | Yes |  |  |
| Enterobacter mori | 539813 | 4.9453 |  |  |  |  |  |  |  |  |  | Yes | 21216919 |  |
| Enterobacter roggenkampii | 1812935 | 4.9766 |  |  |  |  |  |  |  |  |  | Yes | 30430006 | 6097438 |
| Enterococcus faecalis | 1351 | 2.9686 |  |  |  |  |  |  |  |  |  | Yes |  |  |
| Enterococcus faecium | 1352 | 2.9207 |  |  |  |  |  | Yes | Infectious |  |  | Yes |  |  |
| Enterococcus gilvus | 160453 | 4.1368 |  |  |  |  |  |  |  |  |  | Yes |  |  |
| Enterorhabdus caecimuris | 671266 | 2.8874 |  |  |  |  |  |  |  |  |  | Yes |  |  |
| Enterorhabdus mucosicola | 580026 | 2.9480 |  |  |  |  |  |  |  |  |  | Yes |  |  |
| Escherichia albertii | 208962 | 4.8387 |  |  |  |  |  |  |  |  |  | Yes | 29249728 | 5797873 |
| Escherichia coli | 562 | 5.1417 |  |  |  |  | Yes | Yes | Opportunistic | No |  | Yes |  |  |
| Evtepia gabavorous | 2211183 | 2.4944 |  |  |  |  |  |  |  |  |  | Yes |  |  |
| Ewingella americana | 41202 | 4.9418 |  |  |  |  |  | Yes | Infectious |  |  | Yes |  |  |
| Facklamia languida | 82347 | 1.7178 |  |  |  |  |  |  |  |  |  | Yes | 10074542 | 88665 |
| Faecalibacterium prausnitzii | 853 | 2.9698 |  |  |  |  |  |  |  |  |  | Yes |  |  |
| Faecalibacterium rodentium | 1702221 | 2.4158 |  |  |  |  |  |  |  |  |  | Yes |  |  |
| Fannyhessea vaginae | 82135 | 1.4186 |  |  |  |  |  |  |  |  |  | Yes |  |  |
| Finegoldia magna | 1260 | 1.8999 |  |  |  |  |  |  |  |  |  | Yes |  |  |
| Fusobacterium russii | 854 | 1.9418 |  |  |  |  |  |  |  |  |  | Yes | 12173104 |  |
| Gallibacterium anatis | 1195244 | 2.4980 |  |  |  |  |  |  |  |  |  | Yes |  |  |
| Gallibacterium genomosp. 1 | 155515 | 2.3758 |  |  |  |  |  |  |  |  |  | Yes |  |  |
| Gardnerella vaginalis | 2702 | 1.6203 |  |  |  |  |  |  |  |  |  | Yes |  |  |
| Gemella asaccharolytica | 502393 | 1.2899 |  |  |  |  |  |  |  |  |  | Yes |  |  |
| Gemella bergeri | 84136 | 1.6045 |  |  |  |  |  |  |  |  |  | Yes |  |  |
| Gemella cuniculi | 1121914 | 1.8697 |  |  |  |  |  |  |  |  |  | Yes |  |  |
| Gemmata obscuriglobus | 114 | 9.0159 |  |  |  |  |  |  |  |  |  | Yes |  |  |
| Gemmatirosa kalamazoonesis | 861299 | 7.4792 |  |  |  |  |  |  |  |  |  | Yes |  |  |
| Gemmiger formicilis | 745368 | 2.3639 |  |  |  |  |  |  |  |  |  | Yes |  |  |
| Geodermatophilus obscurus | 1861 | 5.2386 |  |  |  |  |  |  |  |  |  | Yes | 21304698 | 3035280 |
| Glaesserella parasuis | 738 | 2.2215 |  |  |  |  |  |  |  |  |  | Yes |  |  |
| Glutamicibacter soli | 453836 | 3.9033 |  |  |  |  |  |  |  |  |  | Yes | 18337691 |  |
| Glycomyces tenuis | 58116 | 5.8840 |  |  |  |  |  |  |  |  |  | Yes |  |  |
| Gordonia bronchialis | 2054 | 5.2990 |  |  |  |  |  |  |  |  |  | Yes |  |  |
| Gordonia jacobaea | 122202 | 4.9255 |  |  |  |  |  |  |  |  |  | Yes | 26337890 | 4559739 |
| Gordonia sputi | 36823 | 4.9530 |  |  |  |  |  |  |  |  |  | Yes | 19788838 | 2819866 |
| Gordonia terrae | 2055 | 5.6733 |  |  |  |  |  |  |  |  |  | Yes |  |  |
| Gracilibacteria bacterium JGI 0000069-P22 | 1130343 | 0.3410 |  |  |  |  |  |  |  |  |  | Yes |  |  |
| Haemophilus aegyptius | 197575 | 1.9262 |  |  |  |  |  | Yes | Opportunistic |  |  | Yes |  |  |
| Haemophilus parasuis | 738 | 2.2215 |  |  |  |  |  |  |  |  |  | Yes |  |  |
| Haemophilus quentini | 123834 | 2.1376 |  |  |  |  |  |  |  |  |  | Yes | 31578259 | 6879273 |
| Hafnia alvei | 569 | 4.7902 |  |  |  |  |  |  |  |  |  | Yes | 8783707 |  |
| Hafnia paralvei | 546367 | 4.7092 |  |  |  |  |  |  |  |  |  | Yes | 19734282 |  |
| Halobacterium salinarum | 2242 | 2.4297 |  |  |  |  |  |  |  |  |  | Yes |  |  |
| Halomonas desiderata | 52021 | 4.7226 |  |  |  |  |  |  |  |  |  | Yes |  |  |
| Halorubrum distributum | 29283 | 3.3061 |  |  |  |  |  |  |  |  |  | Yes |  |  |
| Hassallia byssoidea | 482630 | 7.5015 |  |  |  |  |  |  |  |  |  | Yes |  |  |
| Helcococcus ovis | 72026 | 1.7735 |  |  |  |  |  |  |  |  |  | Yes | 10555322 |  |
| Heminiphilus faecis | 2601703 | 3.0526 |  |  |  |  |  |  |  |  |  | Yes | 33566238 |  |
| Herbaspirillum huttiense | 863372 | 5.6918 |  |  |  |  |  |  |  |  |  | Yes |  |  |
| Hornefia porci | 2652292 | 2.8145 |  |  |  |  |  |  |  |  |  | Yes | 33319778 | 7738495 |
| Hydrocarboniphaga effusa | 243629 | 3.0241 |  |  |  |  |  |  |  |  |  | Yes |  |  |
| Hylemonella gracilis | 80880 | 3.8216 |  |  |  |  |  |  |  |  |  | Yes |  |  |
| Hymenobacter aerophilus | 119644 | 4.2530 |  |  |  |  |  |  |  |  |  | Yes |  |  |
| Inquilinus limosus | 171674 | 7.4137 |  |  |  |  |  |  |  |  |  | Yes |  |  |
| Intestinibacter bartlettii | 261299 | 2.9762 |  |  |  |  |  |  |  |  |  | Yes |  |  |
| Janibacter hoylei | 364298 | 3.1225 |  |  |  |  |  |  |  |  |  | Yes |  |  |
| Janibacter indicus | 857417 | 3.4195 |  |  |  |  |  |  |  |  |  | Yes | 24744020 |  |
| Janibacter melonis | 262209 | 3.4828 |  |  |  |  |  |  |  |  |  | Yes | 15545420 |  |
| Janthinobacterium lividum | 29581 | 6.3703 |  |  |  |  |  |  |  |  |  | Yes |  |  |
| Jeotgalicoccus psychrophilus | 157228 | 2.3457 |  |  |  |  |  |  |  |  |  | Yes | 12710632 |  |
| Jonesia quinghaiensis | 262806 | 3.0381 |  |  |  |  |  |  |  |  |  | Yes | 15545455 |  |
| Jonquetella anthropi | 645512 | 1.7330 |  |  |  |  |  |  |  |  |  | Yes |  |  |
| Kingella kingae | 504 | 1.9929 |  |  |  |  |  |  |  |  |  | Yes |  |  |
| Kingella potus | 265175 | 2.4677 |  |  |  |  |  |  |  |  |  | Yes | 16000497 | 1169176 |
| Klebsiella aerogenes | 548 | 5.2607 |  |  |  |  |  |  |  |  |  | Yes |  |  |
| Klebsiella cf. planticola B43 | 95610 | 4.4793 |  |  |  |  |  |  |  |  |  | Yes | 23754693 |  |
| Klebsiella grimontii | 2058152 | 6.1491 |  |  |  |  |  |  |  |  |  | Yes |  |  |
| Klebsiella michiganensis | 1134687 | 6.1921 |  |  |  |  |  |  |  |  |  | Yes |  |  |
| Klebsiella oxytoca | 571 | 6.0490 |  |  |  |  |  |  |  |  |  | Yes |  |  |
| Klebsiella pneumoniae | 573 | 5.5873 |  |  |  |  |  |  |  |  |  | Yes |  |  |
| Klebsiella variicola | 244366 | 5.6685 |  |  |  |  |  |  |  |  |  | Yes |  |  |
| Klenkia marina | 1960309 | 4.2192 |  |  |  |  |  |  |  |  |  | Yes | 29312207 | 5742155 |
| Knoellia aerolata | 442954 | 4.0880 |  |  |  |  |  |  |  |  |  | Yes | 18048739 |  |
| Kocuria indica | 1049583 | 2.8783 |  |  |  |  |  |  |  |  |  | Yes | 24254742 |  |
| Kocuria marina | 223184 | 2.9614 |  |  |  |  |  |  |  |  |  | Yes | 15388718 |  |
| Kocuria palustris | 71999 | 2.8439 |  |  |  |  |  |  |  |  |  | Yes |  |  |
| Kocuria polaris | 136273 | 3.8068 |  |  |  |  |  |  |  |  |  | Yes |  |  |
| Kocuria rhizophila | 72000 | 2.7016 |  |  |  |  |  |  |  |  |  | Yes |  |  |
| Kocuria rosea | 1275 | 3.9418 |  |  |  |  |  |  |  |  |  | Yes | 26038440 | 3826069 |
| Kocuria varians | 1272 | 2.8436 |  |  |  |  |  |  |  |  |  | Yes |  |  |
| Kosakonia cowanii | 208223 | 4.8085 |  |  |  |  |  |  |  |  |  | Yes | 32301994 |  |
| Kytococcus schroeteri | 138300 | 2.4336 |  |  |  |  |  |  |  |  |  | Yes | 12361263 |  |
| Kytococcus sedentarius | 1276 | 2.6189 |  |  |  |  |  |  |  |  |  | Yes |  |  |
| Lachnoanaerobaculum umeaense | 617123 | 2.8104 |  |  |  |  |  |  |  |  |  | Yes |  |  |
| Lachnospira eligens | 39485 | 3.0358 |  |  |  |  |  |  |  |  |  | Yes |  |  |
| Lacticaseibacillus chiayiensis | 2100821 | 2.8554 |  |  |  |  |  |  |  |  |  | Yes | 29456028 |  |
| Lacticaseibacillus zeae | 57037 | 3.0909 |  |  |  |  |  |  |  |  |  | Yes | 33112225 |  |
| Lactobacillus acidophilus | 1579 | 1.9707 |  |  |  |  |  |  |  |  |  | Yes |  |  |
| Lactobacillus amylolyticus | 83683 | 1.5710 |  |  |  |  |  |  |  |  |  | Yes | 9779604 |  |
| Lactobacillus amylovorus | 1604 | 2.0174 |  |  |  |  |  |  |  |  |  | Yes |  |  |
| Lactobacillus antri | 227943 | 2.2418 |  |  |  |  |  |  |  |  |  | Yes |  |  |
| Lactobacillus coleohominis | 575594 | 1.7210 |  |  |  |  |  |  |  |  |  | Yes |  |  |
| Lactobacillus crispatus | 47770 | 2.1640 |  |  |  |  |  |  |  |  |  | Yes |  |  |
| Lactobacillus curvatus | 28038 | 1.9326 |  |  |  |  |  |  |  |  |  | Yes |  |  |
| Lactobacillus delbrueckii | 1584 | 1.8751 |  |  |  |  |  |  |  |  |  | Yes |  |  |
| Lactobacillus gallinarum | 1423748 | 2.0205 |  |  |  |  |  |  |  |  |  | Yes |  |  |
| Lactobacillus gastricus | 227942 | 1.9049 |  |  |  |  |  |  |  |  |  | Yes |  |  |
| Lactobacillus helveticus | 1587 | 2.0587 |  |  |  |  |  |  |  |  |  | Yes |  |  |
| Lactobacillus hominis | 1423758 | 1.9263 |  |  |  |  |  |  |  |  |  | Yes |  |  |
| Lactobacillus iners | 147802 | 1.2825 |  |  |  |  |  |  |  |  |  | Yes |  |  |
| Lactobacillus jensenii | 575606 | 1.6579 |  |  |  |  |  |  |  |  |  | Yes |  |  |
| Lactobacillus johnsonii | 33959 | 1.9181 |  |  |  |  |  |  |  |  |  | Yes |  |  |
| Lactobacillus kalixensis | 227944 | 2.0782 |  |  |  |  |  |  |  |  |  | Yes | 15653856 |  |
| Lactobacillus kitasatonis | 1423767 | 1.9069 |  |  |  |  |  |  |  |  |  | Yes |  |  |
| Lactobacillus mucosae | 97478 | 2.0791 |  |  |  |  |  |  |  |  |  | Yes |  |  |
| Lactobacillus murinus | 1622 | 2.3915 |  |  |  |  |  |  |  |  |  | Yes |  |  |
| Lactobacillus paragasseri | 2107999 | 1.9567 |  |  |  |  |  |  |  |  |  | Yes | 35694384 | 9186330 |
| Lactobacillus plantarum | 1590 | 3.2448 |  |  |  |  |  |  |  |  |  | Yes |  |  |
| Lactobacillus psittaci | 1122152 | 1.5421 |  |  |  |  |  |  |  |  |  | Yes |  |  |
| Lactobacillus reuteri | 1598 | 2.1153 |  |  |  |  |  |  |  |  |  | Yes |  |  |
| Lactobacillus sakei | 1599 | 1.9926 |  |  |  |  |  |  |  |  |  | Yes |  |  |
| Lactobacillus taiwanensis | 508451 | 1.9867 |  |  |  |  |  |  |  |  |  | Yes | 19605711 |  |
| Lactobacillus ultunensis | 227945 | 2.2484 |  |  |  |  |  |  |  |  |  | Yes |  |  |
| Lactococcus chungangensis | 451457 | 2.2092 |  |  |  |  |  |  |  |  |  | Yes | 18676466 |  |
| Lactococcus garvieae | 1363 | 2.0549 |  |  |  |  |  |  |  |  |  | Yes |  |  |
| Lactococcus lactis | 1358 | 2.5029 |  |  |  |  |  |  |  |  |  | Yes | 25758458 | PMC4547204 |
| Lawsonella clevelandensis | 1528099 | 1.8688 |  |  |  |  |  |  |  |  |  | Yes |  |  |
| Leclercia adecarboxylata | 83655 | 4.6076 |  |  |  |  |  |  |  |  |  | Yes |  |  |
| Lelliottia amnigena | 61646 | 4.5618 |  |  |  |  |  |  |  |  |  | Yes | 23632228 |  |
| Lelliottia nimipressuralis | 69220 | 4.6329 |  |  |  |  |  |  |  |  |  | Yes | 23632228 |  |
| Lentibacillus lipolyticus | 2510307 | 3.1467 |  |  |  |  |  |  |  |  |  | Yes | 31424384 |  |
| Lentilactobacillus parabuchneri | 152331 | 2.6151 |  |  |  |  |  |  |  |  |  | Yes |  |  |
| Leptolyngbya boryana | 1184 | 7.0186 |  |  |  |  |  |  |  |  |  | Yes |  |  |
| Leucobacter chironomi | 491918 | 2.9647 |  |  |  |  |  |  |  |  |  | Yes |  |  |
| Leucobacter luti | 340320 | 3.6182 |  |  |  |  |  |  |  |  |  | Yes |  |  |
| Leucobacter muris | 1935379 | 3.2120 |  |  |  |  |  |  |  |  |  | Yes | 31099739 |  |
| Leucobacter salsicius | 664638 | 3.1854 |  |  |  |  |  |  |  |  |  | Yes | 20363927 |  |
| Leuconostoc carnosum | 1252 | 1.7713 |  |  |  |  |  |  |  |  |  | Yes |  |  |
| Leuconostoc inhae | 178001 | 2.0581 |  |  |  |  |  |  |  |  |  | Yes | 12892137 |  |
| Leuconostoc lactis | 1246 | 1.7207 |  |  |  |  |  |  |  |  |  | Yes |  |  |
| Leuconostoc mesenteroides | 1245 | 1.9064 |  |  |  |  |  |  |  |  |  | Yes |  |  |
| Leuconostoc pseudomesenteroides | 33968 | 2.0086 |  |  |  |  |  |  |  |  |  | Yes |  |  |
| Ligilactobacillus ruminis | 1623 | 2.1087 |  |  |  |  |  |  |  |  |  | Yes | 25879663 | 4393605 |
| Limosilactobacillus gorillae | 1450649 | 1.6416 |  |  |  |  |  |  |  |  |  | Yes | 25240022 |  |
| Limosilactobacillus panis | 47493 | 2.0828 |  |  |  |  |  |  |  |  |  | Yes | 8934903 |  |
| Limosilactobacillus secaliphilus | 396268 | 1.6461 |  |  |  |  |  |  |  |  |  | Yes | 17392199 |  |
| Listeria monocytogenes | 1639 | 2.9722 |  |  |  |  |  |  |  |  |  | Yes |  |  |
| Luteimonas huabeiensis | 1244513 | 4.2959 |  |  |  |  |  |  |  |  |  | Yes | 23504969 |  |
| Lysobacter oculi | 2698682 | 2.5928 |  |  |  |  |  |  |  |  |  | Yes | 31587117 |  |
| Massilia oculi | 945844 | 5.8447 |  |  |  |  |  |  |  |  |  | Yes | 21441374 |  |
| Massilia timonae | 47229 | 5.3054 |  |  |  |  |  |  |  |  |  | Yes |  |  |
| Megasphaera elsdenii | 907 | 2.2883 |  |  |  |  |  |  |  |  |  | Yes | 21914887 | 3187434 |
| Meiothermus cerbereus | 65552 | 3.0348 |  |  |  |  |  |  |  |  |  | Yes | 9336933 |  |
| Meiothermus ruber | 277 | 3.0293 |  |  |  |  |  |  |  |  |  | Yes |  |  |
| Meiothermus silvanus | 52022 | 3.0687 |  |  |  |  |  |  |  |  |  | Yes | 21304690 | 3035272 |
| Melaminivora alkalimesophila | 1165852 | 2.9781 |  |  |  |  |  |  |  |  |  | Yes | 24591424 |  |
| Metaprevotella massiliensis | 1870999 | 0.1850 |  |  |  |  |  |  |  |  |  | Yes |  |  |
| Methanobrevibacter smithii | 2173 | 1.7912 |  |  |  |  |  |  |  |  |  | Yes | 29449383 | 5814497 |
| Methylobacterium aquaticum | 270351 | 7.4253 |  |  |  |  |  |  |  |  |  | Yes |  |  |
| Methylobacterium brachiatum | 269660 | 6.3396 |  |  |  |  |  |  |  |  |  | Yes |  |  |
| Methylobacterium oryzae | 334852 | 6.2866 |  |  |  |  |  |  |  |  |  | Yes |  |  |
| Methylobacterium populi | 223967 | 5.7746 |  |  |  |  |  |  |  |  |  | Yes |  |  |
| Methylobacterium pseudosasicola | 582667 | 6.8453 |  |  |  |  |  |  |  |  |  | Yes |  |  |
| Methylobacterium radiotolerans | 31998 | 6.7912 |  |  |  |  |  |  |  |  |  | Yes |  |  |
| Methylobacterium sp. EUR3 AL-11 | 1305730 | 5.5055 |  |  |  |  |  |  |  |  |  | Yes |  |  |
| Methylobacterium sp. UNCCL110 | 1449057 | 5.5055 |  |  |  |  |  |  |  |  |  | Yes |  |  |
| Microbacterium aurum | 36805 | 3.4125 |  |  |  |  |  |  |  |  |  | Yes |  |  |
| Microbacterium lacticum | 33885 | 2.9848 |  |  |  |  |  |  |  |  |  | Yes |  |  |
| Microbacterium laevaniformans | 36807 | 3.1156 |  |  |  |  |  |  |  |  |  | Yes |  |  |
| Microbacterium sp. KROCY2 | 1305732 | 3.5262 |  |  |  |  |  |  |  |  |  | Yes |  |  |
| Micrococcus aloeverae | 1391911 | 2.4934 |  |  |  |  |  |  |  |  |  | Yes |  |  |
| Micrococcus flavus | 384602 | 2.5536 |  |  |  |  |  |  |  |  |  | Yes | 17220443 |  |
| Micrococcus luteus | 1270 | 2.5013 |  |  |  |  |  |  |  |  |  | Yes |  |  |
| Micrococcus lylae | 1273 | 2.6645 |  |  |  |  |  |  |  |  |  | Yes |  |  |
| Micrococcus terreus | 574650 | 3.0875 |  |  |  |  |  |  |  |  |  | Yes | 19783614 |  |
| Micrococcus yunnanensis | 566027 | 2.4779 |  |  |  |  |  |  |  |  |  | Yes | 19620366 |  |
| Micromonospora globbae | 1894969 | 7.0872 |  |  |  |  |  |  |  |  |  | Yes | 29458491 |  |
| Mitsuokella jalaludinii | 187979 | 2.3555 |  |  |  |  |  |  |  |  |  | Yes | 12054230 |  |
| Mixta calida | 665913 | 4.3267 |  |  |  |  |  |  |  |  |  | Yes | 29485394 |  |
| Mobiluncus curtisii | 2051 | 2.1530 |  |  |  |  |  |  |  |  |  | Yes |  |  |
| Modestobacter caceresii | 1522368 | 4.9537 |  |  |  |  |  |  |  |  |  | Yes | 27108251 |  |
| Moraxella atlantae | 34059 | 2.3490 |  |  |  |  |  |  |  |  |  | Yes |  |  |
| Moraxella catarrhalis | 480 | 1.9091 |  |  |  |  |  |  |  |  |  | Yes | 19480579 |  |
| Moraxella lincolnii | 90241 | 2.0769 |  |  |  |  |  |  |  |  |  | Yes | 8347507 |  |
| Moraxella osloensis | 34062 | 2.6313 |  |  |  |  |  |  |  |  |  | Yes |  |  |
| Muribaculum intestinale | 1796646 | 3.2844 |  |  |  |  |  |  |  |  |  | Yes | 27670113 |  |
| Mycobacterium gordonae | 1778 | 7.3066 |  |  |  |  |  |  |  |  |  | Yes |  |  |
| Mycobacterium iranicum | 912594 | 6.3357 |  |  |  |  |  |  |  |  |  | Yes |  |  |
| Mycobacterium tuberculosis | 1773 | 4.3831 |  |  |  |  |  |  |  |  |  | Yes |  |  |
| Mycobacteroides abscessus | 36809 | 5.0944 |  |  |  |  |  |  |  |  |  | Yes | 27499141 |  |
| Mycolicibacterium chlorophenolicum | 37916 | 7.3302 |  |  |  |  |  |  |  |  |  | Yes |  |  |
| Mycolicibacterium llatzerense | 280871 | 5.8381 |  |  |  |  |  |  |  |  |  | Yes | 19060055 |  |
| Mycolicibacterium phocaicum | 319706 | 5.8532 |  |  |  |  |  |  |  |  |  | Yes | 34118676 |  |
| Mycoplasma hominis | 2098 | 0.6795 |  |  |  |  |  |  |  |  |  | Yes |  |  |
| Nakamurella multipartita | 53461 | 6.0603 |  |  |  |  |  |  |  |  |  | Yes |  |  |
| Naumannella halotolerans | 993414 | 3.4740 |  |  |  |  |  |  |  |  |  | Yes | 22307502 |  |
| Necropsobacter massiliensis | 1400001 | 2.4934 |  |  |  |  |  |  |  |  |  | Yes |  |  |
| Negativicoccus succinicivorans | 620903 | 1.4581 |  |  |  |  |  |  |  |  |  | Yes |  |  |
| Neisseria canis | 493 | 2.5492 |  |  |  |  |  |  |  |  |  | Yes |  |  |
| Neisseria chenwenguii | 1853278 | 2.4742 |  |  |  |  |  |  |  |  |  | Yes | 30798492 | 6546665 |
| Neisseria dentiae | 194197 | 2.7588 |  |  |  |  |  |  |  |  |  | Yes | 8987719 |  |
| Neisseria iguanae | 90242 | 2.6102 |  |  |  |  |  |  |  |  |  | Yes |  |  |
| Neisseria shayeganii | 607712 | 2.3546 |  |  |  |  |  |  |  |  |  | Yes |  |  |
| Neisseria wadsworthii | 1030841 | 2.4671 |  |  |  |  |  |  |  |  |  | Yes |  |  |
| Neisseria weaveri | 28091 | 2.1655 |  |  |  |  |  |  |  |  |  | Yes |  |  |
| Nesterenkonia massiliensis | 1232429 | 2.6724 |  |  |  |  |  |  |  |  |  | Yes | 25197469 | 4148991 |
| Nitrosomonas eutropha | 916 | 2.5200 |  |  |  |  |  |  |  |  |  | Yes |  |  |
| Oceanivirga miroungae | 1130046 | 1.3570 |  |  |  |  |  |  |  |  |  | Yes | 32223835 |  |
| Oceanobacillus massiliensis | 1465765 | 3.5936 |  |  |  |  |  |  |  |  |  | Yes | 24976893 | 4062624 |
| Ochrobactrum anthropi | 529 | 4.9044 |  |  |  |  |  |  |  |  |  | Yes |  |  |
| Oliverpabstia intestinalis | 2606633 | 3.2334 |  |  |  |  |  |  |  |  |  | Yes | 33319778 | 7738495 |
| Olsenella phocaeensis | 1852385 | 2.2768 |  |  |  |  |  |  |  |  |  | Yes |  |  |
| Olsenella urininfantis | 1871033 | 1.7486 |  |  |  |  |  |  |  |  |  | Yes |  |  |
| Ornithinimicrobium flavum | 1288636 | 3.7129 |  |  |  |  |  |  |  |  |  | Yes | 28945533 |  |
| Oxalobacter formigenes | 847 | 2.4678 |  |  |  |  |  |  |  |  |  | Yes |  |  |
| Paenibacillus sophorae | 1333845 | 5.8070 |  |  |  |  |  |  |  |  |  | Yes |  |  |
| Pandoraea sputorum | 93222 | 5.8498 |  |  |  |  |  |  |  |  |  | Yes | 10758901 |  |
| Pantoea agglomerans | 549 | 4.8638 |  |  |  |  |  |  |  |  |  | Yes | 17442803 | 1933083 |
| Pantoea septica | 472695 | 4.2529 |  |  |  |  |  |  |  |  |  | Yes | 19946052 |  |
| Pantoea sesami | 1881110 | 4.9115 |  |  |  |  |  |  |  |  |  | Yes |  |  |
| Pantoea vagans | 470934 | 4.8240 |  |  |  |  |  |  |  |  |  | Yes | 19620357 |  |
| Parabacteroides distasonis | 823 | 5.1561 |  |  |  |  |  |  |  |  |  | Yes |  |  |
| Paraburkholderia fungorum | 134537 | 8.9510 |  |  |  |  |  |  |  |  |  | Yes |  |  |
| Paraburkholderia insulsa | 1441714 | 9.6209 |  |  |  |  |  |  |  |  |  | Yes |  |  |
| Paraburkholderia phytofirmans | 261302 | 8.3948 |  |  |  |  |  |  |  |  |  | Yes |  |  |
| Paracoccus aeridis | 1966466 | 3.3209 |  |  |  |  |  |  |  |  |  | Yes |  |  |
| Paracoccus chinensis | 525640 | 3.6329 |  |  |  |  |  |  |  |  |  | Yes | 19625438 |  |
| Paracoccus haeundaensis | 225362 | 4.1048 |  |  |  |  |  |  |  |  |  | Yes | 15388731 |  |
| Paracoccus marinus | 288426 | 3.1286 |  |  |  |  |  |  |  |  |  | Yes | 18218935 |  |
| Paracoccus mutanolyticus | 1499308 | 3.5924 |  |  |  |  |  |  |  |  |  | Yes | 27452833 |  |
| Paracoccus salipaludis | 2032623 | 3.6745 |  |  |  |  |  |  |  |  |  | Yes |  |  |
| Paracoccus sanguinis | 1545044 | 3.4940 |  |  |  |  |  |  |  |  |  | Yes |  |  |
| Paracoccus sphaerophysae | 690417 | 3.2617 |  |  |  |  |  |  |  |  |  | Yes | 20400662 |  |
| Paracoccus tibetensis | 336292 | 3.9114 |  |  |  |  |  |  |  |  |  | Yes | 23024140 |  |
| Paracoccus yeei | 147645 | 4.6202 |  |  |  |  |  |  |  |  |  | Yes |  |  |
| Paramuribaculum intestinale | 2094151 | 2.7535 |  |  |  |  |  |  |  |  |  | Yes | 30782206 | 6381624 |
| Paraprevotella clara | 454154 | 4.2105 |  |  |  |  |  |  |  |  |  | Yes |  |  |
| Pasteurella bettyae | 752 | 2.2981 |  |  |  |  |  |  |  |  |  | Yes |  |  |
| Pasteurella multocida | 747 | 2.3203 |  |  |  |  |  |  |  |  |  | Yes |  |  |
| Pectobacterium brasiliense | 180957 | 4.8507 |  |  |  |  |  |  |  |  |  | Yes |  |  |
| Pectobacterium carotovorum | 554 | 4.8248 |  |  |  |  |  |  |  |  |  | Yes | 31343401 |  |
| Pediococcus acidilactici | 1254 | 1.9986 |  |  |  |  |  |  |  |  |  | Yes | 9675696 | 105061 |
| Pediococcus pentosaceus | 1408206 | 1.8304 |  |  |  |  |  |  |  |  |  | Yes |  |  |
| Pedobacter himalayensis | 246796 | 4.6791 |  |  |  |  |  |  |  |  |  | Yes |  |  |
| Peptacetobacter hiranonis | 89152 | 2.5219 |  |  |  |  |  |  |  |  |  | Yes | 32369000 |  |
| Peptococcus niger | 2741 | 1.9685 |  |  |  |  |  |  |  |  |  | Yes |  |  |
| Peptoniphilus coxii | 755172 | 1.7199 |  |  |  |  |  |  |  |  |  | Yes | 22178538 |  |
| Peptoniphilus duerdenii | 507750 | 2.1248 |  |  |  |  |  |  |  |  |  | Yes | 22140159 |  |
| Peptoniphilus harei | 54005 | 1.8360 |  |  |  |  |  |  |  |  |  | Yes |  |  |
| Peptoniphilus rhinitidis | 1175452 | 1.8853 |  |  |  |  |  |  |  |  |  | Yes |  |  |
| Peptostreptococcus anaerobius | 1261 | 2.0950 |  |  |  |  |  |  |  |  |  | Yes |  |  |
| Peptostreptococcus russellii | 215200 | 2.1262 |  |  |  |  |  |  |  |  |  | Yes |  |  |
| Phascolarctobacterium faecium | 33025 | 2.4436 |  |  |  |  |  |  |  |  |  | Yes |  |  |
| Phascolarctobacterium succinatutens | 626940 | 2.0770 |  |  |  |  |  |  |  |  |  | Yes | 22081579 | 3255759 |
| Phenylobacterium zucineum | 284016 | 4.3792 |  |  |  |  |  |  |  |  |  | Yes | 16908113 |  |
| Phocaeicola abscessus | 555313 | 2.5369 |  |  |  |  |  |  |  |  |  | Yes |  |  |
| Phocaeicola vulgatus | 821 | 5.0583 |  |  |  |  |  |  |  |  |  | Yes |  |  |
| Phytobacter diazotrophicus | 395631 | 5.6995 |  |  |  |  |  |  |  |  |  | Yes |  |  |
| Pilimelia anulata | 53371 | 6.9800 |  |  |  |  |  |  |  |  |  | Yes |  |  |
| Planctomonas deserti | 2144185 | 3.8609 |  |  |  |  |  |  |  |  |  | Yes | 30387709 |  |
| Pluralibacter gergoviae | 61647 | 5.5223 |  |  |  |  |  |  |  |  |  | Yes |  |  |
| Porphyromonas asaccharolytica | 28123 | 2.1928 |  |  |  |  | Most likely |  |  |  |  | Yes |  |  |
| Porphyromonas cangingivalis | 36874 | 2.3865 |  |  |  |  |  |  |  |  |  | Yes |  |  |
| Porphyromonas circumdentaria | 29524 | 2.0338 |  |  |  |  |  |  |  |  |  | Yes | 1503973 |  |
| Porphyromonas crevioricanis | 393921 | 2.1069 |  |  |  |  |  |  |  |  |  | Yes |  |  |
| Porphyromonas gulae | 111105 | 2.3561 |  |  |  |  |  |  |  |  |  | Yes |  |  |
| Porphyromonas levii | 1122973 | 2.4417 |  |  |  |  |  |  |  |  |  | Yes |  |  |
| Porphyromonas macacae | 28115 | 2.3246 |  |  |  |  |  |  |  |  |  | Yes |  |  |
| Porphyromonas uenonis | 281920 | 2.2544 |  |  |  |  |  |  |  |  |  | Yes |  |  |
| Prevotella amnii | 419005 | 2.3919 |  |  |  |  |  |  |  |  |  | Yes |  |  |
| Prevotella bergensis | 242750 | 3.2737 |  |  |  |  |  |  |  |  |  | Yes |  |  |
| Prevotella brunnea | 2508867 | 2.5851 |  |  |  |  |  |  |  |  |  | Yes |  |  |
| Prevotella colorans | 1703337 | 2.9367 |  |  |  |  |  |  |  |  |  | Yes | 27150727 |  |
| Prevotella copri | 165179 | 3.6703 |  |  |  |  |  |  |  |  |  | Yes |  |  |
| Prevotella corporis | 1122981 | 2.8002 |  |  |  |  |  |  |  |  |  | Yes |  |  |
| Prevotella dentasini | 1236509 | 2.5466 |  |  |  |  |  |  |  |  |  | Yes |  |  |
| Prevotella disiens | 28130 | 2.8026 |  |  |  |  |  |  |  |  |  | Yes |  |  |
| Prevotella falsenii | 515414 | 2.8002 |  |  |  |  |  |  |  |  |  | Yes |  |  |
| Prevotella hominis | 2518605 | 4.0171 |  |  |  |  |  |  |  |  |  | Yes | 32697186 |  |
| Prevotella ihumii | 1917878 | 3.3234 |  |  |  |  |  |  |  |  |  | Yes |  |  |
| Prevotella jejuni | 1177574 | 3.9289 |  |  |  |  |  |  |  |  |  | Yes |  |  |
| Prevotella pectinovora | 1602169 | 3.1361 |  |  |  |  |  |  |  |  |  | Yes | 2622452 |  |
| Prevotella phocaeensis | 1776388 | 2.9221 |  |  |  |  |  |  |  |  |  | Yes |  |  |
| Prevotella stercorea | 363265 | 3.0973 |  |  |  |  |  |  |  |  |  | Yes |  |  |
| Prevotella timonensis | 386414 | 3.1164 |  |  |  |  |  |  |  |  |  | Yes |  |  |
| Priestia megaterium | 1404 | 5.7126 |  |  |  |  |  |  |  |  |  | Yes | 14602653 | 262321 |
| Propionibacterium cyclohexanicum | 64702 | 2.8090 |  |  |  |  |  |  |  |  |  | Yes |  |  |
| Propionibacterium freudenreichii | 1744 | 2.6309 |  |  |  |  |  |  |  |  |  | Yes |  |  |
| Propionimicrobium lymphophilum | 33012 | 2.0356 |  |  |  |  |  |  |  |  |  | Yes | 28491325 | 5412094 |
| Pseudokineococcus lusitanus | 763993 | 3.8390 |  |  |  |  |  |  |  |  |  | Yes | 21112988 |  |
| Pseudomethylobacillus aquaticus | 2676064 | 2.5502 |  |  |  |  |  |  |  |  |  | Yes |  |  |
| Pseudomonas aeruginosa | 287 | 6.6081 |  |  |  |  |  |  |  |  |  | Yes |  |  |
| Pseudomonas alcaligenes | 43263 | 4.6293 |  |  |  |  |  |  |  |  |  | Yes |  |  |
| Pseudomonas alcaliphila | 101564 | 5.3273 |  |  |  |  |  |  |  |  |  | Yes | 11321079 |  |
| Pseudomonas antarctica | 219572 | 6.3772 |  |  |  |  |  |  |  |  |  | Yes | 15143013 |  |
| Pseudomonas azotoformans | 47878 | 6.6905 |  |  |  |  |  |  |  |  |  | Yes | 10939664 |  |
| Pseudomonas brenneri | 129817 | 5.9930 |  |  |  |  |  |  |  |  |  | Yes | 11446518 |  |
| Pseudomonas canadensis | 915099 | 6.5067 |  |  |  |  |  |  |  |  |  | Yes | 27902304 | 5817194 |
| Pseudomonas chengduensis | 489632 | 5.4131 |  |  |  |  |  |  |  |  |  | Yes | 24021726 |  |
| Pseudomonas costantinii | 168469 | 6.6736 |  |  |  |  |  |  |  |  |  | Yes | 12508856 |  |
| Pseudomonas extremaustralis | 359110 | 6.5812 |  |  |  |  |  |  |  |  |  | Yes | 19688380 |  |
| Pseudomonas fluorescens | 294 | 6.3918 |  |  |  |  |  |  |  |  |  | Yes |  |  |
| Pseudomonas formosensis | 1002526 | 3.1754 |  |  |  |  |  |  |  |  |  | Yes | 23435249 |  |
| Pseudomonas fragi | 296 | 5.0723 |  |  |  |  |  |  |  |  |  | Yes |  |  |
| Pseudomonas indoloxydans | 404407 | 5.1997 |  |  |  |  |  |  |  |  |  | Yes | 18406094 |  |
| Pseudomonas kairouanensis | 2293832 | 6.7023 |  |  |  |  |  |  |  |  |  | Yes | 30910423 |  |
| Pseudomonas koreensis | 198620 | 6.1006 |  |  |  |  |  |  |  |  |  | Yes | 12656147 |  |
| Pseudomonas libanensis | 75588 | 6.2387 |  |  |  |  |  |  |  |  |  | Yes | 10425766 |  |
| Pseudomonas luteola | 47886 | 5.4464 |  |  |  |  |  |  |  |  |  | Yes |  |  |
| Pseudomonas monteilii | 76759 | 5.7883 |  |  |  |  |  |  |  |  |  | Yes |  |  |
| Pseudomonas nabeulensis | 2293833 | 6.7635 |  |  |  |  |  |  |  |  |  | Yes | 30910423 |  |
| Pseudomonas oryzihabitans | 47885 | 5.0411 |  |  |  |  |  |  |  |  |  | Yes |  |  |
| Pseudomonas piscis | 2614538 | 6.9276 |  |  |  |  |  |  |  |  |  | Yes | 32213249 |  |
| Pseudomonas protegens | 380021 | 6.9382 |  |  |  |  |  |  |  |  |  | Yes | 21392918 |  |
| Pseudomonas psychrotolerans | 237610 | 5.2838 |  |  |  |  |  |  |  |  |  | Yes | 15388721 |  |
| Pseudomonas putida | 303 | 6.0784 |  |  |  |  |  |  |  |  |  | Yes | 10939664 |  |
| Pseudomonas salomonii | 191391 | 6.6850 |  |  |  |  |  |  |  |  |  | Yes | 12508870 |  |
| Pseudomonas simiae | 321846 | 6.2045 |  |  |  |  |  |  |  |  |  | Yes |  |  |
| Pseudomonas songnenensis | 1176259 | 4.5533 |  |  |  |  |  |  |  |  |  | Yes |  |  |
| Pseudomonas stutzeri | 316 | 4.3429 |  |  |  |  |  |  |  |  |  | Yes |  |  |
| Pseudomonas stutzeri group | 136846 | 4.3429 |  |  |  |  |  |  |  |  |  | Yes |  |  |
| Pseudomonas syringae | 317 | 6.0344 |  |  |  |  |  |  |  |  |  | Yes |  |  |
| Pseudomonas tolaasii | 29442 | 6.7251 |  |  |  |  |  |  |  |  |  | Yes | 11055932 | 92388 |
| Pseudomonas veronii | 76761 | 7.0805 |  |  |  |  |  |  |  |  |  | Yes | 8863448 |  |
| Pseudomonas zeshuii | 1076620 | 5.3875 |  |  |  |  |  |  |  |  |  | Yes |  |  |
| Pseudonocardia ammonioxydans | 260086 | 7.3615 |  |  |  |  |  |  |  |  |  | Yes | 16514026 |  |
| Pseudorhizobium haltolerans | 1233081 | 4.7017 |  |  |  |  |  |  |  |  |  | Yes |  |  |
| Pseudoxanthomonas spadix | 1045855 | 3.4135 |  |  |  |  |  |  |  |  |  | Yes |  |  |
| Pyrinomonas methylaliphatogenes | 454194 | 3.5089 |  |  |  |  |  |  |  |  |  | Yes | 24048862 |  |
| Ralstonia mannitolilytica | 105219 | 4.8430 |  |  |  |  |  |  |  |  |  | Yes | 29067090 |  |
| Ralstonia pickettii | 329 | 5.2548 |  |  |  |  |  |  |  |  |  | Yes |  |  |
| Raoultella terrigena | 577 | 5.6633 |  |  |  |  |  |  |  |  |  | Yes |  |  |
| Rhodococcus qingshengii | 334542 | 6.7794 |  |  |  |  |  |  |  |  |  | Yes |  |  |
| Rhodopseudomonas palustris | 1076 | 5.3992 |  |  |  |  |  |  |  |  |  | Yes |  |  |
| Romboutsia timonensis | 1776391 | 2.5237 |  |  |  |  |  |  |  |  |  | Yes | 27200178 | 4864248 |
| Roseburia hominis | 301301 | 3.3070 |  |  |  |  |  |  |  |  |  | Yes |  |  |
| Roseburia intestinalis | 166486 | 4.2947 |  |  |  |  |  |  |  |  |  | Yes |  |  |
| Roseomonas aerilata | 452982 | 6.4285 |  |  |  |  |  |  |  |  |  | Yes |  |  |
| Roseomonas cervicalis | 204525 | 5.1010 |  |  |  |  |  |  |  |  |  | Yes |  |  |
| Rothia kristinae | 37923 | 2.3111 |  |  |  |  |  |  |  |  |  | Yes |  |  |
| Rubellimicrobium mesophilum | 1123067 | 4.9218 |  |  |  |  |  |  |  |  |  | Yes |  |  |
| Rubellimicrobium thermophilum | 295419 | 3.1631 |  |  |  |  |  |  |  |  |  | Yes |  |  |
| Rubritepida flocculans | 182403 | 3.8341 |  |  |  |  |  |  |  |  |  | Yes |  |  |
| Rubrobacter xylanophilus | 49319 | 3.1243 |  |  |  |  |  |  |  |  |  | Yes |  |  |
| Rudanella lutea | 451374 | 6.5460 |  |  |  |  |  |  |  |  |  | Yes |  |  |
| Ruminococcus bicirculans | 1160721 | 2.4946 |  |  |  |  |  |  |  |  |  | Yes |  |  |
| Ruminococcus bromii | 40518 | 2.1773 |  |  |  |  |  |  |  |  |  | Yes |  |  |
| Ruminococcus callidus | 40519 | 3.0271 |  |  |  |  |  |  |  |  |  | Yes |  |  |
| Ruminococcus lactaris | 46228 | 2.8336 |  |  |  |  |  |  |  |  |  | Yes |  |  |
| Saccharomonospora glauca | 40990 | 4.5641 |  |  |  |  |  |  |  |  |  | Yes |  |  |
| Saccharopolyspora coralli | 2665642 | 4.8320 |  |  |  |  |  |  |  |  |  | Yes | 32375933 |  |
| Saccharopolyspora dendranthemae | 1181886 | 6.4106 |  |  |  |  |  |  |  |  |  | Yes | 23559043 |  |
| Saccharopolyspora flava | 95161 | 6.2913 |  |  |  |  |  |  |  |  |  | Yes | 11321076 |  |
| Salicibibacter halophilus | 2502791 | 3.7542 |  |  |  |  |  |  |  |  |  | Yes | 31659686 |  |
| Salinicoccus halodurans | 407035 | 2.7569 |  |  |  |  |  |  |  |  |  | Yes | 18599690 |  |
| Salinicoccus kekensis | 714307 | 2.6104 |  |  |  |  |  |  |  |  |  | Yes | 20449768 |  |
| Salmonella enterica | 28901 | 4.7986 |  |  |  |  |  |  |  |  |  | Yes |  |  |
| Sanguibacter massiliensis | 1973217 | 3.0734 |  |  |  |  |  |  |  |  |  | Yes | 29922471 | 6004731 |
| Secundilactobacillus oryzae | 1202668 | 1.8503 |  |  |  |  |  |  |  |  |  | Yes | 23378109 |  |
| Selenomonas bovis | 416586 | 2.1601 |  |  |  |  |  |  |  |  |  | Yes |  |  |
| Selenomonas ruminantium | 971 | 2.8216 |  |  |  |  |  |  |  |  |  | Yes |  |  |
| Senegalimassilia anaerobia | 1473216 | 2.3811 |  |  |  |  |  |  |  |  |  | Yes |  |  |
| Serinicoccus hydrothermalis | 1758689 | 3.5974 |  |  |  |  |  |  |  |  |  | Yes | 32375934 |  |
| Serinicoccus marinus | 247333 | 3.4084 |  |  |  |  |  |  |  |  |  | Yes | 15388714 |  |
| Serinicoccus profundi | 1078471 | 3.4548 |  |  |  |  |  |  |  |  |  | Yes |  |  |
| Serratia ficaria | 61651 | 5.2313 |  |  |  |  |  |  |  |  |  | Yes |  |  |
| Serratia grimesii | 82995 | 5.1061 |  |  |  |  |  |  |  |  |  | Yes |  |  |
| Serratia marcescens | 615 | 5.2101 |  |  |  |  |  |  |  |  |  | Yes |  |  |
| Serratia nematodiphila | 458197 | 5.2145 |  |  |  |  |  |  |  |  |  | Yes |  |  |
| Serratia plymuthica | 82996 | 5.4494 |  |  |  |  |  |  |  |  |  | Yes |  |  |
| Serratia surfactantfaciens | 2741499 | 5.1734 |  |  |  |  |  |  |  |  |  | Yes |  |  |
| Serratia symbiotica | 138074 | 2.4368 |  |  |  |  |  |  |  |  |  | Yes |  |  |
| Serratia ureilytica | 300181 | 5.1456 |  |  |  |  |  |  |  |  |  | Yes |  |  |
| Shewanella putrefaciens | 1305841 | 4.6592 |  |  |  |  |  |  |  |  |  | Yes |  |  |
| Shigella boydii | 621 | 4.6784 |  |  |  |  |  |  |  |  |  | Yes |  | 4027345 |
| Shigella dysenteriae | 622 | 4.6136 |  |  |  |  |  |  |  |  |  | Yes | 12147489 | 124020 |
| Shigella flexneri | 623 | 4.5847 |  |  |  |  |  |  |  |  |  | Yes |  |  |
| Shigella sonnei | 624 | 4.6676 |  |  |  |  |  |  |  |  |  | Yes | 26004640 | 4443274 |
| Simonsiella muelleri | 72 | 2.4212 |  |  |  |  |  |  |  |  |  | Yes |  |  |
| Skermanella aerolata | 393310 | 8.5316 |  |  |  |  |  |  |  |  |  | Yes | 30364926 | 6254487 |
| Slackia isoflavoniconvertens | 572010 | 2.3187 |  |  |  |  |  |  |  |  |  | Yes |  |  |
| Sneathia sanguinegens | 40543 | 1.2910 |  |  |  |  |  |  |  |  |  | Yes | 7548563 |  |
| Snodgrassella alvi | 1196083 | 2.4861 |  |  |  |  |  |  |  |  |  | Yes | 23041637 |  |
| Sodaliphilus pleomorphus | 2606626 | 3.3120 |  |  |  |  |  |  |  |  |  | Yes | 33319778 | 7738495 |
| Solimonas variicoloris | 254408 | 4.1195 |  |  |  |  |  |  |  |  |  | Yes |  |  |
| Sphaerisporangium cinnabarinum | 47482 | 4.3875 |  |  |  |  |  |  |  |  |  | Yes |  |  |
| Sphaerotilus natans | 34103 | 4.6301 |  |  |  |  |  |  |  |  |  | Yes | 20495027 |  |
| Sphingobium cloacae | 120107 | 4.4191 |  |  |  |  |  |  |  |  |  | Yes |  |  |
| Sphingobium limneticum | 1007511 | 5.1532 |  |  |  |  |  |  |  |  |  | Yes | 22561591 |  |
| Sphingobium yanoikuyae | 13690 | 5.4596 |  |  |  |  |  |  |  |  |  | Yes |  |  |
| Sphingomonas aquatilis | 93063 | 3.8410 |  |  |  |  |  |  |  |  |  | Yes | 11491350 |  |
| Sphingomonas astaxanthinifaciens | 407019 | 2.5330 |  |  |  |  |  |  |  |  |  | Yes |  |  |
| Sphingomonas ginsenosidimutans | 862134 | 3.7327 |  |  |  |  |  |  |  |  |  | Yes | 21221931 |  |
| Sphingomonas koreensis | 93064 | 4.8302 |  |  |  |  |  |  |  |  |  | Yes | 11491350 |  |
| Sphingomonas melonis | 152682 | 3.9963 |  |  |  |  |  |  |  |  |  | Yes |  |  |
| Sphingomonas parapaucimobilis | 28213 | 3.9945 |  |  |  |  |  |  |  |  |  | Yes | 2111872 |  |
| Sphingomonas sanguinis | 33051 | 4.1499 |  |  |  |  |  |  |  |  |  | Yes | 17551055 |  |
| Sphingopyxis alaskensis | 117207 | 3.5754 |  |  |  |  |  |  |  |  |  | Yes | 11211276 |  |
| Staphylococcus argenteus | 985002 | 2.7935 |  |  |  |  |  |  |  |  |  | Yes |  |  |
| Staphylococcus aureus | 1280 | 2.8369 |  |  |  |  |  |  |  |  |  | Yes |  |  |
| Staphylococcus capitis | 29388 | 2.4850 |  |  |  |  |  |  |  |  |  | Yes |  |  |
| Staphylococcus caprae | 29380 | 2.6296 |  |  |  |  |  |  |  |  |  | Yes | 30613447 | 6314791 |
| Staphylococcus epidermidis | 1282 | 2.5166 |  |  |  |  |  |  |  |  |  | Yes |  |  |
| Staphylococcus haemolyticus | 1283 | 2.5086 |  |  |  |  |  |  |  |  |  | Yes |  |  |
| Staphylococcus hominis | 1290 | 2.2500 |  |  |  |  |  |  |  |  |  | Yes |  |  |
| Staphylococcus pasteuri | 45972 | 2.5438 |  |  |  |  |  |  |  |  |  | Yes | 8098615 |  |
| Staphylococcus pettenkoferi | 170573 | 2.4723 |  |  |  |  |  |  |  |  |  | Yes |  |  |
| Staphylococcus pseudintermedius | 283734 | 2.6574 |  |  |  |  |  |  |  |  |  | Yes |  |  |
| Staphylococcus schweitzeri | 1654388 | 2.7547 |  |  |  |  |  |  |  |  |  | Yes |  |  |
| Staphylococcus warneri | 1292 | 2.5523 |  |  |  |  |  |  |  |  |  | Yes |  |  |
| Staphylococcus xylosus | 1288 | 2.8359 |  |  |  |  |  |  |  |  |  | Yes |  |  |
| Stenotrophomonas bentonitica | 1450134 | 4.3869 |  |  |  |  |  |  |  |  |  | Yes | 28820086 | 5817250 |
| Stenotrophomonas maltophilia | 40324 | 4.6365 |  |  |  |  |  |  |  |  |  | Yes |  |  |
| Stenotrophomonas pavanii | 487698 | 4.4031 |  |  |  |  |  |  |  |  |  | Yes |  |  |
| Stenotrophomonas rhizophila | 216778 | 4.2366 |  |  |  |  |  |  |  |  |  | Yes | 12508851 |  |
| Streptobacillus moniliformis | 519441 | 1.6577 |  |  |  |  |  |  |  |  |  | Yes |  |  |
| Streptococcus alactolyticus | 29389 | 1.6982 |  |  |  |  |  |  |  |  |  | Yes | 27525136 | 4972922 |
| Streptococcus caballi | 439220 | 2.1220 |  |  |  |  |  |  |  |  |  | Yes |  |  |
| Streptococcus castoreus | 254786 | 1.8835 |  |  |  |  |  |  |  |  |  | Yes |  |  |
| Streptococcus downei | 1317 | 2.2358 |  |  |  |  |  |  |  |  |  | Yes |  |  |
| Streptococcus dysgalactiae | 1334 | 2.1640 |  |  |  |  |  |  |  |  |  | Yes |  |  |
| Streptococcus equi | 1336 | 2.1299 |  |  |  |  |  |  |  |  |  | Yes |  |  |
| Streptococcus equinus | 1335 | 1.8757 |  |  |  |  |  |  |  |  |  | Yes |  |  |
| Streptococcus ferus | 1123303 | 1.8598 |  |  |  |  |  |  |  |  |  | Yes |  |  |
| Streptococcus gallolyticus | 981539 | 2.3114 |  |  |  |  |  |  |  |  |  | Yes |  |  |
| Streptococcus hyointestinalis | 1337 | 2.2171 |  |  |  |  |  |  |  |  |  | Yes |  |  |
| Streptococcus lutetiensis | 1076934 | 1.8490 |  |  |  |  |  |  |  |  |  | Yes |  |  |
| Streptococcus macedonicus | 59310 | 2.1637 |  |  |  |  |  |  |  |  |  | Yes |  |  |
| Streptococcus orisasini | 1080071 | 2.2992 |  |  |  |  |  |  |  |  |  | Yes | 23291897 |  |
| Streptococcus parauberis | 1348 | 2.0843 |  |  |  |  |  |  |  |  |  | Yes | 24092782 | 3790086 |
| Streptococcus pasteurianus | 197614 | 2.1598 |  |  |  |  |  |  |  |  |  | Yes | 12148636 |  |
| Streptococcus pluranimalium | 82348 | 2.2460 |  |  |  |  |  |  |  |  |  | Yes |  |  |
| Streptococcus suis | 1307 | 2.0972 |  |  |  |  |  |  |  |  |  | Yes |  |  |
| Streptococcus thermophilus | 1308 | 1.8320 |  |  |  |  |  |  |  |  |  | Yes |  |  |
| Streptococcus timonensis | 1852387 | 1.9253 |  |  |  |  |  |  |  |  |  | Yes |  |  |
| Streptococcus troglodytae | 1111760 | 2.0979 |  |  |  |  |  |  |  |  |  | Yes | 27921343 |  |
| Streptococcus viridans | 78535 | 1.9976 |  |  |  |  |  |  |  |  |  | Yes |  |  |
| Streptomyces avermitilis | 33903 | 9.9368 |  |  |  |  |  |  |  |  |  | Yes | 12508861 |  |
| Sutterella wadsworthensis | 40545 | 2.8758 |  |  |  |  |  |  |  |  |  | Yes |  |  |
| Tepidimonas fonticaldi | 1101373 | 2.8754 |  |  |  |  |  |  |  |  |  | Yes | 22984136 |  |
| Tepidimonas taiwanensis | 307486 | 2.8635 |  |  |  |  |  |  |  |  |  | Yes | 16215773 |  |
| Tepidiphilus succinatimandens | 224436 | 2.3696 |  |  |  |  |  |  |  |  |  | Yes | 15143002 |  |
| Tepidiphilus thermophilus | 876478 | 2.2598 |  |  |  |  |  |  |  |  |  | Yes | 24048864 |  |
| Tessaracoccus massiliensis | 1522311 | 3.2117 |  |  |  |  |  |  |  |  |  | Yes |  |  |
| Tessaracoccus rhinocerotis | 1689449 | 3.5476 |  |  |  |  |  |  |  |  |  | Yes |  |  |
| Thermobacillus composti | 377615 | 4.3555 |  |  |  |  |  |  |  |  |  | Yes | 17625178 |  |
| Thermus parvatiensis | 456163 | 2.0264 |  |  |  |  |  |  |  |  |  | Yes | 26543260 |  |
| Thermus scotoductus | 37636 | 2.3110 |  |  |  |  |  |  |  |  |  | Yes |  |  |
| Thermus thermophilus | 274 | 2.1662 |  |  |  |  |  |  |  |  |  | Yes |  |  |
| Thiobacillus denitrificans | 36861 | 3.1893 |  |  |  |  |  |  |  |  |  | Yes |  |  |
| Tissierella praeacuta | 43131 | 3.1653 |  |  |  |  |  |  |  |  |  | Yes |  |  |
| Treponema berlinense | 225004 | 2.2259 |  |  |  |  |  |  |  |  |  | Yes | 16014500 |  |
| Treponema porcinum | 261392 | 1.9711 |  |  |  |  |  |  |  |  |  | Yes | 16014500 |  |
| Trueperella bernardiae | 59561 | 2.0468 |  |  |  |  |  |  |  |  |  | Yes |  |  |
| Turicella otitidis | 883169 | 2.1444 |  |  |  |  |  |  |  |  |  | Yes |  |  |
| Undibacterium pigrum | 401470 | 6.4071 |  |  |  |  |  |  |  |  |  | Yes | 17625185 |  |
| Varibaculum cambriense | 184870 | 2.0970 |  |  |  |  |  |  |  |  |  | Yes |  |  |
| Varibaculum massiliense | 1852372 | 2.2834 |  |  |  |  |  |  |  |  |  | Yes |  |  |
| Varibaculum vaginae | 2364797 | 2.0150 |  |  |  |  |  |  |  |  |  | Yes | 31367386 | 6656688 |
| Variovorax paradoxus | 34073 | 7.0117 |  |  |  |  |  |  |  |  |  | Yes |  |  |
| Veillonella caviae | 248316 | 1.9640 |  |  |  |  |  |  |  |  |  | Yes |  |  |
| Veillonella rodentium | 248315 | 2.0413 |  |  |  |  |  |  |  |  |  | Yes |  |  |
| Vibrio cholerae | 666 | 4.0201 |  |  |  |  |  |  |  |  |  | Yes |  |  |
| Vulcaniibacterium thermophilum | 1169913 | 3.0756 |  |  |  |  |  |  |  |  |  | Yes | 23864384 |  |
| Weissella cibaria | 137591 | 2.4556 |  |  |  |  |  |  |  |  |  | Yes |  |  |
| Weissella confusa | 1583 | 2.2585 |  |  |  |  |  |  |  |  |  | Yes |  |  |
| Weissella viridescens | 1629 | 1.5703 |  |  |  |  |  |  |  |  |  | Yes | 16503360 |  |
| Xanthomonas campestris | 190485 | 5.0054 |  |  |  |  |  |  |  |  |  | Yes |  |  |
| Xanthomonas citri | 346 | 5.2633 |  |  |  |  |  |  |  |  |  | Yes | 20569374 |  |
| Yaniella halotolerans | 225453 | 2.7755 |  |  |  |  |  |  |  |  |  | Yes |  |  |
| Yersinia pestis | 632 | 4.5963 |  |  |  |  |  |  |  |  |  | Yes |  |  |
| Yersinia pseudotuberculosis | 633 | 4.7209 |  |  |  |  |  |  |  |  |  | Yes |  |  |
| Yersinia ruckeri | 29486 | 3.7577 |  |  |  |  |  |  |  |  |  | Yes |  |  |
| Yimella lutea | 587872 | 3.5331 |  |  |  |  |  |  |  |  |  | Yes | 19656924 |  |
| Zymomonas mobilis | 542 | 2.0669 |  |  |  |  |  |  |  |  |  | Yes |  |  |
| Abiotrophia_u_s | 46123 | 1.8908 |  |  |  |  |  |  |  |  | Yes |  |  |  |
| Acetomicrobium_u_s | 49894 | 2.0210 |  |  |  |  |  |  |  |  | Yes |  |  |  |
| Acholeplasma_u_s | 2147 | 1.0404 |  |  |  |  |  |  |  |  | Yes |  |  |  |
| Achromobacter_u_s | 222 | 6.2908 |  |  |  |  |  |  |  |  | Yes |  |  |  |
| Acidaminococcus_u_s | 904 | 2.3371 |  |  |  |  |  |  |  |  | Yes |  |  |  |
| Acidimicrobiaceae_u_s | 84994 | 2.0825 |  |  |  |  |  |  |  |  | Yes |  |  |  |
| Acidipropionibacterium_u_s | 1912215 | 3.1717 |  |  |  |  |  |  |  |  | Yes |  |  |  |
| Acidithiobacillales_u_s | 225057 | 3.0154 |  |  |  |  |  |  |  |  | Yes |  |  |  |
| Acidovorax_u_s | 12916 | 5.0149 |  |  |  |  |  |  |  |  | Yes |  |  |  |
| Acinetobacter sp. 479375 | 1310601 | 3.6702 |  |  |  |  |  |  |  |  | Yes |  |  |  |
| Acinetobacter sp. NIPH 284 | 1217704 | 3.6702 |  |  |  |  |  |  |  |  | Yes |  |  |  |
| Acinetobacter sp. WC-141 | 903915 | 3.4025 |  |  |  |  |  |  |  |  | Yes |  |  |  |
| Acinetobacter_u_s | 469 | 3.6702 |  |  |  |  |  |  |  |  | Yes |  |  |  |
| Actibacterium_u_s | 1433986 | 2.7086 |  |  |  |  |  |  |  |  | Yes |  |  |  |
| Actinobacillus_u_s | 713 | 2.3292 |  |  |  |  |  |  |  |  | Yes |  |  |  |
| actinobacterium SCGC AAA023-D18 | 932037 | 0.7533 |  |  |  |  |  |  |  |  | Yes |  |  |  |
| actinobacterium SCGC AAA027-J17 | 932040 | 0.9666 |  |  |  |  |  |  |  |  | Yes |  |  |  |
| Actinobaculum_u_s | 76833 | 2.5675 |  |  |  |  |  |  |  |  | Yes |  |  |  |
| Actinokineospora_u_s | 39845 | 6.4395 |  |  |  |  |  |  |  |  | Yes |  |  |  |
| Actinomadura_u_s | 1988 | 9.0635 |  |  |  |  |  |  |  |  | Yes |  |  |  |
| Actinomyces sp. oral taxon 170 | 712117 | 2.4039 |  |  |  |  |  |  |  |  | Yes |  |  |  |
| Actinomyces sp. oral taxon 171 | 706438 | 2.4039 |  |  |  |  |  |  |  |  | Yes |  |  |  |
| Actinomyces sp. oral taxon 175 | 712119 | 2.4039 |  |  |  |  |  |  |  |  | Yes |  |  |  |
| Actinomyces sp. oral taxon 178 | 710238 | 2.4039 |  |  |  |  |  |  |  |  | Yes |  |  |  |
| Actinomyces sp. oral taxon 180 | 651609 | 2.4039 |  |  |  |  |  |  |  |  | Yes |  |  |  |
| Actinomyces sp. oral taxon 448 | 712124 | 2.4039 |  |  |  |  |  |  |  |  | Yes |  |  |  |
| Actinomyces sp. oral taxon 848 | 649739 | 2.4039 |  |  |  |  |  |  |  |  | Yes |  |  |  |
| Actinomyces sp. oral taxon 849 | 653385 | 2.4039 |  |  |  |  |  |  |  |  | Yes |  |  |  |
| Actinomyces sp. oral taxon 877 | 1227263 | 2.4039 |  |  |  |  |  |  |  |  | Yes |  |  |  |
| Actinomyces_u_s | 1654 | 2.2808 |  |  |  |  |  |  |  |  | Yes |  |  |  |
| Actinoplanes_u_s | 1865 | 9.2400 |  |  |  |  |  |  |  |  | Yes |  |  |  |
| Actinotalea_u_s | 458839 | 4.0274 |  |  |  |  |  |  |  |  | Yes |  |  |  |
| Actinotignum_u_s | 1653174 | 2.0749 |  |  |  |  |  |  |  |  | Yes |  |  |  |
| Adlercreutzia_u_s | 447020 | 2.3670 |  |  |  |  |  |  |  |  | Yes |  |  |  |
| Aequorivita_u_s | 153265 | 3.1609 |  |  |  |  |  |  |  |  | Yes |  |  |  |
| Aerococcus_u_s | 1375 | 2.1064 |  |  |  |  |  |  |  |  | Yes |  |  |  |
| Aeromonas_u_s | 642 | 4.6156 |  |  |  |  |  |  |  |  | Yes |  |  |  |
| Afipia_u_s | 1033 | 4.7042 |  |  |  |  |  |  |  |  | Yes |  |  |  |
| Aggregatibacter sp. oral taxon 458 | 712148 | 2.0166 |  |  |  |  |  |  |  |  | Yes |  |  |  |
| Aggregatibacter_u_s | 416916 | 2.0166 |  |  |  |  |  |  |  |  | Yes |  |  |  |
| Agrobacterium_u_s | 357 | 5.5333 |  |  |  |  |  |  |  |  | Yes |  |  |  |
| Agrococcus_u_s | 46352 | 3.0210 |  |  |  |  |  |  |  |  | Yes |  |  |  |
| Alcaligenes_u_s | 507 | 4.2737 |  |  |  |  |  |  |  |  | Yes |  |  |  |
| Alishewanella_u_s |  |  |  |  |  |  |  |  |  |  | Yes |  |  |  |
| Alistipes sp. HGB5 | 908612 | 2.5016 |  |  |  |  |  |  |  |  | Yes |  |  |  |
| Alistipes_u_s | 239759 | 2.5016 |  |  |  |  |  |  |  |  | Yes |  |  |  |
| Alloscardovia_u_s | 419014 | 1.9610 |  |  |  |  |  |  |  |  | Yes |  |  |  |
| alpha proteobacterium LLX12A | 1229484 | 5.9611 |  |  |  |  |  |  |  |  | Yes |  |  |  |
| alpha proteobacterium SCGC AAA023-L09 | 938615 | 0.7749 |  |  |  |  |  |  |  |  | Yes |  |  |  |
| alpha proteobacterium SCGC AAA028-D10 | 938641 | 0.9251 |  |  |  |  |  |  |  |  | Yes |  |  |  |
| Aminobacterium_u_s | 81466 | 1.6666 |  |  |  |  |  |  |  |  | Yes |  |  |  |
| Amycolatopsis_u_s | 1813 | 9.3329 |  |  |  |  |  |  |  |  | Yes |  |  |  |
| Anaerobutyricum hallii | 39488 | 3.4209 |  |  |  |  |  |  |  |  | Yes |  |  |  |
| Anaerococcus prevotii | 33034 | 2.0018 |  |  |  |  |  |  |  |  | Yes |  |  |  |
| Anaerococcus_u_s | 165779 | 1.7950 |  |  |  |  |  |  |  |  | Yes |  |  |  |
| Anaeromyxobacter_u_s | 161492 | 5.1698 |  |  |  |  |  |  |  |  | Yes |  |  |  |
| Anaerostipes_u_s | 207244 | 2.7295 |  |  |  |  |  |  |  |  | Yes |  |  |  |
| Anoxybacillus_u_s | 150247 | 3.5411 |  |  |  |  |  |  |  |  | Yes |  |  |  |
| Aquabacterium sp. NJ1 | 1538295 | 4.2041 |  |  |  |  |  |  |  |  | Yes |  |  |  |
| Aquificaceae_u_s | 64898 | 1.6014 |  |  |  |  |  |  |  |  | Yes |  |  |  |
| Arcanobacterium_u_s | 28263 | 1.9412 |  |  |  |  |  |  |  |  | Yes |  |  |  |
| Arenimonas_u_s | 490567 | 2.6810 |  |  |  |  |  |  |  |  | Yes |  |  |  |
| Arsenicicoccus_u_s | 267408 | 3.8366 |  |  |  |  |  |  |  |  | Yes |  |  |  |
| Arthrobacter_u_s | 1663 | 4.3992 |  |  |  |  |  |  |  |  | Yes |  |  |  |
| Asaia_u_s | 91914 | 3.7430 |  |  |  |  |  |  |  |  | Yes |  |  |  |
| Atopobium sp. BS2 | 936550 | 1.9724 |  |  |  |  |  |  |  |  | Yes |  |  |  |
| Atopobium sp. ICM42b | 1190620 | 1.9724 |  |  |  |  |  |  |  |  | Yes |  |  |  |
| Atopobium sp. oral taxon 199 | 712156 | 1.9724 |  |  |  |  |  |  |  |  | Yes |  |  |  |
| Atopobium sp. oral taxon 810 | 712158 | 1.9724 |  |  |  |  |  |  |  |  | Yes |  |  |  |
| Atopobium_u_s | 1380 | 1.4721 |  |  |  |  |  |  |  |  | Yes |  |  |  |
| Aureimonas_u_s | 414371 | 4.8451 |  |  |  |  |  |  |  |  | Yes |  |  |  |
| Azoarcus_u_s | 12960 | 4.8253 |  |  |  |  |  |  |  |  | Yes |  |  |  |
| Azonexaceae_u_s | 2008795 | 3.6236 |  |  |  |  |  |  |  |  | Yes |  |  |  |
| Azospirillum_u_s | 191 | 6.9829 |  |  |  |  |  |  |  |  | Yes |  |  |  |
| Bacillus sp. EGD-AK10 | 1386080 | 4.9436 |  |  |  |  |  |  |  |  | Yes |  |  |  |
| Bacillus_u_s | 1386 | 4.9436 |  |  |  |  |  |  |  |  | Yes |  |  |  |
| Bacteria_u_s | 2 |  |  |  |  |  |  |  |  |  | Yes |  |  |  |
| Bacteroides sp. 3_1_40A | 469593 | 4.5699 |  |  |  |  |  |  |  |  | Yes |  |  |  |
| Bacteroides sp. 4_1_36 | 457393 | 4.5699 |  |  |  |  |  |  |  |  | Yes |  |  |  |
| Bacteroides sp. 4_3_47FAA | 457394 | 4.5699 |  |  |  |  |  |  |  |  | Yes |  |  |  |
| Bacteroides sp. 9_1_42FAA | 457395 | 4.5699 |  |  |  |  |  |  |  |  | Yes |  |  |  |
| Bacteroides sp. D2 | 556259 | 4.4149 |  |  |  |  |  |  |  |  | Yes |  |  |  |
| Bacteroides_u_s | 816 | 4.5699 |  |  |  |  |  |  |  |  | Yes |  |  |  |
| Bacteroidetes bacterium oral taxon 272 | 651591 | 2.5027 |  |  |  |  |  |  |  |  | Yes |  |  |  |
| Belnapia sp. F-4-1 | 1545443 | 5.9623 |  |  |  |  |  |  |  |  | Yes |  |  |  |
| Bifidobacterium sp. 12_1_47BFAA | 469594 | 2.3955 |  |  |  |  |  |  |  |  | Yes |  |  |  |
| Bifidobacterium sp. MSTE12 | 1161409 | 1.9665 |  |  |  |  |  |  |  |  | Yes |  |  |  |
| Bifidobacterium_u_s | 1678 | 1.9988 |  |  |  |  |  |  |  |  | Yes |  |  |  |
| Bilophila_u_s | 35832 | 2.6157 |  |  |  |  |  |  |  |  | Yes |  |  |  |
| Blastocatellia_u_s | 1562566 | 4.3963 |  |  |  |  |  |  |  |  | Yes |  |  |  |
| Blastococcus_u_s | 38501 | 4.3379 |  |  |  |  |  |  |  |  | Yes |  |  |  |
| Blastomonas_u_s | 150203 | 4.1059 |  |  |  |  |  |  |  |  | Yes |  |  |  |
| Blattabacterium_u_s | 34098 | 0.6350 |  |  |  |  |  |  |  |  | Yes |  |  |  |
| Blautia_u_s | 572511 | 3.3630 |  |  |  |  |  |  |  |  | Yes |  |  |  |
| Bordetella_u_s | 517 | 4.5374 |  |  |  |  |  |  |  |  | Yes |  |  |  |
| Bosea_u_s | 85413 | 5.6674 |  |  |  |  |  |  |  |  | Yes |  |  |  |
| Brachyspira_u_s | 29521 | 2.4584 |  |  |  |  |  |  |  |  | Yes |  |  |  |
| Bradyrhizobium sp. DFCI-1 | 1230476 | 8.0456 |  |  |  |  |  |  |  |  | Yes |  |  |  |
| Bradyrhizobium_u_s | 374 | 8.0456 |  |  |  |  |  |  |  |  | Yes |  |  |  |
| Brevibacillus_u_s | 55080 | 5.9365 |  |  |  |  |  |  |  |  | Yes |  |  |  |
| Brevibacterium_u_s | 1696 | 3.7816 |  |  |  |  |  |  |  |  | Yes |  |  |  |
| Brevundimonas_u_s | 41275 | 3.0988 |  |  |  |  |  |  |  |  | Yes |  |  |  |
| Brochothrix_u_s | 2755 | 2.4592 |  |  |  |  |  |  |  |  | Yes |  |  |  |
| Burkholderia_u_s | 32008 | 7.5699 |  |  |  |  |  |  |  |  | Yes |  |  |  |
| butyrate-producing bacterium SS3/4 | 245014 | 3.6010 |  |  |  |  |  |  |  |  | Yes |  |  |  |
| Butyricicoccus_u_s | 580596 | 2.2702 |  |  |  |  |  |  |  |  | Yes |  |  |  |
| Calothrix sp. PCC 7103 | 32057 | 7.5123 |  |  |  |  |  |  |  |  | Yes |  |  |  |
| Campylobacter sp. 10_1_50 | 665939 | 1.6705 |  |  |  |  |  |  |  |  | Yes |  |  |  |
| Campylobacter sp. FOBRC14 | 936554 | 1.6705 |  |  |  |  |  |  |  |  | Yes |  |  |  |
| Campylobacter_u_s | 194 | 1.6705 |  |  |  |  |  |  |  |  | Yes |  |  |  |
| Candidatus Absconditabacteria_u_s | 221235 | 1.0592 |  |  |  |  |  |  |  |  | Yes |  |  |  |
| Candidatus Arthromitus_u_s | 49082 | 1.5298 |  |  |  |  |  |  |  |  | Yes |  |  |  |
| Candidatus Saccharibacteria_u_s | 95818 | 0.7033 |  |  |  |  |  |  |  |  | Yes |  |  |  |
| Capnocytophaga sp. CM59 | 936370 | 2.6693 |  |  |  |  |  |  |  |  | Yes | No | 22057871 | 3255620 |
| Capnocytophaga sp. oral taxon 324 | 712211 | 2.6693 |  |  |  |  |  |  |  |  | Yes | No |  |  |
| Capnocytophaga sp. oral taxon 326 | 712212 | 2.6693 |  |  |  |  |  |  |  |  | Yes | No |  |  |
| Capnocytophaga sp. oral taxon 329 | 706435 | 2.6693 |  |  |  |  |  |  |  |  | Yes | No |  |  |
| Capnocytophaga sp. oral taxon 332 | 712213 | 2.6693 |  |  |  |  |  |  |  |  | Yes | No |  |  |
| Capnocytophaga sp. oral taxon 335 | 712215 | 2.6693 |  |  |  |  |  |  |  |  | Yes | No |  |  |
| Capnocytophaga sp. oral taxon 336 | 712216 | 2.6693 |  |  |  |  |  |  |  |  | Yes | No |  |  |
| Capnocytophaga sp. oral taxon 338 | 710239 | 2.6693 |  |  |  |  |  |  |  |  | Yes | No |  |  |
| Capnocytophaga sp. oral taxon 380 | 712217 | 2.6693 |  |  |  |  |  |  |  |  | Yes | No |  |  |
| Capnocytophaga sp. oral taxon 412 | 712218 | 2.6693 |  |  |  |  |  |  |  |  | Yes | No |  |  |
| Capnocytophaga sp. oral taxon 863 | 1227265 | 2.6693 |  |  |  |  |  |  |  |  | Yes | No |  |  |
| Capnocytophaga_u_s | 1016 | 2.6693 |  |  |  |  |  |  |  |  | Yes |  |  |  |
| Cardiobacterium_u_s | 2717 | 3.8633 |  |  |  |  |  |  |  |  | Yes |  |  |  |
| Carnobacterium_u_s | 2747 | 2.0477 |  |  |  |  |  |  |  |  | Yes |  |  |  |
| Caulobacter_u_s | 75 | 4.8803 |  |  |  |  |  |  |  |  | Yes |  |  |  |
| Cellulosimicrobium_u_s | 157920 | 4.3220 |  |  |  |  |  |  |  |  | Yes |  |  |  |
| Christensenella_u_s | 990721 | 2.7244 |  |  |  |  |  |  |  |  | Yes |  |  |  |
| Chromobacterium_u_s | 535 | 4.8072 |  |  |  |  |  |  |  |  | Yes |  |  |  |
| Chroococcidiopsis_u_s | 54298 | 6.6743 |  |  |  |  |  |  |  |  | Yes |  |  |  |
| Chryseobacterium_u_s | 59732 | 4.4925 |  |  |  |  |  |  |  |  | Yes |  |  |  |
| Chrysiogenaceae_u_s | 189770 | 2.8768 |  |  |  |  |  |  |  |  | Yes |  |  |  |
| Citrobacter_u_s | 544 | 5.0459 |  |  |  |  |  |  |  |  | Yes |  |  |  |
| Clavibacter_u_s | 1573 | 3.1119 |  |  |  |  |  |  |  |  | Yes |  |  |  |
| Clostridiales_u_s | 186802 | 2.6908 |  |  |  |  |  |  |  |  | Yes |  |  |  |
| Clostridium_u_s | 1485 | 2.9606 |  |  |  |  |  |  |  |  | Yes |  |  |  |
| Cohnella_u_s | 329857 | 6.4263 |  |  |  |  |  |  |  |  | Yes |  |  |  |
| Collinsella_u_s | 102106 | 2.0388 |  |  |  |  |  |  |  |  | Yes |  |  |  |
| Comamonas_u_s | 283 | 3.8396 |  |  |  |  |  |  |  |  | Yes |  |  |  |
| Commensalibacter_u_s | 1079922 | 2.0101 |  |  |  |  |  |  |  |  | Yes |  |  |  |
| Conchiformibius_u_s | 334107 | 2.1889 |  |  |  |  |  |  |  |  | Yes |  |  |  |
| Coprobacillus sp. 8_2_54BFAA | 469597 | 2.8270 |  |  |  |  |  |  |  |  | Yes |  |  |  |
| Coprococcus_u_s | 33042 | 2.8235 |  |  |  |  |  |  |  |  | Yes |  |  |  |
| Coprothermobacter_u_s | 68335 | 1.6081 |  |  |  |  |  |  |  |  | Yes |  |  |  |
| Corynebacterium sp. KPL1824 | 1203561 | 2.4825 |  |  |  |  |  |  |  |  | Yes |  |  |  |
| Corynebacterium_u_s | 1716 | 2.4734 |  |  |  |  |  |  |  |  | Yes |  |  |  |
| Cupriavidus_u_s | 106589 | 6.7662 |  |  |  |  |  |  |  |  | Yes |  |  |  |
| Curtobacterium_u_s | 2034 | 3.7106 |  |  |  |  |  |  |  |  | Yes |  |  |  |
| Dehalococcoides_u_s | 61434 | 1.1909 |  |  |  |  |  |  |  |  | Yes |  |  |  |
| Deinococcus_u_s | 1298 | 3.5543 |  |  |  |  |  |  |  |  | Yes |  |  |  |
| Delftia_u_s | 80865 | 6.4761 |  |  |  |  |  |  |  |  | Yes |  |  |  |
| Dermabacter_u_s | 36739 | 2.2566 |  |  |  |  |  |  |  |  | Yes |  |  |  |
| Dermacoccus sp. Ellin185 | 188626 | 3.0750 |  |  |  |  |  |  |  |  | Yes |  |  |  |
| Dermacoccus_u_s | 57495 | 3.0750 |  |  |  |  |  |  |  |  | Yes |  |  |  |
| Desulfobulbus sp. Dsb1 | 1292026 | 2.6601 |  |  |  |  |  |  |  |  | Yes |  |  |  |
| Desulfobulbus sp. Dsb5 | 1292025 | 2.7042 |  |  |  |  |  |  |  |  | Yes |  |  |  |
| Desulfobulbus_u_s | 893 | 2.7042 |  |  |  |  |  |  |  |  | Yes |  |  |  |
| Desulfomicrobium_u_s | 898 | 3.3101 |  |  |  |  |  |  |  |  | Yes |  |  |  |
| Desulfotomaculum_u_s | 1562 | 2.3111 |  |  |  |  |  |  |  |  | Yes |  |  |  |
| Desulfovibrio sp. Dsv1 | 1292024 | 3.0647 |  |  |  |  |  |  |  |  | Yes |  | 22170420, 23555659 | 3358035, 3608642 |
| Desulfovibrio_u_s | 872 | 3.0647 |  |  |  |  |  |  |  |  | Yes |  |  |  |
| Dialister_u_s | 39948 | 1.9173 |  |  |  |  |  |  |  |  | Yes |  |  |  |
| Diaphorobacter_u_s | 238749 | 4.0151 |  |  |  |  |  |  |  |  | Yes |  |  |  |
| Dictyoglomus_u_s | 13 | 1.8674 |  |  |  |  |  |  |  |  | Yes |  |  |  |
| Dietzia_u_s | 37914 | 3.7203 |  |  |  |  |  |  |  |  | Yes |  |  |  |
| Dorea_u_s | 189330 | 2.9208 |  |  |  |  |  |  |  |  | Yes |  |  |  |
| Duncaniella_u_s | 2518495 | 2.7926 |  |  |  |  |  |  |  |  | Yes |  |  |  |
| Dyadobacter_u_s | 120831 | 7.1556 |  |  |  |  |  |  |  |  | Yes |  |  |  |
| Edwardsiella_u_s | 635 | 4.0381 |  |  |  |  |  |  |  |  | Yes |  |  |  |
| Eikenella_u_s | 538 | 1.9434 |  |  |  |  |  |  |  |  | Yes |  |  |  |
| Elizabethkingia_u_s | 308865 | 4.0486 |  |  |  |  |  |  |  |  | Yes |  |  |  |
| Enhydrobacter_u_s | 212791 | 2.3768 |  |  |  |  |  |  |  |  | Yes |  |  |  |
| Enterobacter_u_s | 547 | 4.9030 |  |  |  |  |  |  |  |  | Yes |  |  |  |
| Enterobacteriaceae_u_s | 543 | 4.7450 |  |  |  |  |  |  |  |  | Yes |  |  |  |
| Enterococcus_u_s | 1350 | 2.9768 |  |  |  |  |  |  |  |  | Yes |  |  |  |
| Enterorhabdus_u_s | 580024 | 2.7930 |  |  |  |  |  |  |  |  | Yes |  |  |  |
| Erysipelotrichaceae bacterium 6_1_45 | 469614 | 4.9072 |  |  |  |  |  |  |  |  | Yes |  |  |  |
| Escherichia_u_s | 561 | 4.8364 |  |  |  |  |  |  |  |  | Yes |  |  |  |
| Eubacterium_u_s | 1730 | 2.2532 |  |  |  |  |  |  |  |  | Yes |  |  |  |
| Exiguobacterium_u_s | 33986 | 2.9343 |  |  |  |  |  |  |  |  | Yes |  |  |  |
| Facklamia_u_s | 66831 | 2.0751 |  |  |  |  |  |  |  |  | Yes |  |  |  |
| Faecalibacterium_u_s | 216851 | 2.2152 |  |  |  |  |  |  |  |  | Yes |  |  |  |
| Fibrobacter_u_s | 832 | 3.1859 |  |  |  |  |  |  |  |  | Yes |  |  |  |
| Finegoldia_u_s | 150022 | 1.8352 |  |  |  |  |  |  |  |  | Yes |  |  |  |
| Firmicutes_u_s | 1239 | 2.2927 |  |  |  |  |  |  |  |  | Yes |  |  |  |
| Flammeovirga_u_s | 59739 | 7.7251 |  |  |  |  |  |  |  |  | Yes |  |  |  |
| Flavihumibacter_u_s | 1004301 | 4.4383 |  |  |  |  |  |  |  |  | Yes |  |  |  |
| Flavobacterium_u_s | 237 | 3.9180 |  |  |  |  |  |  |  |  | Yes |  |  |  |
| Franconibacter_u_s | 1649295 | 3.9048 |  |  |  |  |  |  |  |  | Yes |  |  |  |
| Fusobacterium sp. CM1 | 936561 | 2.1272 |  |  |  |  |  |  |  |  | Yes |  |  |  |
| Fusobacterium sp. CM21 | 936562 | 2.1272 |  |  |  |  |  |  |  |  | Yes |  |  |  |
| Fusobacterium sp. CM22 | 936563 | 2.1272 |  |  |  |  |  |  |  |  | Yes |  |  |  |
| Fusobacterium sp. OBRC1 | 1032505 | 2.1272 |  |  |  |  |  |  |  |  | Yes |  |  |  |
| Fusobacterium sp. oral taxon 370 | 712288 | 2.1272 |  |  |  |  |  |  |  |  | Yes |  |  |  |
| Fusobacterium_u_s | 848 | 2.1272 |  |  |  |  |  |  |  |  | Yes |  |  |  |
| Gallibacterium_u_s | 155493 | 2.4537 |  |  |  |  |  |  |  |  | Yes |  |  |  |
| Gardnerella_u_s | 2701 | 1.5945 |  |  |  |  |  |  |  |  | Yes |  |  |  |
| Gemella_u_s | 1378 | 1.7697 |  |  |  |  |  |  |  |  | Yes |  |  |  |
| Gemmata_u_s | 113 | 4.4676 |  |  |  |  |  |  |  |  | Yes |  |  |  |
| Gemmatimonas_u_s | 173479 | 3.8565 |  |  |  |  |  |  |  |  | Yes |  |  |  |
| Geoalkalibacter_u_s | 392332 | 3.1221 |  |  |  |  |  |  |  |  | Yes |  |  |  |
| Geobacillus_u_s | 129337 | 3.4776 |  |  |  |  |  |  |  |  | Yes |  |  |  |
| Geobacter_u_s | 28231 | 3.5512 |  |  |  |  |  |  |  |  | Yes |  |  |  |
| Geodermatophilus_u_s | 1860 | 4.7254 |  |  |  |  |  |  |  |  | Yes |  |  |  |
| Gloeobacter_u_s | 33071 | 4.5335 |  |  |  |  |  |  |  |  | Yes |  |  |  |
| Glutamicibacter_u_s | 1742989 | 3.5083 |  |  |  |  |  |  |  |  | Yes |  |  |  |
| Glycomyces_u_s | 58113 | 5.1163 |  |  |  |  |  |  |  |  | Yes |  |  |  |
| Gramella_u_s | 292691 | 3.4816 |  |  |  |  |  |  |  |  | Yes |  |  |  |
| Granulicatella_u_s | 117563 | 1.9275 |  |  |  |  |  |  |  |  | Yes |  |  |  |
| Grimontia_u_s | 246861 | 5.2991 |  |  |  |  |  |  |  |  | Yes |  |  |  |
| Haemophilus sp. oral taxon 851 | 762964 | 1.9740 |  |  |  |  |  |  |  |  | Yes |  |  |  |
| Haemophilus_u_s | 724 | 1.9740 |  |  |  |  |  |  |  |  | Yes |  |  |  |
| Hafnia_u_s | 568 | 4.7078 |  |  |  |  |  |  |  |  | Yes |  |  |  |
| Haladaptatus_u_s | 367188 | 5.0446 |  |  |  |  |  |  |  |  | Yes |  |  |  |
| Halanaerobium_u_s | 2330 | 2.5123 |  |  |  |  |  |  |  |  | Yes |  |  |  |
| Halococcus_u_s | 2249 | 3.8734 |  |  |  |  |  |  |  |  | Yes |  |  |  |
| Haloferax sp. ATCC BAA-644 | 1227462 | 3.8308 |  |  |  |  |  |  |  |  | Yes |  |  |  |
| Haloferax sp. ATCC BAA-645 | 1227463 | 3.8308 |  |  |  |  |  |  |  |  | Yes |  |  |  |
| Halomonas_u_s | 2745 | 3.9306 |  |  |  |  |  |  |  |  | Yes |  |  |  |
| Helicobacter_u_s | 209 | 1.8792 |  |  |  |  |  |  |  |  | Yes |  |  |  |
| Herbaspirillum_u_s | 963 | 5.5529 |  |  |  |  |  |  |  |  | Yes |  |  |  |
| Holospora_u_s | 44747 | 1.4303 |  |  |  |  |  |  |  |  | Yes |  |  |  |
| Hymenobacter sp. APR13 | 1356852 | 5.0239 |  |  |  |  |  |  |  |  | Yes |  |  |  |
| Idiomarina_u_s | 135575 | 2.3359 |  |  |  |  |  |  |  |  | Yes |  |  |  |
| Ilumatobacter_u_s | 682522 | 2.4406 |  |  |  |  |  |  |  |  | Yes |  |  |  |
| Janibacter_u_s | 53457 | 3.6378 |  |  |  |  |  |  |  |  | Yes |  |  |  |
| Janthinobacterium sp. RA13 | 1502762 | 6.3122 |  |  |  |  |  |  |  |  | Yes |  |  |  |
| Janthinobacterium_u_s | 29580 | 6.3122 |  |  |  |  |  |  |  |  | Yes |  |  |  |
| Jiella_u_s | 1775688 | 5.0052 |  |  |  |  |  |  |  |  | Yes |  |  |  |
| Jonesia_u_s | 43673 | 2.8939 |  |  |  |  |  |  |  |  | Yes |  |  |  |
| Jonquetella sp. BV3C21 | 1111126 | 1.6543 |  |  |  |  |  |  |  |  | Yes |  |  |  |
| Jonquetella_u_s | 428711 | 1.6543 |  |  |  |  |  |  |  |  | Yes |  |  |  |
| Kibdelosporangium_u_s | 2029 | 11.7523 |  |  |  |  |  |  |  |  | Yes |  |  |  |
| Kingella_u_s | 32257 | 1.7020 |  |  |  |  |  |  |  |  | Yes |  |  |  |
| Klebsiella_u_s | 570 | 5.4679 |  |  |  |  |  |  |  |  | Yes |  |  |  |
| Kocuria sp. UCD-OTCP | 1292021 | 2.8428 |  |  |  |  |  |  |  |  | Yes |  |  |  |
| Kocuria_u_s | 57493 | 2.8428 |  |  |  |  |  |  |  |  | Yes |  |  |  |
| Kosakonia_u_s | 1330547 | 5.1338 |  |  |  |  |  |  |  |  | Yes |  |  |  |
| Kribbella_u_s | 182639 | 8.5773 |  |  |  |  |  |  |  |  | Yes |  |  |  |
| Ktedonobacteria_u_s | 388447 | 3.3013 |  |  |  |  |  |  |  |  | Yes |  |  |  |
| Kurthia_u_s | 1649 | 3.1037 |  |  |  |  |  |  |  |  | Yes |  |  |  |
| Kutzneria_u_s | 43356 | 11.6465 |  |  |  |  |  |  |  |  | Yes |  |  |  |
| Lachnoanaerobaculum sp. ICM7 | 936594 | 2.7800 |  |  |  |  |  |  |  |  | Yes |  |  |  |
| Lachnoanaerobaculum sp. MSX33 | 936596 | 2.8711 |  |  |  |  |  |  |  |  | Yes |  |  |  |
| Lachnoanaerobaculum sp. OBRC5-5 | 936595 | 2.2809 |  |  |  |  |  |  |  |  | Yes |  |  |  |
| Lachnoanaerobaculum_u_s | 1164882 | 2.2809 |  |  |  |  |  |  |  |  | Yes |  |  |  |
| Lachnoclostridium_u_s | 1164882 | 2.3835 |  |  |  |  |  |  |  |  | Yes |  |  |  |
| Lachnospiraceae bacterium AC2012 | 1392494 | 2.5554 |  |  |  |  |  |  |  |  | Yes |  |  |  |
| Lachnospiraceae bacterium oral taxon 082 | 712976 | 2.9458 |  |  |  |  |  |  |  |  | Yes |  |  |  |
| Lachnospiraceae bacterium oral taxon 500 | 712991 | 3.2447 |  |  |  |  |  |  |  |  | Yes |  |  |  |
| Lactobacillus_u_s | 1578 | 2.3488 |  |  |  |  |  |  |  |  | Yes |  |  |  |
| Lactococcus_u_s | 1357 | 2.2975 |  |  |  |  |  |  |  |  | Yes |  |  |  |
| Leptospira_u_s | 171 | 4.0024 |  |  |  |  |  |  |  |  | Yes |  |  |  |
| Leptotrichia sp. oral taxon 215 | 712359 | 2.3543 |  |  |  |  |  |  |  |  | Yes |  |  |  |
| Leptotrichia sp. oral taxon 225 | 671213 | 2.3543 |  |  |  |  |  |  |  |  | Yes |  |  |  |
| Leptotrichia sp. oral taxon 879 | 1227267 | 2.3543 |  |  |  |  |  |  |  |  | Yes |  |  |  |
| Leptotrichia_u_s | 32067 | 2.3543 |  |  |  |  |  |  |  |  | Yes |  |  |  |
| Leucobacter_u_s | 55968 | 3.1846 |  |  |  |  |  |  |  |  | Yes |  |  |  |
| Leuconostoc_u_s | 1243 | 1.6334 |  |  |  |  |  |  |  |  | Yes |  |  |  |
| Limnohabitans_u_s | 665874 | 3.3836 |  |  |  |  |  |  |  |  | Yes |  |  |  |
| Luteimonas_u_s | 83614 | 3.4016 |  |  |  |  |  |  |  |  | Yes |  |  |  |
| Lysobacter_u_s | 68 | 4.0190 |  |  |  |  |  |  |  |  | Yes |  |  |  |
| Mannheimia_u_s | 75984 | 2.2954 |  |  |  |  |  |  |  |  | Yes |  |  |  |
| Marinobacterium_u_s | 48075 | 4.0308 |  |  |  |  |  |  |  |  | Yes |  |  |  |
| Marmoricola_u_s | 86795 | 4.1083 |  |  |  |  |  |  |  |  | Yes |  |  |  |
| Massilia_u_s | 149698 | 6.0206 |  |  |  |  |  |  |  |  | Yes |  |  |  |
| Megamonas_u_s | 158846 | 2.2920 |  |  |  |  |  |  |  |  | Yes |  |  |  |
| Megasphaera sp. BV3C16-1 | 1111454 | 2.1760 |  |  |  |  |  |  |  |  | Yes |  |  |  |
| Megasphaera_u_s | 906 | 2.1760 |  |  |  |  |  |  |  |  | Yes |  |  |  |
| Mesorhizobium_u_s | 68287 | 6.6391 |  |  |  |  |  |  |  |  | Yes |  |  |  |
| Metakosakonia_u_s | 2055876 | 5.7995 |  |  |  |  |  |  |  |  | Yes |  |  |  |
| Methylibium_u_s | 316612 | 4.6163 |  |  |  |  |  |  |  |  | Yes |  |  |  |
| Methylobacterium_u_s | 407 | 5.5055 |  |  |  |  |  |  |  |  | Yes |  |  |  |
| Methylophilus_u_s | 16 | 2.9403 |  |  |  |  |  |  |  |  | Yes |  |  |  |
| Methylopila_u_s | 61653 | 4.4021 |  |  |  |  |  |  |  |  | Yes |  |  |  |
| Methylotenera_u_s | 359407 | 1.8100 |  |  |  |  |  |  |  |  | Yes |  |  |  |
| Methyloversatilis_u_s | 378210 | 2.8833 |  |  |  |  |  |  |  |  | Yes |  |  |  |
| Microbacterium_u_s | 33882 | 3.5262 |  |  |  |  |  |  |  |  | Yes |  |  |  |
| Micrococcus_u_s | 1269 | 2.5031 |  |  |  |  |  |  |  |  | Yes |  |  |  |
| Micromonospora_u_s | 1873 | 6.8463 |  |  |  |  |  |  |  |  | Yes |  |  |  |
| Microvirga_u_s | 186650 | 4.7281 |  |  |  |  |  |  |  |  | Yes |  |  |  |
| Mitsuokella_u_s | 52225 | 2.2285 |  |  |  |  |  |  |  |  | Yes |  |  |  |
| Mobilicoccus_u_s | 984996 | 3.4498 |  |  |  |  |  |  |  |  | Yes |  |  |  |
| Mobiluncus_u_s | 2050 | 2.2918 |  |  |  |  |  |  |  |  | Yes |  |  |  |
| Modestobacter_u_s | 88138 | 4.8434 |  |  |  |  |  |  |  |  | Yes |  |  |  |
| Mogibacterium_u_s | 86331 | 1.8885 |  |  |  |  |  |  |  |  | Yes |  |  |  |
| Moraxella_u_s | 475 | 2.1118 |  |  |  |  |  |  |  |  | Yes |  |  |  |
| Moritella_u_s | 58050 | 4.8364 |  |  |  |  |  |  |  |  | Yes |  |  |  |
| Muribaculum_u_s | 1918540 | 2.7640 |  |  |  |  |  |  |  |  | Yes |  |  |  |
| Muricauda_u_s | 111500 | 4.0386 |  |  |  |  |  |  |  |  | Yes |  |  |  |
| Mycobacterium_u_s | 1763 | 5.6167 |  |  |  |  |  |  |  |  | Yes |  |  |  |
| Mycolicibacterium _u_s | 1866885 | 6.2236 |  |  |  |  |  |  |  |  | Yes |  |  |  |
| Mycolicibacterium_u_s | 1866885 | 6.2623 |  |  |  |  |  |  |  |  | Yes |  |  |  |
| Nakamurella_u_s | 53460 | 4.7150 |  |  |  |  |  |  |  |  | Yes |  |  |  |
| Neisseria sp. GT4A_CT1 | 665946 | 2.3144 |  |  |  |  |  |  |  |  | Yes |  |  |  |
| Neisseria sp. oral taxon 014 | 641148 | 2.3144 |  |  |  |  |  |  |  |  | Yes |  |  |  |
| Neisseria sp. oral taxon 020 | 712401 | 2.3144 |  |  |  |  |  |  |  |  | Yes |  |  |  |
| Neisseria_u_s | 482 | 2.3144 |  |  |  |  |  |  |  |  | Yes |  |  |  |
| Neorhizobium_u_s | 1525371 | 6.2149 |  |  |  |  |  |  |  |  | Yes |  |  |  |
| Neorickettsia_u_s | 33993 | 0.8592 |  |  |  |  |  |  |  |  | Yes |  |  |  |
| Nesterenkonia_u_s | 57494 | 2.9261 |  |  |  |  |  |  |  |  | Yes |  |  |  |
| Nitrobacter_u_s | 911 | 3.7940 |  |  |  |  |  |  |  |  | Yes |  |  |  |
| Nitrosococcus_u_s | 1227 | 3.7658 |  |  |  |  |  |  |  |  | Yes |  |  |  |
| Nocardioides_u_s | 1839 | 4.3780 |  |  |  |  |  |  |  |  | Yes |  |  |  |
| Nocardiopsis_u_s | 2013 | 6.2094 |  |  |  |  |  |  |  |  | Yes |  |  |  |
| Novosphingobium_u_s | 165696 | 4.6270 |  |  |  |  |  |  |  |  | Yes |  |  |  |
| Ochrobactrum_u_s | 528 | 4.8343 |  |  |  |  |  |  |  |  | Yes |  |  |  |
| Olsenella sp. oral taxon 809 | 661086 | 2.1408 |  |  |  |  |  |  |  |  | Yes |  |  |  |
| Olsenella_u_s | 133925 | 2.1408 |  |  |  |  |  |  |  |  | Yes |  |  |  |
| Oribacterium sp. oral taxon 078 | 652706 | 2.2411 |  |  |  |  |  |  |  |  | Yes |  |  |  |
| Oribacterium sp. oral taxon 108 | 712414 | 2.2411 |  |  |  |  |  |  |  |  | Yes |  |  |  |
| Oribacterium_u_s | 265975 | 2.2411 |  |  |  |  |  |  |  |  | Yes |  |  |  |
| Ornithinimicrobium_u_s | 125287 | 3.8505 |  |  |  |  |  |  |  |  | Yes |  |  |  |
| Oscillibacter sp. ER4 | 1519439 | 2.0914 |  |  |  |  |  |  |  |  | Yes |  |  |  |
| Oscillibacter_u_s | 459786 | 2.0914 |  |  |  |  |  |  |  |  | Yes |  |  |  |
| Ottowia_u_s | 219181 | 2.8306 |  |  |  |  |  |  |  |  | Yes |  |  |  |
| Pantoea sp. PSNIH1 | 1484158 | 4.8670 |  |  |  |  |  |  |  |  | Yes |  |  |  |
| Pantoea_u_s | 53335 | 4.8670 |  |  |  |  |  |  |  |  | Yes |  |  |  |
| Parabacteroides_u_s | 375288 | 4.3247 |  |  |  |  |  |  |  |  | Yes |  |  |  |
| Paracoccus_u_s | 265 | 3.8116 |  |  |  |  |  |  |  |  | Yes |  |  |  |
| Parvimonas sp. oral taxon 110 | 671230 | 1.5419 |  |  |  |  |  |  |  |  | Yes |  |  |  |
| Parvimonas sp. oral taxon 393 | 713008 | 1.5419 |  |  |  |  |  |  |  |  | Yes |  |  |  |
| Parvimonas_u_s | 543311 | 1.5419 |  |  |  |  |  |  |  |  | Yes |  |  |  |
| Patulibacter_u_s | 361607 | 5.0283 |  |  |  |  |  |  |  |  | Yes |  |  |  |
| Pedobacter_u_s | 84567 | 5.1522 |  |  |  |  |  |  |  |  | Yes |  |  |  |
| Pelosinus_u_s | 365348 | 4.3178 |  |  |  |  |  |  |  |  | Yes |  |  |  |
| Peptoclostridium_u_s | 1481960 | 2.5676 |  |  |  |  |  |  |  |  | Yes |  |  |  |
| Peptoniphilus sp. ChDC B134 | 1354300 | 1.4998 |  |  |  |  |  |  |  |  | Yes |  |  |  |
| Peptoniphilus sp. oral taxon 836 | 671216 | 1.6740 |  |  |  |  |  |  |  |  | Yes |  |  |  |
| Peptoniphilus_u_s | 162289 | 1.6740 |  |  |  |  |  |  |  |  | Yes |  |  |  |
| Peptostreptococcaceae bacterium AS15 | 936556 | 2.6546 |  |  |  |  |  |  |  |  | Yes |  |  |  |
| Peptostreptococcaceae bacterium oral taxon 113 | 1321783 | 2.0883 |  |  |  |  |  |  |  |  | Yes |  |  |  |
| Peptostreptococcus sp. MV1 | 1219626 | 2.0635 |  |  |  |  |  |  |  |  | Yes |  |  |  |
| Peptostreptococcus_u_s | 1257 | 2.0635 |  |  |  |  |  |  |  |  | Yes |  |  |  |
| Phascolarctobacterium sp. CAG:207 | 1262914 | 1.7532 |  |  |  |  |  |  |  |  | Yes |  |  |  |
| Phyllobacterium_u_s | 28100 | 4.7293 |  |  |  |  |  |  |  |  | Yes |  |  |  |
| Planococcus_u_s | 1372 | 3.6255 |  |  |  |  |  |  |  |  | Yes |  |  |  |
| Polaribacter_u_s | 52959 | 3.2412 |  |  |  |  |  |  |  |  | Yes |  |  |  |
| Polaromonas_u_s | 52972 | 4.5195 |  |  |  |  |  |  |  |  | Yes |  |  |  |
| Pontibacter_u_s | 323449 | 4.6238 |  |  |  |  |  |  |  |  | Yes |  |  |  |
| Porphyromonadaceae bacterium COT-184 OH4590 | 1517682 | 2.3925 |  |  |  |  |  |  |  |  | Yes |  |  |  |
| Porphyromonas sp. KLE 1280 | 997829 | 2.0291 |  |  |  |  |  |  |  |  | Yes |  |  |  |
| Porphyromonas sp. oral taxon 278 | 712437 | 2.0291 |  |  |  |  |  |  |  |  | Yes |  |  |  |
| Porphyromonas sp. oral taxon 279 | 712438 | 2.0291 |  |  |  |  |  |  |  |  | Yes |  |  |  |
| Porphyromonas_u_s | 836 | 2.0291 |  |  |  |  |  |  |  |  | Yes |  |  |  |
| Prevotella conceptionensis | 340486 | 4.0801 |  |  |  |  |  |  |  |  | Yes |  |  |  |
| Prevotella sp. C561 | 563031 | 2.7616 |  |  |  |  |  |  |  |  | Yes |  |  |  |
| Prevotella sp. F0091 | 1227276 | 2.7616 |  |  |  |  |  |  |  |  | Yes |  |  |  |
| Prevotella sp. HJM029 | 1433844 | 2.7616 |  |  |  |  |  |  |  |  | Yes |  |  |  |
| Prevotella sp. ICM33 | 1161412 | 2.7616 |  |  |  |  |  |  |  |  | Yes |  |  |  |
| Prevotella sp. MSX73 | 1032506 | 2.7616 |  |  |  |  |  |  |  |  | Yes |  |  |  |
| Prevotella sp. oral taxon 299 | 652716 | 2.7616 |  |  |  |  |  |  |  |  | Yes |  |  |  |
| Prevotella sp. oral taxon 306 | 712461 | 2.7616 |  |  |  |  |  |  |  |  | Yes |  |  |  |
| Prevotella sp. oral taxon 317 | 652721 | 2.7616 |  |  |  |  |  |  |  |  | Yes |  |  |  |
| Prevotella sp. oral taxon 472 | 655809 | 2.7616 |  |  |  |  |  |  |  |  | Yes |  |  |  |
| Prevotella sp. oral taxon 473 | 712469 | 2.7616 |  |  |  |  |  |  |  |  | Yes |  |  |  |
| Prevotella_u_s | 838 | 2.7616 |  |  |  |  |  |  |  |  | Yes |  |  |  |
| Promicromonospora_u_s | 43676 | 6.4091 |  |  |  |  |  |  |  |  | Yes |  |  |  |
| Propionibacterium sp. 434-HC2 | 936048 | 1.9999 |  |  |  |  |  |  |  |  | Yes |  |  |  |
| Propionibacterium sp. 5_U_42AFAA | 450748 | 1.9999 |  |  |  |  |  |  |  |  | Yes |  |  |  |
| Propionibacterium sp. DORA_15 | 1403938 | 2.6930 |  |  |  |  |  |  |  |  | Yes |  |  |  |
| Propionibacterium sp. HGH0353 | 1203571 | 1.9999 |  |  |  |  |  |  |  |  | Yes |  |  |  |
| Propionibacterium sp. KPL1844 | 1203573 | 1.9999 |  |  |  |  |  |  |  |  | Yes |  |  |  |
| Propionibacterium sp. KPL2009 | 1203635 | 1.9999 |  |  |  |  |  |  |  |  | Yes |  |  |  |
| Propionibacterium sp. oral taxon 192 | 671222 | 1.9999 |  |  |  |  |  |  |  |  | Yes |  |  |  |
| Propionibacterium_u_s | 1743 | 1.9999 |  |  |  |  |  |  |  |  | Yes |  |  |  |
| Proteus_u_s | 583 | 3.8897 |  |  |  |  |  |  |  |  | Yes |  |  |  |
| Pseudomonas sp. Ag1 | 1197727 | 6.1780 |  |  |  |  |  |  |  |  | Yes |  |  |  |
| Pseudomonas sp. CBZ-4 | 1163065 | 6.1780 |  |  |  |  |  |  |  |  | Yes |  |  |  |
| Pseudomonas_u_s | 286 | 6.1780 |  |  |  |  |  |  |  |  | Yes |  |  |  |
| Pseudonocardia_u_s | 1847 | 6.5274 |  |  |  |  |  |  |  |  | Yes |  |  |  |
| Pseudoxanthomonas_u_s | 83618 | 4.3851 |  |  |  |  |  |  |  |  | Yes |  |  |  |
| Psychrobacter_u_s | 497 | 3.1201 |  |  |  |  |  |  |  |  | Yes |  |  |  |
| Psychroflexus_u_s | 83612 | 2.6513 |  |  |  |  |  |  |  |  | Yes |  |  |  |
| Pusillimonas_u_s | 305976 | 3.3014 |  |  |  |  |  |  |  |  | Yes |  |  |  |
| Quadrisphaera_u_s | 317661 | 4.2015 |  |  |  |  |  |  |  |  | Yes |  |  |  |
| Ralstonia sp. AU12-08 | 1235457 | 5.2548 |  |  |  |  |  |  |  |  | Yes |  |  |  |
| Ralstonia_u_s | 48736 | 5.3658 |  |  |  |  |  |  |  |  | Yes |  |  |  |
| Rheinheimera_u_s | 67575 | 4.1526 |  |  |  |  |  |  |  |  | Yes |  |  |  |
| Rhizobium_u_s | 379 | 6.3427 |  |  |  |  |  |  |  |  | Yes |  |  |  |
| Rhodococcus_u_s | 1827 | 5.7057 |  |  |  |  |  |  |  |  | Yes |  |  |  |
| Rhodopirellula_u_s | 265488 | 6.5559 |  |  |  |  |  |  |  |  | Yes |  |  |  |
| Rhodopseudomonas_u_s | 1073 | 5.4050 |  |  |  |  |  |  |  |  | Yes |  |  |  |
| Roseburia_u_s | 841 | 2.9104 |  |  |  |  |  |  |  |  | Yes |  |  |  |
| Roseiflexus_u_s | 120961 | 5.8016 |  |  |  |  |  |  |  |  | Yes |  |  |  |
| Roseomonas_u_s | 125216 | 5.8029 |  |  |  |  |  |  |  |  | Yes |  |  |  |
| Rothia_u_s | 32207 | 2.3045 |  |  |  |  |  |  |  |  | Yes |  |  |  |
| Ruminococcus sp. 5_1_39BFAA | 457412 | 2.1184 |  |  |  |  |  |  |  |  | Yes |  |  |  |
| Ruminococcus_u_s | 1263 | 2.1300 |  |  |  |  |  |  |  |  | Yes |  |  |  |
| Runella_u_s | 105 | 7.1365 |  |  |  |  |  |  |  |  | Yes |  |  |  |
| Saccharibacter_u_s | 231052 | 2.1793 |  |  |  |  |  |  |  |  | Yes |  |  |  |
| Saccharopolyspora_u_s | 1835 | 6.3306 |  |  |  |  |  |  |  |  | Yes |  |  |  |
| Saccharothrix_u_s | 2071 | 9.3040 |  |  |  |  |  |  |  |  | Yes |  |  |  |
| Salinicoccus_u_s | 45669 | 2.4390 |  |  |  |  |  |  |  |  | Yes |  |  |  |
| Salinispora_u_s | 168694 | 5.4510 |  |  |  |  |  |  |  |  | Yes |  |  |  |
| Salmonella_u_s | 590 | 4.8647 |  |  |  |  |  |  |  |  | Yes |  |  |  |
| Sandarakinorhabdus_u_s | 362865 | 3.1986 |  |  |  |  |  |  |  |  | Yes |  |  |  |
| Scardovia_u_s | 196081 | 1.5084 |  |  |  |  |  |  |  |  | Yes |  |  |  |
| Sedimentibacter sp. B4 | 304766 | 3.6190 |  |  |  |  |  |  |  |  | Yes |  |  |  |
| Segniliparus_u_s | 286801 | 3.3726 |  |  |  |  |  |  |  |  | Yes |  |  |  |
| Selenomonas sp. CM52 | 936381 | 2.5583 |  |  |  |  |  |  |  |  | Yes |  |  |  |
| Selenomonas sp. F0473 | 999423 | 2.5583 |  |  |  |  |  |  |  |  | Yes |  |  |  |
| Selenomonas sp. FOBRC6 | 936572 | 2.5583 |  |  |  |  |  |  |  |  | Yes |  |  |  |
| Selenomonas sp. FOBRC9 | 936573 | 2.5583 |  |  |  |  |  |  |  |  | Yes |  |  |  |
| Selenomonas sp. oral taxon 137 | 712531 | 2.5583 |  |  |  |  |  |  |  |  | Yes |  |  |  |
| Selenomonas sp. oral taxon 138 | 712532 | 2.5583 |  |  |  |  |  |  |  |  | Yes |  |  |  |
| Selenomonas sp. oral taxon 149 | 712535 | 2.5583 |  |  |  |  |  |  |  |  | Yes |  |  |  |
| Selenomonas sp. oral taxon 892 | 1321785 | 2.5583 |  |  |  |  |  |  |  |  | Yes |  |  |  |
| Selenomonas_u_s | 970 | 2.5583 |  |  |  |  |  |  |  |  | Yes |  |  |  |
| Serinicoccus_u_s | 265976 | 3.5210 |  |  |  |  |  |  |  |  | Yes |  |  |  |
| Serratia_u_s | 613 | 5.3084 |  |  |  |  |  |  |  |  | Yes |  |  |  |
| Shewanella_u_s | 22 | 4.8611 |  |  |  |  |  |  |  |  | Yes |  |  |  |
| Shigella_u_s | 620 | 4.4866 |  |  |  |  |  |  |  |  | Yes |  |  |  |
| Shuttleworthia sp. MSX8B | 936574 | 2.1026 |  |  |  |  |  |  |  |  | Yes |  |  |  |
| Shuttleworthia_u_s | 177971 | 2.1026 |  |  |  |  |  |  |  |  | Yes |  |  |  |
| Slackia sp. CM382 | 1111137 | 2.0414 |  |  |  |  |  |  |  |  | Yes |  |  |  |
| Slackia_u_s | 84108 | 2.0309 |  |  |  |  |  |  |  |  | Yes |  |  |  |
| Solirubrobacter_u_s | 207599 | 5.8621 |  |  |  |  |  |  |  |  | Yes |  |  |  |
| Sphingobacterium_u_s | 28453 | 5.2037 |  |  |  |  |  |  |  |  | Yes |  |  |  |
| Sphingobium_u_s | 165695 | 4.4793 |  |  |  |  |  |  |  |  | Yes |  |  |  |
| Sphingomonas sp. Ant H11 | 1564113 | 4.0412 |  |  |  |  |  |  |  |  | Yes |  |  |  |
| Sphingomonas_u_s | 13687 | 4.0412 |  |  |  |  |  |  |  |  | Yes |  |  |  |
| Sphingopyxis_u_s | 165697 | 4.3074 |  |  |  |  |  |  |  |  | Yes |  |  |  |
| Spirosoma_u_s | 107 | 8.9919 |  |  |  |  |  |  |  |  | Yes |  |  |  |
| Spongiibacter_u_s | 630749 | 3.4074 |  |  |  |  |  |  |  |  | Yes |  |  |  |
| Staphylococcus sp. DORA_6_22 | 1403935 | 2.5350 |  |  |  |  |  |  |  |  | Yes |  |  |  |
| Staphylococcus sp. M0480 | 1388318 | 2.5350 |  |  |  |  |  |  |  |  | Yes |  |  |  |
| Staphylococcus sp. MDS7B | 1209359 | 2.5350 |  |  |  |  |  |  |  |  | Yes |  |  |  |
| Staphylococcus_u_s | 1279 | 2.5350 |  |  |  |  |  |  |  |  | Yes |  |  |  |
| Stenotrophomonas sp. SKA14 | 391601 | 4.4388 |  |  |  |  |  |  |  |  | Yes |  |  |  |
| Stenotrophomonas_u_s | 40323 | 4.4388 |  |  |  |  |  |  |  |  | Yes |  |  |  |
| Streptococcus sp. 2_1_36FAA | 469609 | 2.0368 |  |  |  |  |  |  |  |  | Yes |  |  |  |
| Streptococcus sp. ACC21 | 1161413 | 2.0545 |  |  |  |  |  |  |  |  | Yes |  |  |  |
| Streptococcus sp. ACS2 | 936576 | 2.0368 |  |  |  |  |  |  |  |  | Yes |  |  |  |
| Streptococcus sp. AS14 | 936577 | 2.0368 |  |  |  |  |  |  |  |  | Yes |  |  |  |
| Streptococcus sp. AS20 | 936578 | 2.0368 |  |  |  |  |  |  |  |  | Yes |  |  |  |
| Streptococcus sp. BS21 | 1161414 | 2.0368 |  |  |  |  |  |  |  |  | Yes |  |  |  |
| Streptococcus sp. BS35b | 1105032 | 2.0368 |  |  |  |  |  |  |  |  | Yes |  |  |  |
| Streptococcus sp. C300 | 563036 | 2.0368 |  |  |  |  |  |  |  |  | Yes |  |  |  |
| Streptococcus sp. CM6 | 936580 | 2.0368 |  |  |  |  |  |  |  |  | Yes |  |  |  |
| Streptococcus sp. CM7 | 936581 | 2.0545 |  |  |  |  |  |  |  |  | Yes |  |  |  |
| Streptococcus sp. DBCMS | 1604598 | 2.0368 |  |  |  |  |  |  |  |  | Yes |  |  |  |
| Streptococcus sp. DORA_10 | 1403937 | 2.0368 |  |  |  |  |  |  |  |  | Yes |  |  |  |
| Streptococcus sp. F0441 | 999424 | 2.0368 |  |  |  |  |  |  |  |  | Yes |  |  |  |
| Streptococcus sp. GMD3S | 1169672 | 2.0368 |  |  |  |  |  |  |  |  | Yes |  |  |  |
| Streptococcus sp. GMD5S | 1169674 | 2.0368 |  |  |  |  |  |  |  |  | Yes |  |  |  |
| Streptococcus sp. HSISM1 | 1316408 | 2.0368 |  |  |  |  |  |  |  |  | Yes |  |  |  |
| Streptococcus sp. HSISS2 | 1316411 | 2.0368 |  |  |  |  |  |  |  |  | Yes |  |  |  |
| Streptococcus sp. HSISS3 | 1316412 | 2.0545 |  |  |  |  |  |  |  |  | Yes |  |  |  |
| Streptococcus sp. I-G2 | 1156431 | 2.0368 |  |  |  |  |  |  |  |  | Yes |  |  |  |
| Streptococcus sp. I-P16 | 1156433 | 2.0368 |  |  |  |  |  |  |  |  | Yes |  |  |  |
| Streptococcus sp. M143 | 563037 | 2.0368 |  |  |  |  |  |  |  |  | Yes |  |  |  |
| Streptococcus sp. M334 | 563038 | 2.0368 |  |  |  |  |  |  |  |  | Yes |  |  |  |
| Streptococcus sp. OBRC6 | 936587 | 2.0368 |  |  |  |  |  |  |  |  | Yes |  |  |  |
| Streptococcus sp. oral taxon 056 | 712620 | 2.0368 |  |  |  |  |  |  |  |  | Yes |  |  |  |
| Streptococcus sp. oral taxon 058 | 712622 | 2.0368 |  |  |  |  |  |  |  |  | Yes |  |  |  |
| Streptococcus sp. oral taxon 071 | 712630 | 2.0368 |  |  |  |  |  |  |  |  | Yes |  |  |  |
| Streptococcus sp. SR1 | 1161416 | 2.0368 |  |  |  |  |  |  |  |  | Yes |  |  |  |
| Streptococcus sp. SR4 | 1161417 | 2.0368 |  |  |  |  |  |  |  |  | Yes |  |  |  |
| Streptococcus sp. VT 162 | 1419814 | 2.0368 |  |  |  |  |  |  |  |  | Yes |  |  |  |
| Streptococcus_u_s | 1301 | 2.0368 |  |  |  |  |  |  |  |  | Yes |  |  |  |
| Streptomyces_u_s | 1883 | 8.2199 |  |  |  |  |  |  |  |  | Yes |  |  |  |
| Subdoligranulum sp. 4_3_54A2FAA | 665956 | 2.3391 |  |  |  |  |  |  |  |  | Yes |  |  |  |
| Subdoligranulum_u_s | 292632 | 2.3723 |  |  |  |  |  |  |  |  | Yes |  |  |  |
| Sulfobacillus_u_s | 28033 | 3.3587 |  |  |  |  |  |  |  |  | Yes |  |  |  |
| Sulfuricurvum_u_s | 286130 | 2.0135 |  |  |  |  |  |  |  |  | Yes |  |  |  |
| Sulfurospirillum_u_s | 57665 | 2.6041 |  |  |  |  |  |  |  |  | Yes |  |  |  |
| Sutterella_u_s | 40544 | 1.8366 |  |  |  |  |  |  |  |  | Yes |  |  |  |
| Synechocystis sp. PCC 7509 | 927677 | 3.6599 |  |  |  |  |  |  |  |  | Yes |  |  |  |
| Tannerella sp. oral taxon HOT-286 | 712710 | 2.9735 |  |  |  |  |  |  |  |  | Yes |  |  |  |
| Tannerella_u_s | 195950 | 3.0136 |  |  |  |  |  |  |  |  | Yes |  |  |  |
| Tatumella_u_s | 82986 | 3.5179 |  |  |  |  |  |  |  |  | Yes |  |  |  |
| Taylorella_u_s | 29574 | 1.6429 |  |  |  |  |  |  |  |  | Yes |  |  |  |
| Tenacibaculum_u_s | 104267 | 3.9186 |  |  |  |  |  |  |  |  | Yes |  |  |  |
| Thalassospira_u_s | 168934 | 4.3678 |  |  |  |  |  |  |  |  | Yes |  |  |  |
| Thauera_u_s | 33057 | 3.7254 |  |  |  |  |  |  |  |  | Yes |  |  |  |
| Thermanaerovibrio_u_s | 81461 | 1.8647 |  |  |  |  |  |  |  |  | Yes |  |  |  |
| Thermoactinomyces_u_s | 2023 | 2.6233 |  |  |  |  |  |  |  |  | Yes |  |  |  |
| Thermoanaerobacterium_u_s | 28895 | 2.9797 |  |  |  |  |  |  |  |  | Yes |  |  |  |
| Thermodesulfatator_u_s | 241192 | 1.2829 |  |  |  |  |  |  |  |  | Yes |  |  |  |
| Thermodesulfobacterium_u_s | 1740 | 1.5616 |  |  |  |  |  |  |  |  | Yes |  |  |  |
| Thermus_u_s | 270 | 2.2635 |  |  |  |  |  |  |  |  | Yes |  |  |  |
| Thioalkalivibrio_u_s | 106633 | 2.8203 |  |  |  |  |  |  |  |  | Yes |  |  |  |
| Thiomicrorhabdus_u_s | 2039723 | 2.6504 |  |  |  |  |  |  |  |  | Yes |  |  |  |
| Thiomonas_u_s | 32012 | 3.6094 |  |  |  |  |  |  |  |  | Yes |  |  |  |
| Treponema sp. OMZ 838 | 1539298 | 2.3518 |  |  |  |  |  |  |  |  | Yes |  |  |  |
| Treponema_u_s | 157 | 2.3518 |  |  |  |  |  |  |  |  | Yes |  |  |  |
| Tsukamurella_u_s | 2060 | 4.5034 |  |  |  |  |  |  |  |  | Yes |  |  |  |
| Turicibacter_u_s | 191303 | 2.1955 |  |  |  |  |  |  |  |  | Yes |  |  |  |
| Ureaplasma_u_s | 2129 | 0.5443 |  |  |  |  |  |  |  |  | Yes |  |  |  |
| Variovorax_u_s | 34072 | 7.0047 |  |  |  |  |  |  |  |  | Yes |  |  |  |
| Veillonella sp. 3_1_44 | 457416 | 1.9380 |  |  |  |  |  |  |  |  | Yes |  |  |  |
| Veillonella sp. 6_1_27 | 450749 | 1.9380 |  |  |  |  |  |  |  |  | Yes |  |  |  |
| Veillonella sp. DORA_B_18_19_23 | 1403933 | 1.9380 |  |  |  |  |  |  |  |  | Yes |  |  |  |
| Veillonella sp. oral taxon 158 | 671228 | 1.9380 |  |  |  |  |  |  |  |  | Yes |  |  |  |
| Veillonella_u_s | 29465 | 1.9380 |  |  |  |  |  |  |  |  | Yes |  |  |  |
| Verminephrobacter_u_s | 364316 | 4.4223 |  |  |  |  |  |  |  |  | Yes |  |  |  |
| Vibrio_u_s | 662 | 4.9726 |  |  |  |  |  |  |  |  | Yes |  |  |  |
| Xanthomonas_u_s | 338 | 4.8501 |  |  |  |  |  |  |  |  | Yes |  |  |  |
| Yersinia_u_s | 629 | 4.6433 |  |  |  |  |  |  |  |  | Yes |  |  |  |
